# Design, synthesis, molecular modelling and biological evaluation of novel 6-amino-5-cyano-2-thiopyrimidine derivatives as potent anticancer agents against leukemia and apoptotic inducers

**DOI:** 10.1080/14756366.2024.2304625

**Published:** 2024-02-13

**Authors:** Naglaa M. Ahmed, Mosaad S. Mohamed, Samir M. Awad, Rania H. Abd El-Hameed, Neama A. Abd El-tawab, Mohamed S. Gaballah, Ahmed M. Said

**Affiliations:** a Pharmaceutical Organic Chemistry Department, Helwan University, Ein-Helwan, Egypt; b Biochemistry and Molecular Biology Department, Helwan University, Ein-Helwan, Egypt; c Department of Chemistry, University at Buffalo, The State University of New York, Buffalo, USA; d Athenex Inc, Buffalo, NY, USA

**Keywords:** Pyrimidine, anticancer, Bax, Bcl_2_, p53, Caspase3, Apoptosis, PI3K

## Abstract

Herein, a novel series of 6-amino-5-cyano-2-thiopyrimidines and condensed pyrimidines analogues were prepared. All the synthesized compounds **(1a-c, 2a-c, 3a-c, 4a-r** and **5a-c)** were evaluated for *in vitro* anticancer activity by the National Cancer Institute (NCI; MD, USA) against 60 cell lines. Compound **1c** showed promising anticancer activity and was selected for the five-dose testing. Results demonstrated that compound **1c** possessed broad spectrum anti-cancer activity against the nine cancerous subpanels tested with selectivity ratio ranging from 0.7 to 39 at the GI_50_ level with high selectivity towards leukaemia. Mechanistic studies showed that Compound **1c** showed comparable activity to Duvelisib against PI3Kδ (IC_50_ = 0.0034 and 0.0025 μM, respectively) and arrested cell cycle at the S phase and displayed significant increase in the early and late apoptosis in HL60 and leukaemia SR cells. The necrosis percentage showed a significant increase from 1.13% to 3.41% in compound **1c** treated HL60 cells as well as from 1.51% to 4.72% in compound **1c** treated leukaemia SR cells. Also, compound **1c** triggered apoptosis by activating caspase 3, Bax, P53 and suppressing Bcl_2_. Moreover, **1c** revealed a good safety profile against human normal lung fibroblast cell line (WI-38 cells). Molecular analysis of Duvelisib and compound **1c** in PI3K was performed. Finally, these results suggest that 2-thiopyrimidine derivative **1c** might serve as a model for designing novel anticancer drugs in the future.

## Graphical Abstract

**Figure:**
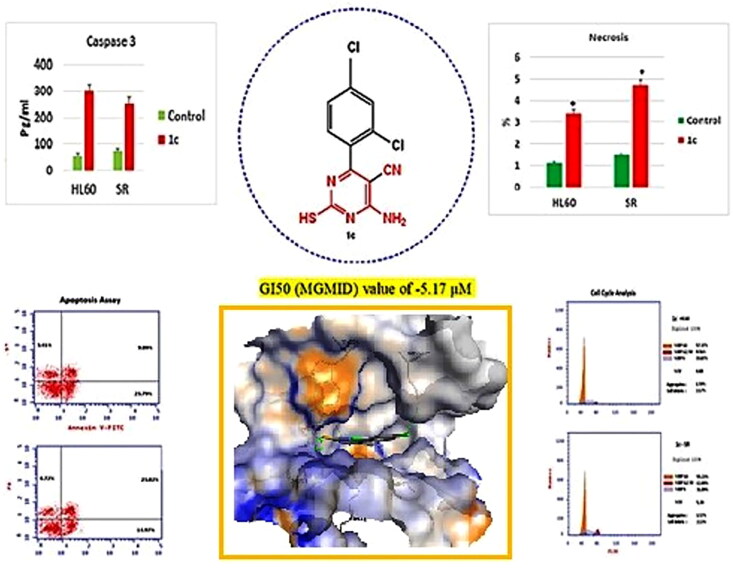

## Introduction

Cancer is a complex, fatal disease that affects several organs in the human body and poses a significant threat to human health and welfare^1–3^. Leukaemia is a common malignancy associated with an increased number of leukocytes in the blood or bone marrow^4^. It can be broadly categorised into myeloid or lymphoid lineages, according to the WHO Classification of Tumours of Haematopoietic and Lymphoid Tissues, Fourth Edition. The predominant leukaemia cells can be mature, precursor cells of various lineages, or both. Acute myeloid leukaemia (AML), acute lymphoblastic leukaemia (ALL), chronic myeloid leukaemia (CML), and chronic lymphocytic leukaemia (CLL) are the four primary subtypes of leukaemia^5^. Based on estimates from the American Cancer Society, in 2023 there will be an estimated: 59,610 new cases of leukaemia reported, and about 23,710 deaths from all forms of the disease ^6^. Based on the latest WHO data, the number of leukaemia deaths in Egypt reached 3763 in 2020, or 0.70% of all deaths.

Rapid proliferation is the characteristic that all cancers have in common^7^^.^ Due to the highly intricate and interconnected molecular pathways involved in this proliferation, multitarget cancer treatment strategies must be used^8^. Targeting cell cycle phases and checkpoints is a relatively new area for cancer treatment, and it may offer special opportunities and show promise for the advancement of cancer treatment.

Five phases of the cell cycle are recognised: G0 (gap 0), G1 (gap 1), S (DNA synthesis), G2 (gap 2), and M (mitosis). There are checkpoints in between these phases where the accuracy of DNA synthesis and the integrity of cellular components are observed. The G1/S and G2/M boundaries have two significant checkpoints^9^.

Human cancer is characterised by an abnormal cell distribution during cell cycle progression. Tumour cells have a propensity to accumulate changes in cell cycle machinery components, which results in an impaired ability to respond to DNA damage by halting cell cycle progression. Chemo resistance could be brought on by the cell cycle machinery’s diminished capacity to react to DNA damage as many anti-cancer medications are DNA damaging substances^9–11^. Therefore, a new class of medications that concentrate on characteristics of the cell cycle unique to cancer cells is required.

Apoptosis is a method of controlled cell death that allows cells with significant DNA damage to be destroyed. The notion that apoptosis might be essential for eradicating malignant cells, hyperplasia, and tumour progression was first put forth by Kerr et al^12^. in the early 1970s. Cancer cells can develop apoptosis resistance through three main known mechanisms; either by generating imbalance between pro and antiapoptotic proteins, or due to deficiency in death receptor signalling, or by reduction in the activity of the caspases^13^. As a result, cancer therapy may benefit from inducing apoptosis in cancerous cells, and malignant tumour cells must be selectively eliminated so that healthy cells can continue to proliferate. However, the capacity of tumour cells to avoid apoptosis during chemotherapy is another defining feature of cancer.

By overexpressing anti-apoptotic BCL-2 family members^14–16^ like BCL_2_, BCLX, and MCL1 and/or by having low levels of pro-apoptotic members like BAX^17^, acute leukaemia is linked to a reduction in cell sensitivity to pro-apoptotic signals. This means that leukemic cells can avoid apoptosis, which can result in chemotherapy resistance and relapse that is linked to a shorter time in which a patient will remain disease-free or die^14^^,^^15^^,^^17^^,^^18^.

Chemotherapy, which includes multi-agent chemotherapy regimens, is the standard first-line therapeutic approach for people with acute leukemia^19^. Pancreatitis, coagulopathy, hepatotoxicity, and the development of chemo resistance are just a few of the unfortunate off-target effects of multi-agent chemotherapy regimens, which ultimately lead to patient death and disease relapse^19–22^. Designing targeted therapies with lower toxicities, which reverse chemotherapy resistance, and increase acute leukaemia patient survival is currently one of the most promising anti-leukaemia strategies^14^^,^^18^.

Heterocyclic derivatives, a significant class of organic compounds, are extensively used in a wide range of pharmaceutical and industrial applications. In medicinal chemistry, pyrimidines are one of the most significant heterocyclic molecules because they exhibit exceptional pharmacological properties as necessary constituents of every cell, and thus every biological living matter^23^. Pyrimidines are present in several naturally occurring molecules such as nucleotides, nucleic acids, vitamins, coenzymes, purines, pterins, and uric acids. As a result, the wide spread use of pyrimidines is attributed to that; DNA and RNA contain them sufficiently. Medicinal chemists developed and made use of a variety of pyrimidine scaffolds to create novel therapeutics with a variety of pharmacological activities, such as anti-proliferative, antiviral, antitumor, anti-inflammatory, antibacterial, antifungal, anti-Alzheimer’s, and anti-tubercular properties^24–28^.

**Figure 1.**
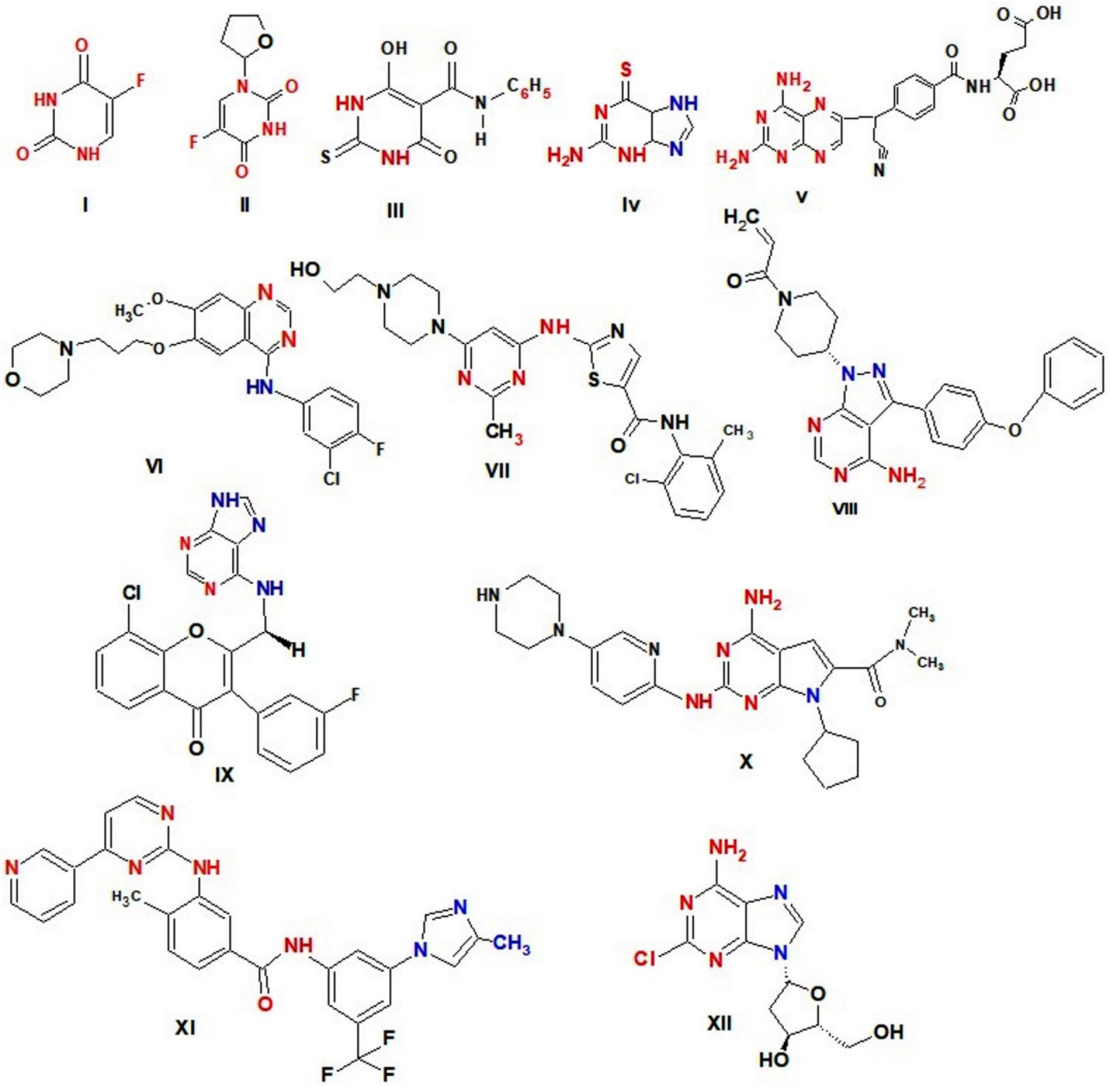
Anticancer drugs have pyrimidine ring.

Numerous drugs with pyrimidine nucleus are utilised as effective anticancer agents by a variety of modes of action, including 5-fluorouracil (5-FU) **I** as a thymidylate synthase inhibitor^29^^,^ Tegafur **II** a prodrug of 5-FU^30^, Merbarone **III** as DNA topoisomerase II (topo II) catalytic inhibitor^31^, Thioguanine **IV** as purine antagonists^32^, Pralatrexate **V** as Dihydrofolate reductase (DHFR) inhibitor^33^, Gefitinib **VI** as a potent EGFR inhibitor^34^, Dasatinib **VII** as multi-targeted of Bcr-Abl, Src family kinases inhibitor and apoptosis inducer^35^, Ibrutinib (IBR) **VIII** as Bruton’s tyrosine kinase (BTK) inhibitor^36^, Duvelisib **IX** as a potent PI3K inhibitor^37^ and was approved by FDA for the treatment of chronic lymphocytic leukaemia (CLL) and small lymphocytic lymphoma (SLL)^38^, Ribociclib **X** as Cyclin-dependent kinases (CDK) inhibitors^39^; effectively inhibits CDK_4_ and CDK_6;_ induces cell cycle arrested at the G0/G1 phase in IMR5 and BE2C cell^40^, Nilotinib **XI** as tyrosine kinase inhibitor and apoptosis inducer^41^ and Cladribine **XII** as DNA synthesis inhibitor by inhibiting adenosine deaminase^42^^,^^43^. Cladribine causes G1 phase arrest, p21 and p27 expression to rise, inhibit proliferation, induces apoptosis, and activates intrinsic and extrinsic signalling pathways in DLBCL cells^42^ (Figure 1).

Numerous pyrimidine derivatives have been prepared and tested for their potential as anticancer drugs.

**Figure 2.**
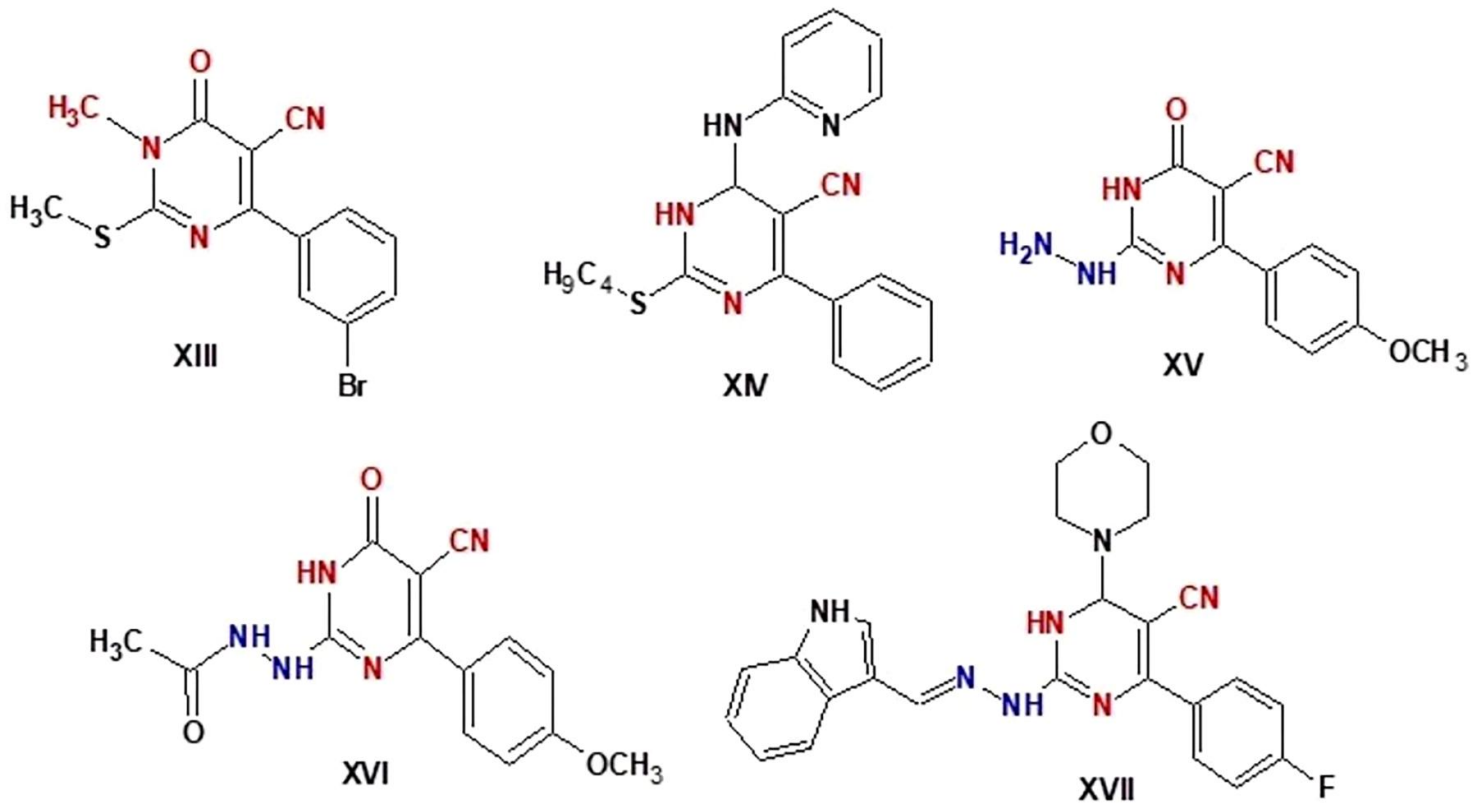
Pyrimidine -5-carbonitril derivatives as anticancer agents.

Pyrimidine-5-carbonitrile is a privileged chemical compound with a strong anticancer effect. Various pyrimidine-5-carbonitrile derivatives have been found to be cytotoxic to various leukaemia and solid tumour cell lines^44–46^. For instance, compound **XIII** showed strong anticancer activity against melanoma, leukaemia, non-small cell lung, and renal cancer^47^^.^ With IC_50_ values of 3.37, 3.04, 4.14, and 2.4 µM, respectively. Compound **XIV** demonstrated 4.5- to 8.4-fold erlotinib activity against HCT-116, HepG-2, MCF-7, and A549 cells and showed high activity against both EGFRWT and mutant EGFRT790M with IC_50_ values of 0.09 and 4.03 µM, respectively. In HCT-116, HepG-2, and MCF-7 cells, compound **XIV** can significantly increase apoptosis and arrest the cell cycle at the G2/M phase, according to assessments of the cell cycle and apoptosis. Additionally, as compared to the control, compound **XIV** increased the level of caspase-3 in HepG-2 by 6.5 times^48^. In compounds **XV - XVII**, pyrimidinone-5-carbonitrile scaffold with a hydrazine, hydrazide, or hydrazone moiety conferred promise chemotherapeutic efficacy as anticancer drugs^49^^,^^50^. Compound **XVII** caused cell cycle arrest at the G2-M phase, a significant increase in the percentage of annexin V-FITC-positive apoptotic cells and increased the level of active caspase-3. It also exhibited remarkable broad spectrum antitumor activity with GI_50_ (median growth inhibition) and TGI (total growth inhibition) values of 6.15 and 28.66 µM, respectively. The safety profile of **XVII** against transformed human liver epithelial-2 (THLE2) was also good^51^. Compound **XVII '**s antiproliferative impact on the leukaemia SR cell line was linked to G2/M phase cell cycle disruption, pre-G1 death, and an increase in caspase-3 activity^51^ (Figure 2).

**Figure 3.**
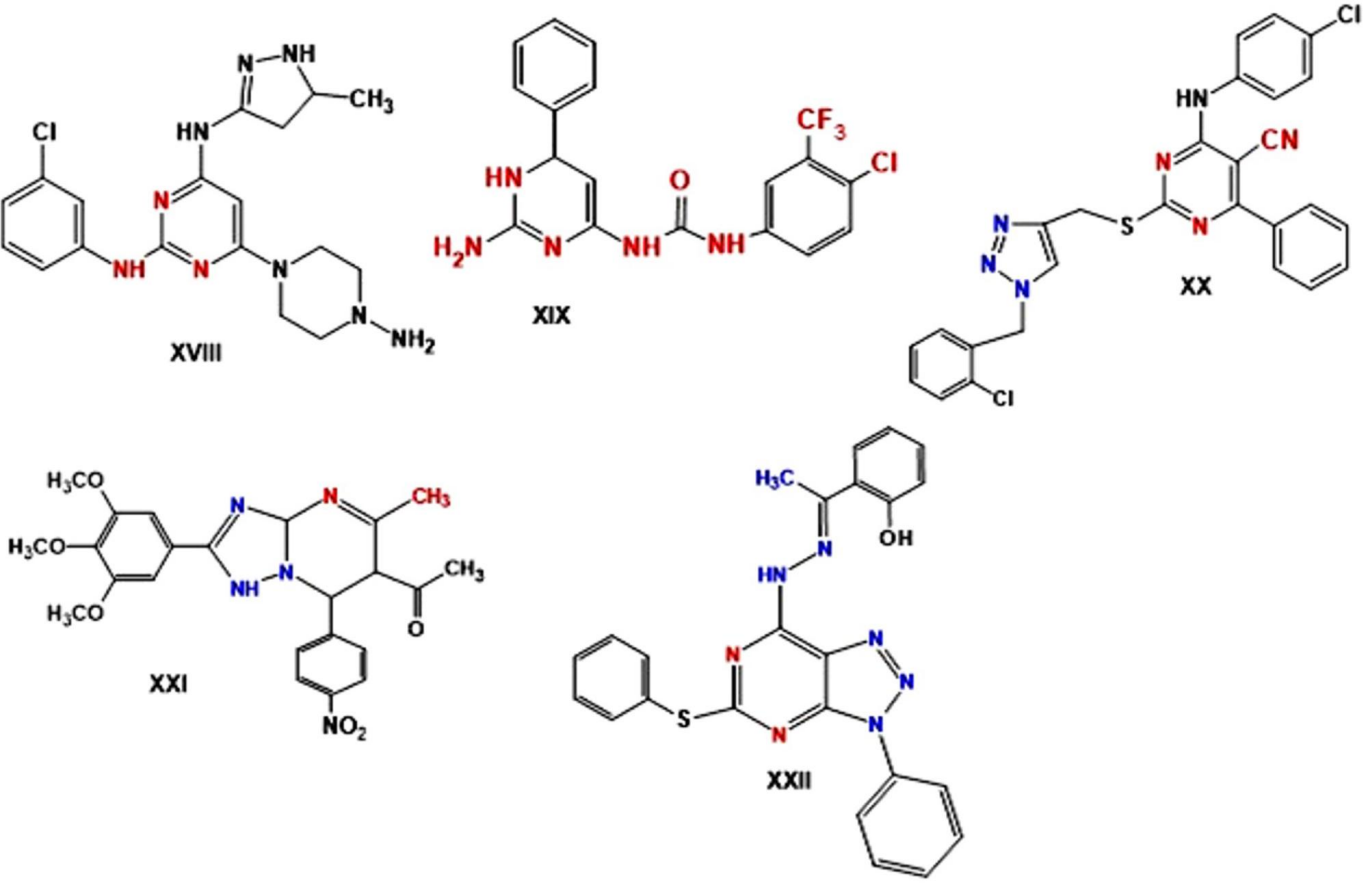
Reported pyrimidine derivatives **(XVIII–XXII)** with apoptotic activity.

Many substituted pyrimidine compounds with anticancer action have been found in recent years. The compound **XVIII** demonstrated strong growth inhibition in solid CNE-2 tumour cells and cell-cycle arrest at the G2/M phase^52^. The significant cytotoxic activity of 2-aminopyrimidine **XIX**, with an IC_50_ value of 11.08 µM was demonstrated against the cancer cell line SW480. Through the upregulation of Bax, Ikb-α and cleaved PARP and the downregulation of Bcl-2 expression levels, compound **XIX** arrested the cell cycle at the G2/M phase and triggered apoptosis, according to mechanistic studies. Additionally, compound **XIX** caused loss of the mitochondrial membrane potential in SW480 cells^53^. In addition, triazole-pyrimidines exerted promising anticancer activity. According to Ma et al., the anticancer efficacy of various 1,2,3-triazole-pyrimidine hybrids was tested against several cancer cell lines. With IC_50_ values against all cancer cell lines ranging from 1.42 to 6.52 µM, compound **XX** (Figure 3) was found to be the most active derivative of these. Apoptosis induction and cell cycle arrest in the G2/M phase were found to be the mechanisms by which compound **XX’s** cytotoxicity in EC-109 was mediated^54^. Recently, [1,2,4]triazolo[1,5-*a*]pyrimidine **XXI** effectively suppressed the growth of A549 and HeLa cell lines, with IC_50_ values of 1.02 and 0.75 μM, respectively. HeLa cells arrested in the G2/M phase of the cell cycle and negatively impacted the microtubule networks and cell morphology^55^^.^ Hydrazone bearing [1,2,3] triazolo[4,5-*d*] pyrimidine **XXII** showed anticancer efficiency against the MGC-803 cell line (IC_50_ = 0.87 μM) and the GES1(IC_50_ = 56.17 μM) and being selective towards normal cell line and the cancer cell line. The compound increases the expression of Bax and Bak, activates caspase-9/3 and p-53, and downregulates Mcl-1 and Bcl_2_ to decrease the mitochondrial pathway, which in turn induce apoptosis. Additionally, at 0.8 μM, the compound prevented the growth of MGC803 cells (Figure 3).^56^

PI3K inhibitors are a potential class of small molecule treatments that have been intensively researched in a variety of cancers, including B-cell malignancies, as monotherapy and in various combinations.

In patients with chronic lymphocytic leukaemia (CLL), there are two types of medicines that target the B cell receptor (BCR): Bruton’s tyrosine kinase (BTK) inhibitors (BTKis) and phosphoinositide 3-kinase inhibitors (PI3Kis)^57^^,^^58^.

Phosphatidylinositol-3 kinases (PI3Ks)^59^ are lipid kinases that phosphorylate PIP2 to PIP3, a signalling lipid. PIP3 molecules recruit proteins with PIP3-binding pleckstrin homology (PH) domains to the plasma membrane, such as Akt in the PI3K/Akt/mTOR signalling pathway. The PI3K pathway is one of the most frequently activated in human tumours, accounting for 50% of all cancers^60^. Activation of this system promotes cell survival, proliferation, and angiogenesis while inhibiting apoptosis via different pathways^61–64^^.^

Dysregulation of the PI3K pathway has also been reported in a variety of diseases such as diabetes, thrombosis, rheumatoid arthritis, asthma, and cancer^65^.

The PI3K kinases are classified into three classes, with class I being the most significant in lymphocyte biology and CLL pathogenesis.

The class I PI3Ks are responsible for the phosphorylation of phosphatidylinositol 4,5-bisphosphate (PIP2) to generate phosphatidylinositol (3,4,5)-trisphosphate (PIP3)^66^. PIP3 operates as a secondary messenger, acting as a scaffold for further downstream signalling in the BCR pathway. Among the class I PI3Ks, four isoforms may be distinguished: PI3Kα, PI3Kβ, PI3Kγ, and PI3Kδ^67^.

PI3Kα and PI3Kβ are ubiquitously expressed in many tissues, while PI3Kγ is expressed specifically in T lymphocytes^68^. PI3Kγ inhibition impairs T lymphocyte, neutrophil, and macrophage function but has no effect on B lymphocytes^69–71^. PI3Kδ expression is restricted mostly to B lymphocytes and their precursors, whereas PI3Kα and PI3Kγ expression levels are low, and PI3K γ expression is almost absent^72^. It has been demonstrated that PI3Kδ and PI3Kα are responsible for proper B lymphocyte development^67^^,^^72^^,^^73^.

Novel medicines have been developed to target certain isoforms of Class I PI3K (α, ß, γ and δ), which appears to be more important in oncology than PI3K classes II and III^74^^,^^75^^.^

**Figure 4.**
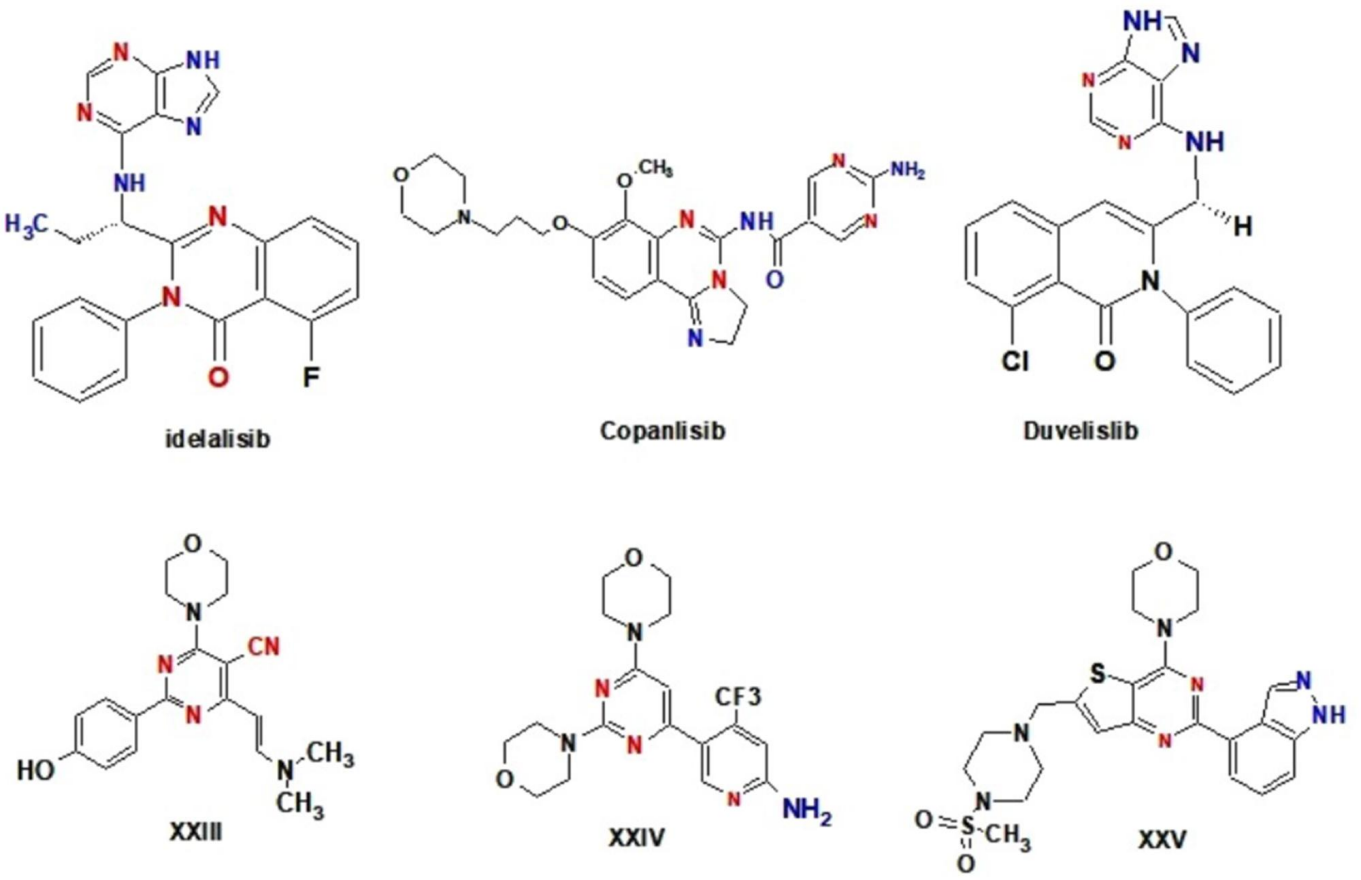
Structure of some reported PI3K inhibitors.

Idelalisib, copanlisib and duvelisib are FDA-approved PI3K inhibitors for the treatment of B-cell malignancies. Idelalisib is an oral, highly selective PI3Kδ inhibitor; copanlisib is an injectable pan-class I PI3K inhibitor with predominant activity against the PI3Kδ and PI3Kα isoforms; duvelisib (IPI-145) is a dual PI3Kδ and PI3Kγ inhibitor; and umbralisib is a dual PI3Kδ and CK1ε inhibitor (Figure 4).

Furthermore, a review of the literature found that a wide range of pyrimidine derivatives exhibited substantial antiproliferative activity due to their high PI3K inhibitory impact^76–80^. Morpholino pyrimidine (WJD008) **XXIII**, for example, was discovered to inhibit the kinase activity of PI3K p110 α and to have superior antiproliferative efficacy against a panel of cancer cells^81^. Furthermore, buparlisib (BKM-120) **XXIV**, is being tested in patients with advanced solid tumours^82^^,^^83^. Pictilisib (GDC-0941) **XXV**, an orally accessible thieno pyrimidine derivative, has also entered Phase I clinical studies in cancer patients^84^^,^^85^ (Figure 4).

**Figure 5.**
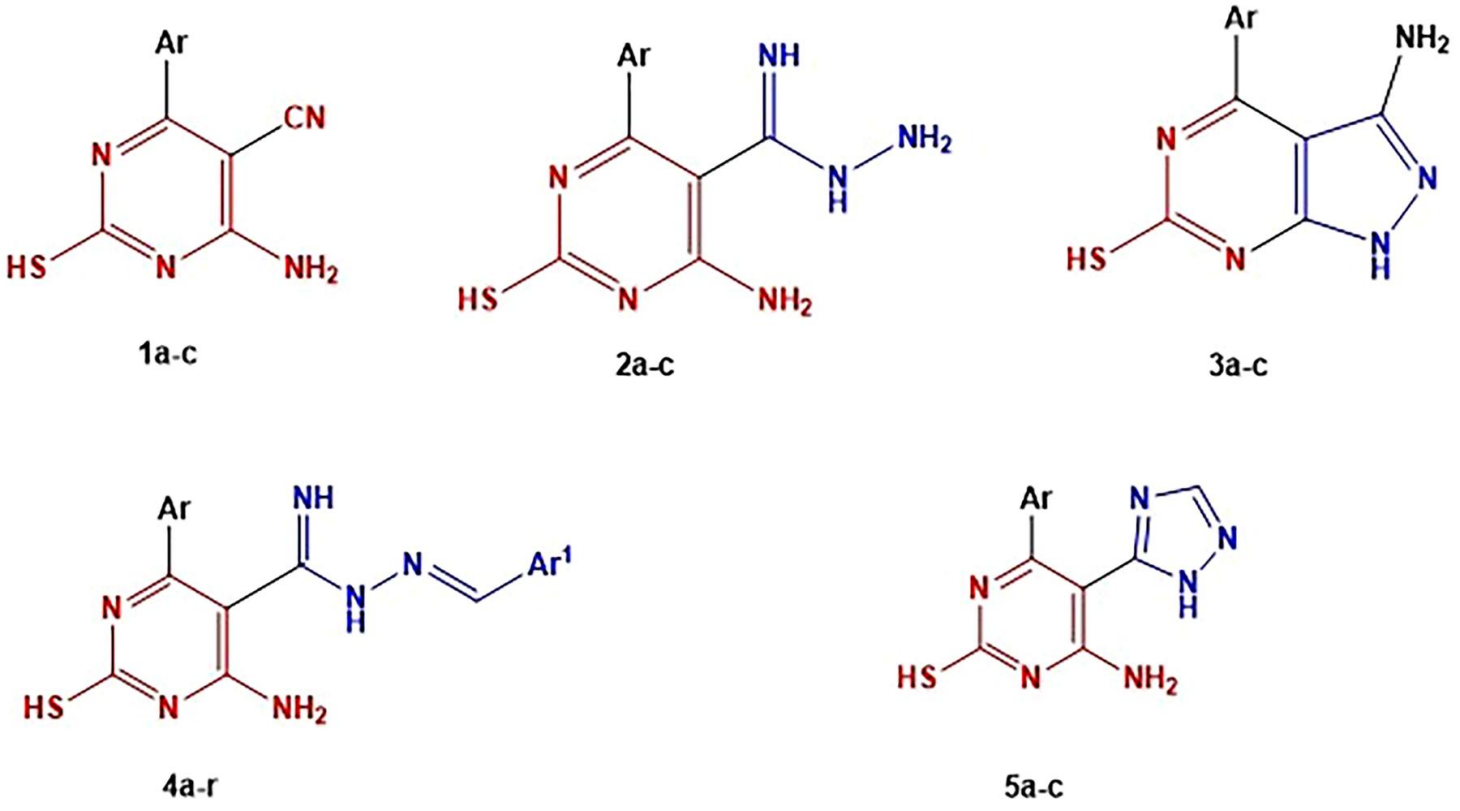
The designed compounds as anticancer, apoptotic inducing agents and PI3K inhibitors.

In light of the facts and as part of our continuous work to create brand-new, powerful anticancer drugs based on the pyrimidine moiety, two strategies were applied to develop the four series. First, the essential core of rational includes chemical modification at the 5-position of 5-cyanopyrimidine by replacing the 5-cyano group of compounds **1a-c** with (carboximidohydrazide group), aryl methylidene or triazole ring in compound **2a-c**, **4a-r** and **5a-c**, respectively. Second, cyclisation of the cyano and amino group of compounds **1a-c** to the corresponding pyrazolopyrimidine analogues **3a-c** (Figure 5).

Here, we present the design and synthesis of novel 6-amino-5-cyano-2-thiopyrimidine derivatives **1–5** and investigate the mechanism of thiopyrimidine-induced apoptosis, with the main objective of creating effective anticancer drugs. Then, we investigated the effect of thiopyrimidine on cell cycle arrest of HL-60 and leukaemia SR cells.

Our findings could pave the way for the use of thiopyrimidine as a potential therapeutic candidate for leukaemia.

## Materials and methods

### Instruments

All melting points are uncorrected and are measured using Electro-thermal 1 A 9100 apparatus (Shimadzu, Japan). IR spectra were recorded as potassium bromide (KBr) discs on a Perkin-Elmer1650 Spectrophotometer (USA), Faculty of Science, Cairo University, Cairo, Egypt.^1^HNMR spectra were recorded in DMSO-d6 on a Varian Mercury (300 MHz) spectrometer (Varian UK) using TMS as internal standard and chemical shifts were given as ppm (Faculty of Science, Cairo University, Cairo, Egypt). The mass spectra were carried out on El MS-1000 EX (Shimadzu, Japan) at electron energy 70 eV, Microanalytical Centre, Faculty of Science, Cairo University, Giza, Egypt. Microanalyses were performed on Vario, Elementar apparatus (Shimadzu, Japan), Organic Microanalysis Unit, Faculty of Science, Cairo University, Cairo, Egypt and the results were within the accepted range (0.40) of the calculated values. The progress of the reactions was monitored by TLC sheets precoated with UV fluorescent silica gel (Merck 60 F254) in chloroform/methanol (3:1) and spots were visualised using UV-light.

### Chemistry

#### 4-Amino-6-aryl-2-Sulfanylpyrimidine-5-carbonitrile (1a-c)

The titled compounds **1a-c** were synthesised according to the reported methods^86^.

A mixture of the appropriate aromatic aldehyde (0.01 mol), malononitrile (0.01 mol) and phosphorus pentoxide (0.0035 mol) was stirred mechanically for 10 m in absolute ethanol (25 ml) and then thiourea (0.02 mol) was added and mixed thoroughly. The resulting reaction mixture was refluxed at 70 °C for 5–8 h. The reaction mixture was allowed to cool and poured on crushed ice, the precipitate formed was filtered, dried, washed with petroleum ether and crystallised from ethanol to give compounds **1a-c.**

***4-Amino-6–(2,4-dichlorophenyl)-2-mercaptopyrimidine-5-carbonitrile (1c).*** Yellow crystal, yield 73%, m.p0.158–160 °C. IR (KBr) v max (cm^−1^): 3396 (NH_2_), 3042 (CH-Ar), 2224 (CN), 1577 (C = N). ^1^H NMR (300 MHz, DMSO-d_6_) *δ*:3.2 (s, 1H, SH, D_2_O exchangeable), 7.36 (d, 1H, J = 12 Hz, Ar-H), 7.50 (s, 1H, Ar-H), 8.03 (d, 1H, J = 10 Hz, Ar-H), 7.51 (s, 2H, NH_2_, D_2_O exchangeable). ^13^C NMR (300 MHz, DMSO-d_6_): *δ* 90.1 (C-5 pyrimidine), 115.5 (CN), 124.1–145.0 (aromatic Cs), 161.9 (C-4 pyrimidine), 166.6 (C-6 pyrimidine), 180.0 (C-2 pyrimidine). MS (EI): m/z: 297 [M^+^] (52.4%); 298 [M^+^ +2] (35%); 300 [M^+^ +4] (5.8%). Anal. Calcd for C_11_H_6_Cl_2_N_4_S (297.16): C, 44.46 H, 2.04; N, 18.85; Found: C, 44.46; H, 2.04; N, 18.85.

#### 4-Amino-6-aryl-2-Sulfanylpyrimidine-5-carboximidohydrazide (2a-c)

A solution of thiopyrimidine derivative (**1a-c**) (0.01 mol) and an excess of 99% hydrazine hydrate (30 ml) was stirred on cold, then the precipitate was poured onto ice, filtered, and dried to give compounds **2a-c.**

***4-Amino-6-[4-(dimethylamino)phenyl]-2-mercaptopyrimidine-5-carboximidohydrazide (2a).*** Lemon yellow powder, yield 88%, m.p. 263–265 °C. IR (KBr) v _max_ (cm^−1^): 3430 (NH_2_), 2909 (CH-sp3), 1599 (C = N).^1^HNMR (300 MHz,DMSO-d_6_) *δ*:2.4 (s, 1H, SH, D_2_O exchangeable), 2.9 (s, 6H, N-CH_3_), 6.74, 6.77 (dd, 2H, J = 16.20 Hz Ar-H), 7.62, 7.65 (dd, 2H, J = 10.20 Hz, Ar-H), 7.8, 8.1 (2s, 4H, 2(NH_2_), D_2_O exchangeable), 8.3, 8.5 (2s, 2H, 2NH, D_2_O exchangeable). ^13^C NMR (300 MHz, DMSO-d_6_): *δ* 41.3 (N-CH_3_), 107.0 (C-5 pyrimidine), 112.0–155.0 (aromatic Cs), 152.0 (C-6 pyrimidine), 158.0 (HN = C), 164.0 (C-4 pyrimidine), 180.0 (C-2 pyrimidine). MS (EI): m/z: 303 [M^+^] (3.6%). Anal. Calcd for C_13_H_17_N_7_S (303.38): C, 51.47; H, 5.65; N, 32.32; Found: C, 51.38; H, 5.58; N, 32.11.

***4-Amino-6–(4-chlorophenyl)-2-mercaptopyrimidine-5-carboximidohydrazide (2b).*** Yellow crystal, yield 71%, m.p. 213–215 °C.IR (KBr) v _max_ (cm^−1^): 3339 (NH_2_), 3088 (CH-Ar), 1598 (C = N). ^1^H NMR (300 MHz, DMSO-d_6_) *δ*:2.4 (s, 1H, SH, D_2_O exchangeable), 7.55, 7.58 (dd, 2H, J = 40.70 Hz, Ar-H), 7.87, 7.90 (dd, 2H, J = 38.0 Hz, Ar-H), 8.0, 8.2 (2s, 4H, 2(NH_2_), D_2_O exchangeable), 8.5, 8.6 (2s, 2H, 2NH, D_2_O exchangeable). ^13^C NMR (300 MHz, DMSO-d_6_): *δ* 107.84 (C-5 pyrimidine), 125.26–134.17 (aromatic Cs), 152.38 (C-6 pyrimidine) 157.57 (HN = C) 164.86 (C-4 pyrimidine), 180.38 (C-2 pyrimidine). MS (EI): m/z: 294 [M^+^] (5%); 296 [M^+^ +2] (1.7%). Anal. Calcd for C_11_H_1__1_N_6_SCl (294.76): C, 44.82; H, 3.76; N, 28.51; Found: C, 44.60; H, 3.74; N, 28.48.

***4-Amino-6–(2,4-dichlorophenyl)-2-mercaptopyrimidine-5-carboximidohydrazide (2c).*** Buff powder, yield 77%, m.p. 113–115 °C. IR (KBr) v_max_ (cm^−1^): 3371 (NH_2_), 3204 (CH-Ar), 1590 (C = N). ^1^H NMR (300 MHz,DMSO-d_6_) *δ*:2.51 (s, 1H, SH, D_2_O exchangeable), 7.3 (d, 1H, J = 16.43 Hz Ar-H), 7.5 (s, 1H, Ar-H), 7.7 (d, 1H, J = 16.01 Hz, Ar-H), 7.9, 8.12 (2s, 4H, 2(NH_2_), D_2_O exchangeable), 8.15, 8.8 (2s, 2H, 2NH, D_2_O exchangeable). ^13^C NMR (300 MHz, DMSO-d_6_): δ 107.5 (C-5 pyrimidine), 128.0–135.7 (aromatic Cs), 152.2(C-6 pyrimidine) 158.5 (HN = C) 164.6 (C-4 pyrimidine), 180.0 (C-2 pyrimidine). MS (EI): m/z: 328[M^+^] (8.92%); 330 [M^+^ +2] (5.9%); 332 [M^+^ +4] (1%). Anal. Calcd for C_11_H_10_Cl_2_N_6_S (329.20): C, 40.13; H, 3.06; N, 25.53; Found: C, 39.89; H, 3.03; N, 25.22.

#### 3-Amino-4-aryl-1H-pyrazolo[3,4-d] pyrimidine-6-thiol (3a-c)

A mixture of thiopyrimidines **1a-c** (0.003 mol) and hydrazine hydrate 99% (0.04 mol) in absolute ethanol (30 ml) was heated under reflux for 24h. The precipitate formed was poured onto ice, filtered, dried, and crystallised from ethanol to give compounds **3a-c**.

***3-Amino-4-[4-(dimethylamino)phenyl]-1H-pyrazolo[3,4-d]pyrimidine-6-thiol (3a).*** Orange powder, yield 80%, m.p. 248–250 °C. IR (KBr) v _max_ (cm^−1^): 3431 (broad NH, NH_2_), 2913 (CH-sp3), 1606(C = N). ^1^H NMR (300 MHz, DMSO-d_6_) *δ*:2.9 (s, 1H, SH, D_2_O exchangeable),3 (s, 6H, N-CH_3_), 6.73, 6.76 (dd, 2H, J = 17.24 Hz, Ar-H), 7.61, 7.64 (dd, 2H, J = 10.66 Hz, Ar-H), 8.47 (s, 2H, NH_2_, D_2_O exchangeable), 9.7 (s, 1H, NH, D_2_O exchangeable). ^13^C NMR (300 MHz, DMSO-d_6_): *δ* 40.22 (N-CH_3_), 91.78 (C-4 pyrazole), 111.28–159.65 (aromatic Cs), 152.27 (C-5 pyrazole), 158.51 (C-3 pyrazole), 164.922 (C-4 pyrimidine), 175.78 (C-2 pyrimidine). MS (EI): m/z: 286 [M^+^] (15.2%). Anal. Calcd for C_13_H_14_N_6_S (286.35): C, 54.53 H, 4.93; N, 29.35; Found: C, 54.73; H, 4.88; N, 29.21.

***3-Amino-4–(4-chlorophenyl)-1H-pyrazolo[3,4-d] pyrimidine-6-thiol (3b).*** White powder, yield 60%, m.p. 228–230 °C. IR (KBr) v _max_ (cm^−1^): 3434 (broad NH, NH_2_), 3046 (CH-Ar), 1623 (C = N). ^1^H NMR (300 MHz,DMSO-d_6_) *δ*:2.4 (s, 1H, SH, D_2_O exchangeable), 7.57, 7.59 (dd, 2H, J = 40.78 Hz, Ar-H), 7.88, 7.91(dd, 2H, J = 38.36 Hz, Ar-H), 8.2(s, 2H, NH_2_, D_2_O exchangeab-le), 8.7 (s, 1H, NH, D_2_O exchangeable). ^13^C NMR (300 MHz, DMSO-d_6_): *δ* 91.5 (C-4 pyrazole), 127.0–135.0 (aromatic Cs), 150.5 (C-5 pyrazole), 151.5 (C-3 pyrazole), 163.3 (C-4 pyrimidine), 180.0 (C-2 pyrimidine). MS (EI): m/z: 277 [M^+^] (20.8%); 279 [M^+^ +2] (6.9%). Anal. Calcd for C_11_H_8_ClN_5_S (277.73): C, 47.57 H, 2.90; N, 25.22; Found: C, 47.26; H, 2.84; N, 25.42.

***3-Amino-4–(2,4-dichlorophenyl)-1H-pyrazolo[3,4-d] pyrimidine-6-thiol (3c).*** White powder, yield 60%, m.p. 220–222 °C. IR (KBr) v_max_ (cm^−1^): 3403 (broad NH, NH_2_), 3086 (CH-Ar), 1614 (C = N). ^1^H NMR (300 MHz, DMSO-d_6_) *δ*:2.9 (s, 1H, SH, D_2_O exchangeable), 7.5 (d, 1H, J = 15.01 Hz, Ar-H) 7.8 (s, 1H, J = 13.1 Hz, Ar-H), 8.2 (d, 1H, J = 13.01 Hz, Ar-H), 8.4 (s, 2H, NH_2_, D_2_O exchangeable), 8.9 (s, 2H, NH, D_2_O exchangeable). ^13^C NMR (300 MHz, DMSO-d_6_): *δ* 91.1 (C-4 pyrazole), 127.5–135.5 (aromatic Cs), 150.1 (C-5 pyrazole), 151.3 (C-3 pyrazole), 163.0 (C-4 pyrimidine), 180.1 (C-2 pyrimidine). MS (EI): m/z: 311 [M^+^] (54.3%); 313 [M^+^ +2] (36.2%); 315 [M^+^ +4] (6%). Anal. Calcd for C_1__1_H_7_Cl_2_N_5_S (312.17): C, 42.32; H, 2.26; N, 22.43; Found: C, 42.25; H, 2.30; N, 22.69.

#### 4-Amino-6-aryl-N'-[(E)-arylmethylidene]-2-sulfanylpyrimidine-5-carboximido hydrazide (4a-r)

Thiopyrimidine derivative **3a-c** (0.003 mol) was dissolved in absolute ethanol (20 ml), and the corresponding aromatic aldehyde (0.003 mol) was added. The reaction mixture was refluxed for 24h. The precipitate formed was poured onto ice, filtered, dried, and crystallised from ethanol to give compounds **4a-r.**

#### 4-Amino-6-[4-(dimethylamino)phenyl]-2-mercapto-N'-[phenylmethylene] pyrimidine-5-carboximidohydrazide (4a)

Pale green powder, yield 55%, m.p. 220–222 °C. IR (KBr) v_max_ (cm^−1^): 3320–3436 (NH, NH_2_), 2913 (CH-sp3),1605 (C = N). ^1^H NMR (300 MHz, DMSO-d_6_) *δ*:2.7 (s, 1H, SH, D_2_O exchangeable), 2.9 (s, 6H, N-CH_3_), 6.6–7.6 (m, 9H, Ar-H), 7.8 (s, 2H, NH_2_, D_2_O exchangeable), 8.4 (s, 1H, -N = CH), 8.1, 8.6 (2s, 2H, 2NH, D_2_O exchangeable). ^13^C NMR (300 MHz, DMSO-d_6_): *δ* 41.3 (N-CH_3_), 107.9 (C-5 pyrimidine), 112.7–155.0 (aromatic Cs), 146.8 (CH = N), 152.1 (C-6 pyrimidine) 163.0 (HN = C) 164.7 (C-4 pyrimidine), 181.0 (C-2 pyrimidine). MS (EI): m/z: 391 [M^+^] 2.8 (%). Anal. Calcd for C_20_H_21_N_7_S (391.49): C, 61.36; H, 5.41; N, 25.04; Found: C, 61.12; H, 5.48; N, 24.39.

#### 4-amino-N'-[(4-chlorophenyl)methylene]-6-[4-(dimethylamino)phenyl]-2-mercapto pyrimidine-5-carboximidohydrazide (4b)

Buff powder, yield 60%, m.p. 215–217 °C. IR (KBr) v_max_ (cm^−1^): 3341–3453 (NH, NH_2_), 3015 (CH-Ar), 1602 (C = N). ^1^H NMR (300 MHz, DMSO-d_6_) *δ*:2.9 (s, 1H, SH, D_2_O exchangeable), 3.1 (s, 6H, N-CH_3_), 6.7–7.8 (m, 8H, Ar-H), 8.1 (s, 2H, NH_2_, D_2_O exchangeable), 8.7 (s, 1H, -N = CH), 8.3, 9.0 (2s, 2H, 2NH, D_2_O exchangeable). ^13^C NMR (300 MHz, DMSO-d_6_): *δ* 107.3 (C-5 pyrimidine), 113.8–155.3 (aromatic Cs), 146.4 (CH = N), 152.5 (C-6 pyrimidine) 163.1 (HN = C) 164.4 (C-4 pyrimidine), 181.0 (C-2 pyrimidine). MS (EI): m/z: 425 [M^+^] (9.3%); 427 [M^+^ +2] (3.1%). Anal. Calcd for C_20_H_20_ClN_7_S (425.93): C, 56.40; H, 4.73; N, 23.02; Found: C, 56.74; H, 4.69; N, 22.83.

**4*-amino-N'-[6-[4-(dimethylamino)phenyl]-(4-nitrophenyl) methylene]-2- mercapto pyrimidine-5-carboximidohydrazide (4c).*** Brown powder, yield 62%, m.p. 228–230 °C. IR (KBr) v _max_ (cm^−1^): 3385–3500 (NH, NH_2_), 3012 (CH-Ar), 1603 (C = N), 1550 (N = O), 1383 (N-O). ^1^H NMR (300 MHz, DMSO-d_6_) *δ*:2.8 (s, 1H, SH, D_2_O exchangeable), 3.0 (s, 6H, N-CH_3_), 6.7–7.8 (m, 8H, Ar-Hs), 7.9 (s, 2H, NH_2_, D_2_O exchangeable), 8.6 (s, 1H, -N = CH), 8.2, 8.9 (s, 2H, 2NH, D_2_O exchangeable). ^13^C NMR (300 MHz, DMSO-d_6_): *δ* 107.2 (C-5 pyrimidine), 113.5–155.6 (aromatic Cs), 146.8 (CH = N), 152.2 (C-6 pyrimidine) 163.3 (HN = C) 164.6 (C-4 pyrimidine), 181.0 (C-2 pyrimidine). MS (EI): m/z: 436 [M^+^] (8.18%). Anal. Calcd for C_20_H_20_N_8_O_2_S (436.49): C, 55.03; H, 4.62; N, 25.67; Found: C, 54.92; H, 4.55; N, 25.46.

***4-amino-N'-[(1Z)-(4-methoxyphenyl)methylene]-6-[4-(dimethylamino)phenyl]-2-mercapto pyrimidine-5-carboximidohydrazide (4d).*** Pale green powder, yield 55%, m.p. 233–235 °C. IR (KBr)v _max_ (cm^−1^): 3353–3410 (NH, NH_2_), 2950 (CH-sp3), 1605 (C = N). ^1^H NMR (300 MHz, DMSO-d_6_) *δ*:2.6 (s, 1H, SH, D_2_O exchangeable), 2.8 (s, 6H, N-CH_3_), 3.6 (s, 3H, OCH_3_), 6.9–7.7 (m, 8H, Ar-H), 8.0 (s, 2H, NH_2_, D_2_O exchangeable), 8.8 (s, 1H, -N = CH), 8.3, 9.1 (s, 2H, 2NH, D_2_O exchangeable). ^13^C NMR (300 MHz, DMSO-d_6_): *δ* 55.8(OCH_3_), 107.1 (C-5 pyrimidine), 113.6–162.0 (aromatic Cs), 146.3 (CH = N), 152.3 (C-6 pyrimidine) 163.6 (HN = C) 164.8 (C-4 pyrimidine), 181.0 (C-2 pyrimidine). MS (EI): m/z: 421 [M^+^] (1.2%). Anal. Calcd for C_21_H_23_N_7_OS (421.51): C, 59.84; H, 5.50; N, 23.26; Found: C, 59.71; H, 5.56; N, 23.58.

***4-amino-N'-[(4-bromophenyl)methylene]-6-[4-(dimethylamino)phenyl]-2-mercapto pyrimidine-5-carboximidohydrazide (4e).*** Buff powder, yield 75%, m.p. 230–232 °C. IR (KBr) v_max_ (cm^−1^): 3382– 3426 (NH, NH_2_), 3015 (CH-Ar), 1606 (C = N). ^1^H NMR (300 MHz, DMSO-d_6_) *δ*:2.7 (s, 1H, SH, D_2_O exchangeable), 2.9 (s, 6H, N-CH_3_), 6.5–7.8 (m, 8H, Ar-H), 8.2 (s, 2H, NH_2_, D_2_O exchangeable), 8.6 (s, 1H, -N = CH), 8.4, 8.8 (s, 2H, 2NH, D_2_O exchangeable). ^13^C NMR (300 MHz, DMSO-d_6_): *δ* 108.2 (C-5 pyrimidine), 112.7–155.3 (aromatic Cs), 144.1 (CH = N), 153.1 (C-6 pyrimidine) 163.5 (HN = C) 165.8 (C-4 pyrimidine), 181.7 (C-2 pyrimidine). MS (EI): m/z: 469 [M^+^] (3%); 471 [M^+^ +2](3%). Anal. Calcd for C_20_H_20_BrN_7_S (470.38): C, 51.07; H, 4.29; N, 20.84; Found: C, 50.89; H, 4.25; N, 20.62.

***4-amino-N'-[(2,4-dichlorophenyl)methylene]-6-[4-(dimethylamino)phenyl]-2-mercapto pyrimidine-5-carboximidohydrazide (4f).*** Yellowish green powder, yield 71%, m.p. 228–230 °C. IR (KBr) v _max_ (cm^−1^): 3324–3480 (NH, NH_2_), 3010 (CH-Ar), 1610 (C = N). ^1^H NMR (300 MHz, DMSO-d_6_) *δ*:2.4 (s, 1H, SH, D_2_O exchangeable), 2.7 (s, 6H, N-CH_3_), 6.6–7.8 (m, 7H, Ar-Hs), 7.9 (s,1H, N = CH), 8.3 (s, 2H, NH_2_, D_2_O exchangeable), 8.5, 8.7 (s, 2H, 2NH, D_2_O exchangeable). ^13^C NMR (300 MHz, DMSO-d_6_): *δ* 107.5 (C-5 pyrimidine), 114.3–155.7 (aromatic Cs), 146.6 (CH = N), 152.5 (C-6 pyrimidine), 163.6 (HN = C), 164.6 (C-4 pyrimidine), 181.0 (C-2 pyrimidine). MS (EI): m/z: 459 [M^+^] (12.4%); 461 [M^+^ +2] (8.2%); 463 [M^+^ +4] (1.4%). Anal. Calcd for C_20_H_19_Cl_2_N_7_S (460.38): C, 52.18; H, 4.16; N, 21.30; Found: C, 51.80; H, 4.20; N, 21.64.

***4-amino-6–(4-chlorophenyl)-2-mercapto-N'-[phenylmethylene]pyrimidine-5-carboximido hydrazide (4g).*** Pale green powder, yield 63%, m.p. 250–252 °C. IR (KBr) v_max_ (cm^−1^): 3348–3450 (NH, NH_2_), 3020 (CH-Ar), 1600 (C = N). ^1^H NMR (300 MHz, DMSO-d_6_) *δ*:2.8 (s, 1H, SH, D_2_O exchangeable), 6.5–7.8 (m, 9H, Ar-H), 8.1 (s, 1H, N = CH), 8.4 (s, 2H, NH_2_, D_2_O exchangeable), 8.6, 8.8 (s, 2H, 2NH, D_2_O exchangeable). ^13^C NMR (300 MHz, DMSO-d_6_): *δ* 108.0 (C-5 pyrimidine), 128.8–135.0 (aromatic Cs), 147.1 (CH = N), 153.1 (C-6 pyrimidine) 164.1 (HN = C) 165.1 (C-4 pyrimidine), 181.0 (C-2 pyrimidine). MS (EI): m/z: 382 [M^+^] (6.3%); 384 [M^+^ +2] (3.1%). Anal. Calcd for C_18_H_15_ClN_6_S (382.86): C, 56.47; H, 3.95; N, 21.95; Found: C, 56.71; H, 3.88; N, 22.11.

***4-amino-6–(4-chlorophenyl)-N'-[(4-chlorophenyl)methylene]-2-mercapto pyrimidine-5-carboximidohydrazide (4h).*** Yellowish green powder, yield 62%, m.p. 200–205 °C. IR (KBr) v_max_ (cm^−1^): 3308–3465 (NH, NH_2_), 3025 (CH-Ar), 1615 (C = N). ^1^H NMR (300 MHz, DMSO-d_6_) *δ*:3.0 (s, 1H, SH, D_2_O exchangeable), 6.7–7.8 (m, 8H, Ar-Hs), 7.9 (s, 2H, NH_2_, D_2_O exchangeable), 8.4 (s, 1H, -N = CH), 8.1, 9.0 (2s, 2H, 2NH, D_2_O exchangeable). ^13^C NMR (300 MHz, DMSO-d_6_): *δ* 108.0 (C-5 pyrimidine), 129.0–136.6 (aromatic Cs), 147.2 (CH = N), 153.2 (C-6 pyrimidine) 164.2 (HN = C) 165.3 (C-4 pyrimidine), 181.1 (C-2 pyrimidine). MS (EI): m/z: 416 [M^+^] (3.6%);418 [M^+^+2] (2.4%); 420 [M^+^ +4] (0.4%). Anal. Calcd for C_18_H_14_Cl_2_N_6_S (417.31): C, 51.81; H, 3.38; N, 20.14; Found: C, 51.54; H, 3.35; N, 19.98.

***4-amino-6–(4-chlorophenyl)-2-mercapto-N'-[(4-nitrophenyl)methylene] pyrimidine-5-carboximidohydrazide (4i).*** Buff powder, yield 75%, m.p. 245–247 °C. IR (KBr) v _max_ (cm^−1^): (CH-Ar), 3393–3525 (NH, NH_2_), 3028 (CH-Ar), 1613 (C = N), 1552 (N = O), 1382 (N-O). ^1^H NMR (300 MHz,DMSO-d_6_) *δ*:2.7 (s, 1H, SH, D_2_O exchangeable), 6.5–7.7 (m, 8H, Ar-H), 8.0 (s, 2H, NH_2_, D_2_O exchangeable),8.5 (s, 1H, -N = CH), 8.3, 8.8 (s, 2H, 2NH, D_2_O exchangeable). ^13^C NMR (300 MHz, DMSO-d_6_): *δ* 108.1 (C-5 pyrimidine), 124.0–150.0 (aromatic Cs), 147.4 (CH = N), 153.3 (C-6 pyrimidine), 164.5 (HN = C), 165.0 (C-4 pyrimidine), 181.1 (C-2 pyrimidine). MS (EI): m/z: 427 [M^+^] (11.7%); 429 [M^+^ +2] (3.9%). Anal. Calcd for C_18_H_14_ClN_7_O_2_S (427.86): C, 50.53; H, 3.30; N, 22.92; Found: C, 20.23; H, 3.28; N, 22.71.

***4-amino-6–(4-chlorophenyl)-2-mercapto-N'-[(4-methoxyphenyl)methylene] pyrimidine- 5-carboximidohydrazide*** ***(4j).*** White powder, yield 72%, m.p. 290–292 °C. IR (KBr) v _max_ (cm^−1^): (CH-Ar), 3318–3512 (NH, NH_2_), 3060 (CH-Ar), 1613 (C = N). ^1^H NMR (300 MHz, DMSO-d_6_) *δ*:2.9 (s, 1H, SH, D_2_O exchangeable), 3.7 (s, 3H, OCH_3_), 6.5–7.6 (m, 8H, Ar-H), 7.8 (s, 2H, NH_2_, D_2_O exchangeable), 8.4 (s, 1H, -N = CH), 8.1, 8.8 (2s, 2H, 2NH, D_2_O exchangeable). ^13^C NMR (300 MHz, DMSO-d_6_): *δ* 55.1 (OCH_3_), 108.1 (C-5 pyrimidine),116.0–160.0 (aromatic Cs),147.1 (CH = N),153.4 (C-6 pyrimidine), 164.1 (HN = C), 165.0 (C-4 pyrimidine), 180.1 (C-2 pyrimidine). MS (EI): m/z: 412 [M^+^] (18.4%); 414 [M^+^ +2] (6.1%). Anal. Calcd for C_19_H_17_ClN_6_ OS (412.89): C, 55.27; H, 4.15; N, 20.35; Found: C, 54.96; H, 4.12; N, 20.04.

***4-amino-N'-[(4-bromophenyl)methylene]-6–(4-chlorophenyl)-2-mercapto pyrimidine-5-carboximidohydrazide (4k).*** Buff powder, yield 63%, m.p. 253–255 °C. IR (KBr) v_max_ (cm^−1^): 3386– 3495 (NH, NH_2_), 3030 (CH-Ar), 1614 (C = N). ^1^H NMR (300 MHz, DMSO-d_6_) *δ*:2.6 (s, 1H, SH, D_2_O exchangeable), 6.7–7.8 (m, 8H, Ar-H), 8.1 (s, 2H, NH_2_, D_2_O exchangeable), 8.6 (s, 1H, -N = CH), 8.3, 9.1 (s, 2H, 2NH, D_2_O exchangeable). ^13^C NMR (300 MHz, DMSO-d_6_): *δ* 55.1 (OCH_3_), 108.3 (C-5 pyrimidine), 125.0–134.0 (aromatic Cs), 147.2 (CH = N), 154.1 (C-6 pyrimidine), 164.2 (HN = C), 165.0 (C-4 pyrimidine), 180.10 (C-2 pyrimidine). MS (EI): m/z: 460 [M^+^] (25.7%); 462 [M^+^ +2](24.7%). Anal. Calcd for C_18_H_14_BrClN_6_S (461.76): C, 46.82; H, 3.06; N, 18.20; Found: C, 46.55; H, 3.09; N, 18.53.

***4-amino-6–(4-chlorophenyl)-N'-[(2,4-dichlorophenyl)methylene]-2-mercapto pyrimidine-5-carboximidohydrazide (4l).*** Buff powder, yield 70%, m.p. 240–242 °C.IR (KBr) v _max_ (cm^−1^): (CH-Ar), 3315–3474 (NH, NH_2_), 3080 (CH-Ar), 1618 (C = N). ^1^H NMR (300 MHz, DMSO-d_6_) *δ*:2.8 (s, 1H, SH, D_2_O exchangeable), 6.5–7.5 (m, 7H, Ar-H), 7.6 (s, 2H, NH_2_, D_2_O exchangeable), 8.5 (s, 1H, -N = CH), 8.2, 8.8 (s, 2H, 2NH, D_2_O exchangeable). ^13^C NMR (300 MHz, DMSO-d_6_): *δ* 55.1 (OCH_3_), 108.1 (C-5 pyrimidine), 129.0–138.0 (aromatic Cs), 147.3 (CH = N), 154.2 (C-6 pyrimidine), 164.2 (HN = C), 165.3 (C-4 pyrimidine),180.4 (C-2 pyrimidine). MS (EI): m/z: 450 [M^+^] (5%); 452 [M^+^ +2] (3.3%); 454 [M^+^ +4] (0.6%). Anal. Calcd for C_18_H_13_Cl_3_N_6_S (451.76): C, 47.86; H, 2.90; N, 18.60; Found: C, 48.02; H, 2.87; N, 18.45.

***4-Amino-6–(2,4-dichlorophenyl)-2-mercapto-N'-[phenylmethylene]pyrimidine-5-carboximidohydrazide (4m).*** Buff powder, yield 66%, m.p. 198–200 °C. IR (KBr) v_max_ (cm^−1^): 3332–3432 (NH, NH_2_), 3200 (CH-Ar), 1600 (C = N). ^1^H NMR (300 MHz, DMSO-d_6_) *δ*:2.4 (s, 1H, SH, D_2_O exchangeable), 7–7.7 (m, 8H, Ar-H), 7.9 (s, 2H, NH_2_, D_2_O exchangeable),8.4 (s, 1H, -N = CH), 8.1, 8.7 (s, 2H, 2NH, D_2_O exchangeable). ^13^C NMR (300 MHz, DMSO-d_6_): *δ* 109.0 (C-5 pyrimidine), 125.5–135.7 (aromatic Cs), 148.0 (CH = N), 154.0 (C-6 pyrimidine), 163.0 (HN = C), 164.0 (C-4 pyrimidine), 181.0 (C-2 pyrimidine). MS (EI): m/z: 416 [M^+^] (4.8%); 418 [M^+^ +2] (3.2%); 420 [M^+^ +4] (0.56%). Anal. Calcd for C_18_H_14_Cl_2_N_6_ S (417.31): C, 51.81; H, 3.38; N, 20.14; Found: C, 51.49; H, 3.35; N, 20.42.

***4-Amino-N'-[(4-chlorophenyl)methylene]-6–(2,4-dichlorophenyl)-2-mercapto pyrimidine-5-carboximidohydrazide (4n).*** Buff powder, yield 68%, m.p. 183–185 °C.IR (KBr) v _max_ (cm^−1^): 3356–3492 (NH, NH_2_), 3210 (CH-Ar), 1620 (C = N). ^1^H NMR (300 MHz, DMSO-d_6_) *δ*:2.6 (s, 1H, SH, D_2_O exchangeable), 6.8–7.6 (m, 7H, Ar-H), 8.0 (s, 2H, NH_2_, D_2_O exchangeable), 8.6 (s, 1H, -N = CH), 8.2, 8.9 (s, 2H, 2NH, D_2_O exchangeable). ^13^C NMR (300 MHz, DMSO-d_6_): *δ* 109.1 (C-5 pyrimidine), 127.6–136.6 (aromatic Cs), 148.1 (CH = N), 154.1 (C-6 pyrimidine), 163.3 (HN = C), 164.2 (C-4 pyrimidine), 181.5 (C-2 pyrimidine). MS (EI): m/z: 450 [M^+^] (57.5%); 452 [M^+^ +2] (38.3%); 454 [M^+^ +4] (6.3%). Anal. Calcd for C_18_H_13_Cl_3_N_6_S(451.76): C, 47.86; H, 2.90; N, 18.60; Found: C, 48.04; H, 2.94; N, 18.89.

***4-amino-6–(2,4-dichlorophenyl)-2-mercapto-N'-[(4-nitrophenyl)methylene] pyrimidine-5-carboximidohydrazide (4o).*** Yellow powder, yield 64%, m.p. 230–232 °C. IR (KBr) v_max_ (cm^−1^): 3300–3500 (NH, NH_2_), 3300 (CH-Ar), 1625 (C = N), 1500 (N = O), 1300 (N-O). ^1^H NMR (300 MHz, DMSO-d_6_) *δ*:2.8 (s, 1H, SH, D_2_O exchangeable), 6.6–7.5 (m, 7H, Ar-H), 7.7 (s, 2H, NH_2_, D_2_O exchangeable), 8.3 (s,1H, -N = CH), 8.0, 8.6 (s, 2H, 2NH, D_2_O exchangeable). ^13^C NMR (300 MHz, DMSO-d_6_): *δ* 109.2 (C-5 pyrimidine), 125.0–150.0 (aromatic Cs), 148.5 (CH = N), 154.2(C-6 pyrimidine), 163.6(HN =  C), 164.2(C-4 pyrimidine), 181.3(C-2 pyrimidine). MS (EI): m/z: 461 [M^+^] (6.4%); 463 [M^+^ +2](4.2%); 465 [M^+^ +4] (0.7%). Anal. Calcd for C_18_H_13_Cl_2_N_7_O_2_S (462.31): C, 46.76; H, 2.83; N, 21.21; Found: C, 46.48; H, 2.87; N, 20.98.

***4-amino-6–(2,4-dichlorophenyl)-2-mercapto-N'-[(4-methoxyphenyl)methylene] pyrimidine-5-carboximidohydrazide (4p).*** White powder, yield 66%, m.p. 192–195 °C.IR (KBr) v_max_ (cm^−1^): 3310–3520 (NH, NH_2_), 3350 (CH-Ar), 1630 (C = N). ^1^H NMR (300 MHz, DMSO-d_6_) *δ*:3.1 (s, 1H, SH, D2O exchangeable), 3.8 (s, 3H, OCH_3_), 7–7.8 (m, 7H, Ar-H), 7.5 (s, 2H, NH_2_, D_2_O exchangeable), 8.6 (s,1H, -N = CH), 8.1, 8.8 (s, 2H, 2NH, D_2_O exchangeable). ^13^C NMR (300 MHz, DMSO-d_6_): *δ* 55.6 (OCH_3_), 109.5 (C-5 pyrimidine), 115.0–160.0 (aromatic Cs), 148.4 (CH = N),154.3 (C-6 pyrimidine), 163.5 (HN = C), 164.3 (C-4 pyrimidine), 181.2 (C-2 pyrimidine). MS (EI): m/z: 446 [M^+^] (4.2%); 448 [M^+^ +2] (2.8%);450 [M^+^ +4] (0.5%). Anal. Calcd for C_19_H_16_Cl_2_N_6_OS (447.34): C, 51.01; H, 3.61; N, 18.79; Found: C, 50.84; H, 3.59; N, 18.47.

#### 4-amino-N'-[(4-bromophenyl)methylene]-6–(2,4-dichlorophenyl)-2-mercapto pyrimidine-5-carboximidohydrazide (4q)

White powder, yield 60%, m.p. 205–207 °C. IR (KBr) v _max_ (cm^−1^): 3380–3437 (NH, NH_2_), 3360 (CH-Ar), 1615 (C = N). ^1^H NMR (300 MHz, DMSO-d_6_) *δ*:2.4 (s, 1H, SH, D_2_O exchangeable), 6.8–7.6 (m, 7H, Ar-Hs), 8 (s, 2H, NH_2_, D_2_O exchangeable), 8.5 (s, 1H, -N = CH), 8.2, 8.9 (s, 2H, 2NH, D_2_O exchangeable). ^13^C NMR (300 MHz, DMSO-d_6_): *δ* 55.6 (OCH_3_), 109.5 (C-5 pyrimidine), 126.0–137.0 (aromatic Cs), 148.2 (CH = N), 154.2 (C-6 pyrimidine), 163.4 (HN = C), 164.8 (C-4 pyrimidine), 181.1 (C-2 pyrimidine). MS (EI): m/z: 494 [M^+^] (8.2%); 496 [M^+^ +2] (8.2%). Anal. Calcd for C_18_H_13_BrCl_2_N_6_S (496.21): C, 43.57; H, 2.64; N, 16.94; Found: C, 43.26; H, 2.70; N, 17.13.

***4-amino-6–(2,4-dichlorophenyl)-N'-[(2,4-dichlorophenyl)methylene]-2-mercapto pyrimidine-5-carboximidohydrazide (4r).*** Buff powder, yield 90%, m.p. 200–202 °C. IR (KBr) v_max_ (cm^−1^): 3330–3436 (NH, NH_2_), 3370 (CH-Ar), 1612 (C = N). ^1^H NMR (300 MHz, DMSO-d_6_) *δ*:2.8 (s, 1H, SH, D_2_O exchangeable), 6.6–7.5 (m, 6H, Ar-H), 7.7 (s, 2H, NH_2_, D_2_O exchangeable), 8.3 (s, 1H, -N = CH), 8.1, 8.7 (s, 2H, 2NH, D_2_O exchangeable). ^13^C NMR (300 MHz, DMSO-d_6_): *δ* 55.6 (OCH_3_), 109.5 (C-5 pyrimidine), 127.0–133.5 (aromatic Cs), 148.2 (CH = N), 154.2 (C-6 pyrimidine), 163.4 (HN = C), 164.8 (C-4 pyrimidine), 181.1 (C-2 pyrimidine). MS (EI): m/z: 484 [M^+^] (15.3%);486 [M^+^ +2] (10.2%); 488 [M^+^ +4] (1.7%). Anal. Calcd for C_18_H_12_Cl_4_N_6_S (486.20): C, 44.47; H, 2.49; N, 17.28; Found: C, 44.71; H, 2.46; N, 17.52.

#### 4-Amino-6-aryl-5-(1H-1,2,4-triazol-5-yl) pyrimidine-2-thiol (5a-c)

Thiopyrimidine derivative **3a-c** (0.003 mol) was refluxed in formic acid (30 ml) for 48h. the reaction mixture was allowed to cool, poured onto ice, and neutralised with NaHCO_3_. The precipitate formed was filtered, dried, and crystallised from ethanol to give compounds **5a-c.**

#### 4-Aamino-6-[4-(dimethylamino)phenyl]-5-(1H-1,2,4-triazol-5-yl)pyrimidine-2-thiol

**(5a).** Brown powder, yield 66%, m.p. 235 °C. IR (KBr) v_max_ (cm^−1^): 3300–3486 (NH, NH_2_) 3050 (CH-Ar), 2950 (CH-sp3),1605 (C = N). ^1^H NMR (300 MHz, DMSO-d_6_) *δ*:2.9 (s,1H, SH, D_2_Oexchangeable), 3.03 (s, 6H, N-CH_3_), 6.7–7.7 (m, 5H, Ar-Hs), 8.4 (s, 2H, NH_2_, D_2_O exchangea- ble), 9.6 (s, 1H, NH, D_2_O exchangeable). ^13^C NMR (300 MHz, DMSO-d_6_): *δ* 40.52 (N-CH_3_), 114.05 (C-5 pyrimidine), 104.75–152.009 (aromatic Cs), 151.55 (C-3 triazole), 157.98 (C-6 pyrimidine), 159.82 (C-5 triazole), 165.79 (C-4 pyrimidine), 178.04 (C-2 pyrimidine). MS (EI): m/z: 313 [M^+^] (1.2%). Anal. Calcd for C_14_H_15_N_7_S (313.38): C, 53.66; H, 4.82; N, 31.29; Found: C, 53.37; H, 4.78; N, 31.43.

***4-Amino-6–(4-chlorophenyl)-5-(1H-1,2,4-triazol-5-yl)pyrimidine-2-thiol (5b).*** Brown powder, yield 70%, m.p. >300 °C. IR (KBr) v_max_ (cm^−1^): 3365–3540 (NH, NH_2_), 3060 (CH-Ar), 1608 (C = N). ^1^H NMR (300 MHz, DMSO-d_6_) *δ*:2.7 (s, 1H, SH, D_2_O exchangeable), 6.5–7.9 (m, 5H, Ar-H), 8.4 (s, 2H, NH_2_, D_2_O exchangeable), 9.4 (s, 1H, NH, D_2_O exchangeable). ^13^C NMR (300 MHz, DMSO-d_6_): *δ* 113.1 (C-5 pyrimidine), 128.8–135.3 (aromatic Cs), 151.3 (C-3 triazole), 158.0 (C-6 pyrimidine), 159.9 (C-5 triazole), 164.1 (C-4 pyrimidine), 178.1 (C-2 pyrimidine). MS (EI): m/z: 304 [M^+^] (3.3%); 306 [M^+^ +2] (1.1%). Anal. Calcd for C_12_H_9_ClN_6_S (304.75): C, 47.29; H, 2.98; N, 27.58; Found: C, 47.50; H, 2.95; N, 27.65.

#### 4-Amino-6–(2,4-dichlorophenyl)-5-(1H-1,2,4-triazol-5-yl)pyrimidine-2-thiol (5c)

Buff powder, yield 80%, m.p. 213–215 °C.IR (KBr) v _max_ (cm^−1^): 3320–3438 (NH, NH_2_), 3040 (CH-Ar),1610 (C = N).^1^HNMR (300 MHz, DMSO-d_6_) *δ*:2.8 (s, 1H, SH, D_2_O exchangeable), 6.6–8.1 (m, 4H, Ar-H), 8.5 (s, 2H, NH_2_, D_2_O exchangeable), 9.7 (s, 1H, NH, D_2_O exchangeable). ^13^C NMR (300 MHz, DMSO-d_6_): *δ* 113.3 (C-5 pyrimidine), 128.4–136.7 (aromatic Cs), 151.5 (C-3 triazole),158.0 (C-6 pyrimidine), 159.5 (C-5 triazole), 164.2 (C-4 pyrimidine), 178.2 (C-2 pyrimidine). MS (EI): m/z: 338 [M^+^] (26.4%); 340 [M^+^ +2] (17.6%); 342 [M^+^ +4] (3%). Anal. Calcd for C_12_H_8_Cl_2_N_6_OS (339.20): C, 42.49; H, 2.38; N, 24.78; Found: C, 42.25; H, 2.42; N, 24.49.

### Screening of anti-cancer activity

#### The NCI-60 human tumour cell lines screen

Developmental Therapeutic Program (DTP), National Cancer Institute (NCI, MD, USA) (www.dtp.nci.nih.gov), had implemented an *in vitro* anticancer screening method.

60 different human tumour cell lines-representing leukaemia, melanoma, and malignancies of the lung, colon, brain, ovary, breast, prostate, and kidney are used in the operation of this screen.

##### Selection guidelines in NCI

According to the protocol of the NCI's drug assessment department in Bethesda, USA54 (National Cancer Institute, https://dtp.cancer.gov/), structures are typically chosen for screening based on their capacity to diversify the NCI small molecule compound collection.

All compounds (**1–5)** were chosen for single-dose testing. According to the described assay (National Cancer Institute, https://dtp.cancer.gov/discovery development/nci-60/), NCI60 single-dose testing is carried out in all 60 cell lines. Compounds were dissolved in DMSO: glycerol (91) and were kept in a freezer at −70° C. The synthesised compounds were introduced to the culture at a single concentration of 1 0 ^−5^ M and left to incubate for 48 h. A protein-binding dye called sulforhodamine B was used to measure end points (SRB).

##### Data analysis for a single dose

A mean graph showing the % growth of treated cells is used to represent the one-dose data. The value provided for the One-dose assay represents growth in comparison to the no-drug control and to the number of cells at time zero. This enables the identification of lethality (values between 0 and 100) as well as growth inhibition (values less than 0). A value of 100, for instance, indicates no growth inhibition. A value of 40 would indicate 60% growth inhibition. A value of 0 indicates no net growth over the course of the experiment. A value of −40 would indicate 40% lethality. A value of −100 indicates all cells are dead (National Cancer Institute, https://dtp.cancer.gov/ discovery development/nci-60/).

#### Five-dose NCI 60 cell screen

Compounds showing a considerable growth inhibition in the One-Dose Screen (eight or more cell lines with a growth% of 10 or less), compound **1c** is evaluated at five concentration levels (0.01, 0.1, 1, 10 and 100 μM) against the 60-cell panel.

According to the National Cancer Institute’s website (https://dtp.cancer.gov/discovery development/nci-60/), three response parameters-GI_50_ (Median Growth Inhibitory Concentration), TGI (Total Growth Inhibitory Concentration), and LC_50_ (Median Lethal Concentration) were calculated^87^ for compound **1c** (National Cancer Institute, https://dtp.cancer.gov/)^88^^.^

The molar concentration that inhibits cell growth completely is known as TGI value, whereas the concentration that causes a 50% reduction in cell growth is known as GI_50_ value.; on the other hand, The LC_50_ value denotes the concentration that results in a 50% loss of the initial cells. Furthermore, the average sensitivity of all cell lines to the compound is termed as the full panel mean graph midpoints (MG-MID). The complete panel mean graph midpoints refers to the average sensitivity of all cell lines to the chemical (MG-MID). A measure of a compound’s selectivity for a given cell line is the ratio created by dividing the full panel mean graph midpoints (MG-MID) concentration by the individual subpanel MG-MID concentrations (obtained as the average sensitivity of all the cell lines in a given subpanel to the test compound). Ratios greater than 6 denote high selectivity while ratios between 3 and 6 denote moderate selectivity whereas compounds not match either of these criteria were classified as non-selective^89^^.^

### In vitro PI3 kinases inhibitory activity assay

The inhibitory activity of compound **1c** was evaluated against PI3K (α, β and δ) using PI3 Kinase Activity/Inhibitor Assay Kit (CAT. # 17–493; Millipore Corporation, MA, USA) according to manufacturer’s regulations (see supplementary).

### Cell cycle analysis

Compound **1c** was selected as a potent candidate, its effect on cell cycle phases of HL60 and leukaemia SR cells was investigated using propidium iodide staining and flow cytometric analysis according to the cell cycle kit (Abcam ®, ab139418-)^90^^,^^91^^.^ Briefly, HL60 and leukaemia SR cells were cultured under optimum conditions. Afterwards, cells were treated by compound **1c** for 48 h. Then, they were harvested, washed with PBS, and cell fixation was performed using ethanol on ice. Additionally, cells were pelleted at 500 xg for 5 min and supernatant was gently aspirated. Furthermore, fixed cells were washed by phosphate-buffered saline (PBS) and resuspended in 200 µl 1 x propidium iodide + RNAse staining solution and incubated in dark for 30 min. Finally, stained cells were tested by flow cytometer using FACSCalibur (Becton Dickinson Biosciences, CA, USA). Finally, Cell Quest software (Becton Dickinson Biosciences) was used to determine cell cycle distribution.

### Apoptosis and necrosis assay

For more assessment of compound **1c**, apoptosis assay was performed using annexin V-FITC Apoptosis detection kit (Biovision, cat#: K101-25) followed by flow cytometric analysis^92^^,^^93^. Briefly, HL60 and leukaemia SR cells were cultured under optimum conditions followed by compound **I c** treatment for 48 h. then, washed in PBS, centrifuged, and resuspended in 500 µl of 1 x binding buffer. Moreover, 5 µl of annexin V-FITC, and 5 µl of propidium iodide were added and incubated in dark for 5 min. Finally, apoptotic cells were detected using flow cytometer as well as PI staining by emission signal detector. FACS Calibur flow cytometer (Becton Dickinson Biosciences) was used to analyse the cells’ apoptosis.

### Caspase 3 enzyme assay

One of the most significant downstream caspases, Caspase-3, is referred to as an effector caspase^94^^,^^95^. For further analysis, the effect of compound **1c** on caspase 3 enzyme level was measured using ELISA kit (Invitrogen, Catalog # KHO1091) according to the manufacturer’s instructions. Briefly, 100 µl of the standard diluent buffer to the zero standard wells. Additionally, 100 µl of standards and controls or diluted samples were added to the appropriate microtiter wells, then wells were covered with plate cover and incubated for 2 h at R.T. Afterwards, wells were aspirated gently, washed with washing buffer 4 times. Furthermore, 100 µl of caspase 3 detection antibody was added into each well except the blank wells, and the plate was covered and incubated for 1 h at R.T. Then, the aspiration and washing steps were repeated again. Finally, 100 µl of the stabilised chromogen solution were added, plate was incubated for 30 min at R.T., stop solution was used to stop the reaction and the developed colour was measured at wave length 450 nm using plat ELISA reader.

### Western blot analysis

To assess the effect of compound **1c** on protein expression of apoptotic and anti-apoptotic proteins levels, western blotting for selected candidate genes were performed^96^^,^^97^.Briefly, equal amounts (20 µg) of protein samples were mixed and boiled with SDS Loading buffer for 10 min, allowed to cool on ice and then loaded into SDS-polyacrylamide gel and separated by Cleaver electrophoresis unit (Cleaver, UK), transferred onto polyvinylidene fluoride (PVDF) membranes (BioRad) for 30 min using a Semi-dry Electro blotter (Biorad, USA) at 2.5 A and 25 V for 30 min. Additionally, the membrane was blocked by 5% non-fat dry milk solution in TBS-T for two hours at RT, in order to reduce non-specific protein interactions between the membrane and the antibody. Furthermore, the membrane was incubated overnight at 4 °C with primary antibodies, anti B-actin (Sigma ®), anti-Bax, anti-Bcl2, and anti-P53 (Abcam ®). The blots were then washed for three times (10 min each) with TBS-T. Finally, the membrane was incubated with the corresponding horse radish peroxidase (HRP)- linked secondary antibodies (Dako) for another hour at room temperature, followed by washing for three times (10 min each) with TBS-T. Then, the chemiluminescent reagent (ECL) was added according to the kit instructions and signals were detected by a CCD camera-based imager (Chemi Doc imager, Biorad, USA), and the bands intensities were measured by ImageLab (Biorad).

#### Statistical analysis

Data were presented as mean ± SD. GraphPad Prism 8.4.2 (679) software (San Diego, CA) was used to do a two-way ANOVA to determine the statistical significance of the differences. Statistical significance was defined as a value of *p* < 0.05.

### In vitro cytotoxic activity WI-38 cells (human lung fibroblast normal cell line)

The American Type Culture Collection provided WI-38 cells (normal lung cells). The cells were grown in DMEM with 10% heat-inactivated FBS (Hyclone), 10 µg/ml of insulin (Sigma), and 1% penicillin-streptomycin. The remaining chemicals and reagents were all from Sigma or Invitrogen. The MTT test was used to evaluate cytotoxicity, as described previously^98^. Graph pad Prism software (San Diego, CA) was used to estimate the 50% inhibitory concentration (IC_50_) from dose response curve graphic plots for each conc.

### Molecular modeling procedure

#### Molecular docking of Duvelisib and compound 1c into the binding site of human PI3Kδ:

The molecular docking was performed by AutoDock Vina^99^ using the crystal structure of human PI3Kδ co-crystallised with an inhibitor (PDB ID 5M6U). 3D structure of Duvelisib and compound **1c** were prepared using the Discovery Studio software (Accelrys Inc., San Diego, CA). Auto Dock Tools (The Scripps Research Institute, La Jolla, California, USA) was used to prepare the ligands and receptor as pdbqt files after removing water, adding polar hydrogen atoms, and Gasteiger charges, respectively. The docking grid box size used was adjusted accordingly to encompass the interaction site. An exhaustiveness value of 8 was used while keeping the other parameters with their default values. The best docking pose (most stable) was selected for binding mode comparison. Visualisation of ligand-protein non-covalent interactions was performed using Discovery Studio software. The schematic 2-D representations of enzyme-ligand complexes were generated using Discovery Studio software.

## Results and discussion

### Chemistry

**Scheme 1.**
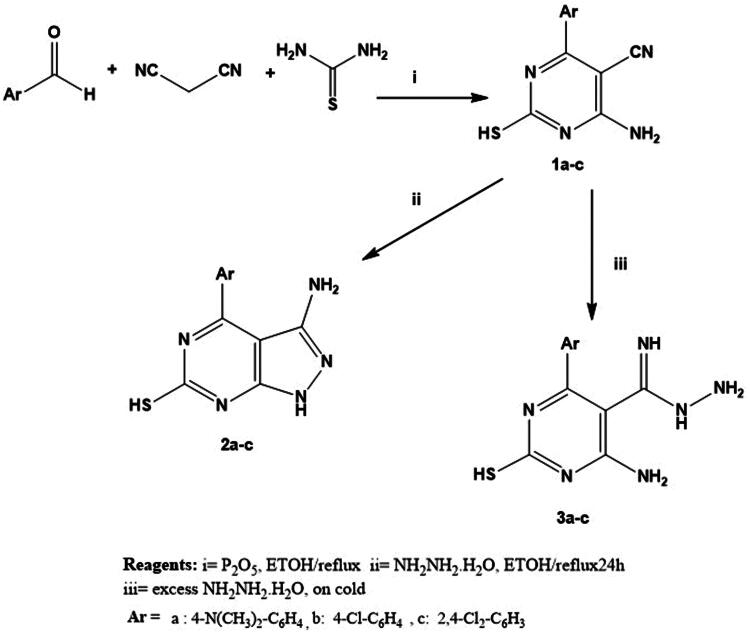
Synthesis of compounds **(1a-c) -(3a-c)**

The provided starting compounds **1a-c** were prepared via condensation of the appropriate aldehyde with malononitrile and thiourea applying a multi-components one step reaction in presence of P_2_O_5_ as a catalyst^86^. Malononitrile and aromatic aldehydes are likely to undergo a Knoevenagel condensation reaction as the reaction progresses, producing aryl methylene malononitrile. These further reacted with thiourea to get the desired product (Scheme 1). Phosphorus pentoxide is a cheap chemical reagent that tends to absorb water molecules and makes the rate of reaction is accelerated as one water molecule is created as the reaction proceeds, producing the required products promptly and with a quantitative yield.

**Scheme 2.**
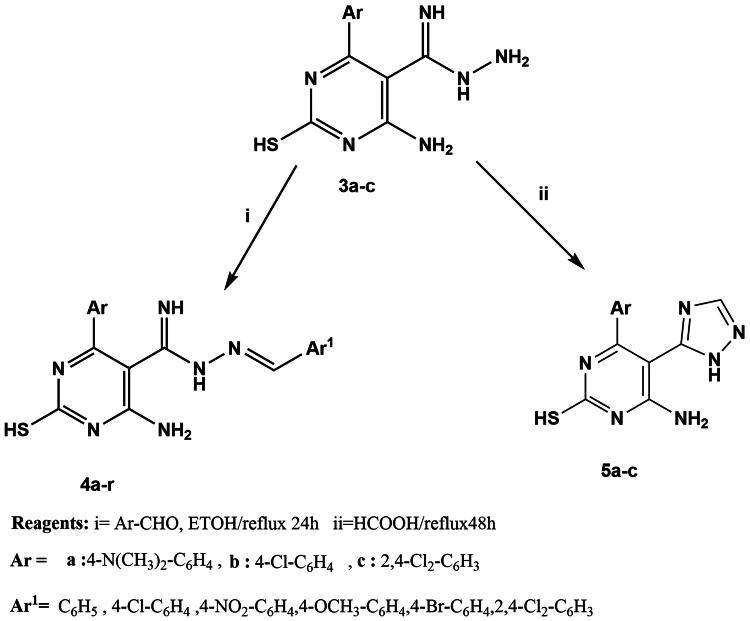
Synthesis of novel hydrazone derivatives (**4a-r)** and triazole derivatives (**5a-c)**.

On the other hand, stirring of **1a-c** with an excess of hydrazine hydrate at room temperature^100^^,^^101^ afforded carboximidohydrazide derivatives **2a-c**. Additionally, compounds **1a-c** were refluxed with hydrazine hydrate^102^ in absolute ethanol to afford pyrazolo pyrimidine derivatives **3a-c** (Scheme 1). In addition, in scheme 2, hydrazide derivatives **3a-c** were refluxed independently with a series of aromatic aldehydes^103^^,^^104^ in absolute ethanol to afford the title compounds **4a-r.** Triazolo derivatives **5a-c** were prepared by reaction of **3a-c** with formic acid^100^^,^^101^ (Scheme 2). Both microanalytical and spectral data confirm the structures of the synthesised compounds.

### Screening of anti-cancer activity

#### The NCI-60 human tumour cell lines screen (interpretation of one-dose data)

**Figure 6.**
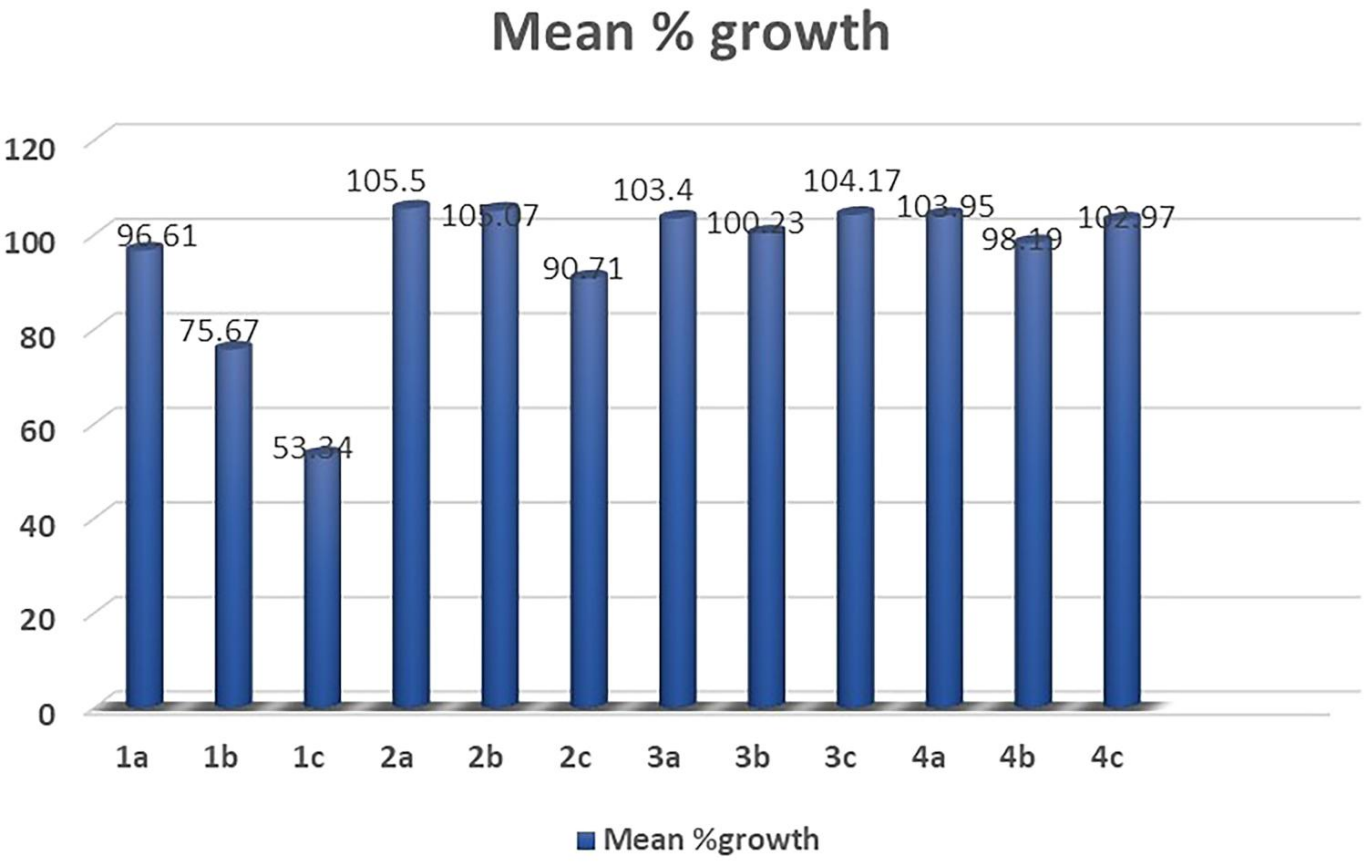
Mean % growth of compounds **1a- 4c**.

**Figure 7.**
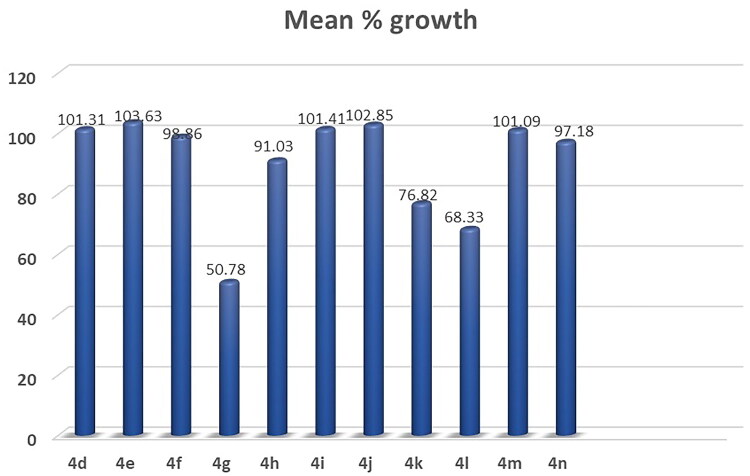
Mean % growth of compounds **4d- 4n**.

**Figure 8.**
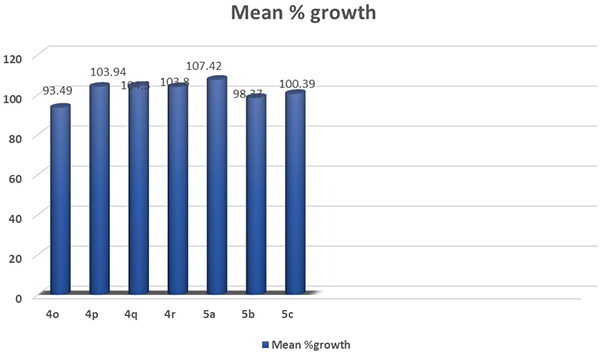
Mean % growth of compounds **4o- 5c**.

All the synthesized compounds **(1a-c, 2a-c, 3a-c, 4a-r and 5a-c)** were selected by the NCI to be tested for their *in vitro* anti-cancer activity. At a single high dose of 10 M, the selected compounds were assessed against the full NCI 60 cell lines panel representing leukaemia, melanoma and cancers of lung, colon, central nervous system, ovary, kidney, prostate as well as breast. The data were reported as mean graph of the percent growth of the treated cells (Supplementary data, Supplementary Tables S1-S5, Figures 1–6) (Figures 6–8).

**Table 1.** Selectivity of compound **1c** on nine human cancer cell types.

Panel	Cell line	GI_50_			TGI	LC_50_
		Conc. Per cell line, Subpanel MID^b^,Selectivity ratio				
leukaemia	CCRF-CEM	0.3	0.33	39	16.10	>100
	HL-60(TB)	0.08			1.82	>100
	K-562	0.14			>100	>100
	MOLT-4	0.7			43.90	>100
	RPMI-8226	0.6			27.50	>100
	SR	0.14			23.70	>100
Non-small cell lung cancer	A549/ATCC	25.8	18.14	0.7	77.40	>100
	EKVX	14.3			29.90	62.60
	HOP-62	18.2			40.20	88.60
	HOP-92	19.8			56.40	>100
	NCI-H226	16.5			36.80	82.00
	NCI-H23	13.1			28.30	60.90
	NCI-H322M	18.1			38.40	81.30
	NCI-H460	22.1			56.80	>100
	NCI-H522	15.4			30.70	61.30
Colon cancer	COLO 205	19	10.9	1.2	36.50	70.00
	HCC-2998	13			25.90	51.50
	HCT-116	0.06			17.90	63.60
	HCT-15	0.08			17.90	97.90
	HT29	13.8			30.80	68.90
	KM12	19			39.00	80.10
	SW-620	12			30.70	78.60
CNS Cancer	SF-268	18.1	12.4	1.04	52.70	>100
	SF-295	10.4			22.60	49.00
	SF-539	10.8			23.80	52.40
	SNB-19	17			50.00	>100
	SNB-75	4.90			25.50	79.90
	U251	13.4			30.40	68.90
panel	Cell line	GI_50_			TGI	LC_50_
		Conc. Per cell line, Subpanel MID^b,^Selectivity ratio				
melanoma	LOX IMVI	1.10	10.7	1.2	15.10	40.00
	MALME-3M	15.3			32.70	69.70
	M14	2.42			28.00	93.70
	MDA-MB- 435	10.5			38.00	>100
	SK-MEL-2	17.5			34.20	66.80
	SK-MEL-28	15.2			30.40	60.70
	SK-MEL-5	5.56			19.50	45.00
	UACC-257	14.7			31.50	67.20
	UACC-62	13.8			28.20	57.60
Ovarian cancer	IGROV1	19.2	19.2	0.7	41.00	87.60
	OVCAR-3	18.1			47.60	>100
	OVCAR-4	32			>100	>100
	OVCAR-5	16.4			32.20	63.20
	OVCAR-8	21.8			74.70	>100
	NCI/ADR- RES	0.7			26.20	>100
	SK-OV-3	26.3			62.60	>100
Renal cancer	786-0	17.5	14.2	0.9	41.60	99.10
	A498	19.8			39.40	78.50
	ACHN	16.5			32.00	62.10
	CAKI-1	18.4			45.70	>100
	RXF 393	12.7			25.70	52.20
	TK-10	21.5			50.20	>100
	UO-31	14.7			28.10	53.60
Prostate cancer	PC-3	11.1	13	0.99	48.20	>100
	DU-145	14.9			29.20	57.40
Breast cancer	MCF7	0.12	11.4	1.13	31.10	>100
	MDA-MB- 231/ATCC	13.1			27.60	58.10
	HS 578 T	18.8			92.60	>100
	BT-549	14.9			30.50	62.70
	T-47D	9.87			32.30	>100
	MDA-MB- 468	11.4			25.20	55.70
Mida	12.9					

Molar concentrations producing 50% growth inhibition (GI_50_), molar concentrations producing total growth inhibition (TGI), molar concentrations producing 50% cellular death (LC_50_), the average sensitivity of all cell lines towards the test agent (µM) (MID^a^).

The obtained results of the tested thiopyrimidine analogs **1–5** showed broad spectrum anticancer activity. Considering the activity towards particular cell lines; Compound **1c** has high activity with a mean value of 53.34. In details, compound **1c** showed high activity against 12 of the tested cell lines with % growth values ranging from 2.54% to 25.29%, lethal effect against 2 of the tested cell lines with % growth values ranging from −18.77 to −49.21, moderate activity against 13 cell lines with % growth values ranging from 31.58% to 49.84%, low activity against 33 cell lines with % growth values ranging from 51.01% to 136.57% (Supplementary Table S1, Figure 1).

It showed high anticancer activity against leukaemia (CCRF-CEM, HL-60(TB), MOLT-4, RPMI-8226 and SR) with tumour growth percentages of (7.65, 5.30, 6.11, 9.51, 2.54, respectively), colon cancer (HCT-15 and HT29) with growth percentages of (10.91 and 24.68, respectively), melanoma(MALME-3M and MDA-MB-435) with growth percentages of (4.96 and 25.29, respectively), ovarian cancer (OVCAR-3 and OVCAR-8) with growth percentages of (17.15 and 5.74, respectively) and renal cancer(ACHN) with growth percentage 19.59. It recorded complete cell death for colon cancer cell line HCT-116 with growth percentage −18.77 and melanoma cell line LOX IMVI with growth percentage −49.21. It also exhibited moderate potency towards leukaemia cell line K- 562 with growth percentage 36.86, non-small cell lung cancer line NCI-H522 with growth percentage 49.42, colon cancer cell lines (KM12 and SW-620) with growth percentages of (49.53 and 45.69, respectively), CNS cancer cell lines (SF-539, SNB-75, U251) with growth percentages of (39.87, 36.51 and 38.84, respectively), melanoma cell line M14 with growth percentage 49.84, ovarian cancer cell line NCI/ADR-RES with growth percentage 37.91, prostatic cancer (PC-3) with growth percentage 49.77 and breast cancer (MCF7,BT-549 and MDA-MB-468) with growth percentages of (31.58,42.10 and 34.98, respectively).

Compound **1b** recorded moderate activity against leukaemia (RPMI −8226), non-small cell lung cancer (HOP-92), CNS cancer (SNB-750) and renal cancer (UO-31) cell lines with growth percentages of (47.20, 43.53, 42.20and45.35, respectively) and low activity against 56 cell lines (Supplementary Table S1, Figure 2).

Compounds **1a, 2a, 2b, 2c, 3a, 3b, 3c, 5a and 5b** exhibited weak activity on all 60 cancer cell lines tested (Supplementary Table S1,S2).

**Table 2.** IC_50_ (µM)^a^ of compound **1c**.

Compound	PI3Kα	PI3Kβ	PI3Kδ
**1c**	0.880 ± 0.005	0.550 ± 0.002	0.0034 ± 0.004
**Duvelisib**	1.602 ± 0.003	0.085 ± 0.004	0.0025 ± 0.002

^a^ mean of two independent replicates ± SD.

Compound **5c** recorded moderate activity against non-small cell lung cancer cell line EKVX with growth percentage 49.98 (Supplementary Table S2, Figure 3).

The results demonstrate the anticancer results for compounds **4a-r**. Compounds **4a, 4b, 4c, 4d, 4e** and **4f** exhibited weak activity on all 60 cancer cell lines tested (Supplementary Table S5).

Compound **4g** showed high activity against 11 of the tested cell lines with % growth values ranging from 1.22% to 30.62%, lethal effect against 4 of the tested cell lines with % growth values ranging from −0.44%to-34.30%, moderate activity against 11 cell lines with % growth values ranging from 31.24% to 49.66%, low activity against 33 cell lines with % growth values ranging from51.39% to 104.62% (Supplementary Table S4, Figure 4).

It exhibited significant anticancer activity against non-small cell lung cancer (HOP-62 and NCI-H460) with tumour growth percentages of (30.23 and 16.47, respectively), colon cancer (HCT-116) with growth percentage 30.62, CNS cancer (SF-539 and U251) with growth percentages of (11.40 and 13.22, respectively), melanoma(LOX IMVI) with growth percentage 22.56,ovarian cancer (OVCAR-8) with growth percentage 20.38, renal cancer(ACHN, RXF-393 and UO-31) with growth percentages of (23.28, 8.96 and 29.45, respectively) and breast cancer(MDA-MB-231/ATCC)) with growth percentage 1.22.It recorded complete cell death for non-small cell lung cancer cell lines HOP-92 and NCI-H226 with growth percentages −0.44 and −0.68, respectively, CNS cancer cell line SNB-75 with growth percentage −34.30 and breast cancer cell line HS 578 T with growth percentage −6.98.It also showed moderate potency towards leukaemia cell lines SR with growth percentage 46.39, non-small cell lung cancer (A549/ATCC) with growth percentage31.63, CNS cancer (SF-268 and SF-295) with growth percentages of (49.28, 35.61, respectively), ovarian cancer (OVCAR-4) with growth percentage 37.46, renal cancer (786–0, CAKI- 1) with growth percentages of (41.67and 31.24, respectively), prostatic cancer(DU-145) with growth percentage 48.70 and breast cancer (MCF7, T-47D and MDA-MB-468) with growth percentages of (42.23, 49.42 and 49.66,respectively).

Compound **4k** showed significant anticancer activity against Non-Small Cell Lung Cancer cell line HOP-92 with growth percentage 18.97, it also recorded complete death of growth for breast cancer cell line MDA-MB-231/ATCC with growth percentage −1.21 and moderate anticancer activities for leukaemia cell line CCRF-CEM, CNS cancer cell line SNB- 75, renal cancer cell line UO-31 and breast cancer cell line HS 578 T with growth percentage from 35.46 to 47.83 (Supplementary Table S4, Figure 5).

Compound **4l** exhibited significant anticancer activity against CNS cancer (SNB- 75), renal cancer (A498), and breast cancer (T-47D) cell lines with growth percentage 26.06, 6.77 and 28.14, respectively. It also recorded moderate anticancer activities against leukaemia (RPMI-8226), melanoma (MALME-3M, UACC-62), ovarian cancer (IGROV1), renal cancer (UO-31 CAKI-1, RXF 393), prostate cancer (PC-3) and breast cancer (HS 578 T); growth % = 46.53, 43.36, 43.08, 44.88, 43.20, 37.0, 31.19, 48.02and36.43, respectively) (Supplementary Table S4).

Compound **4o** showed significant anticancer activity against non-small cell lung cancer cell line HOP-92 with growth percentage 2.04 (Supplementary Table S5, Figure 6).

Compounds **4h, 4i, 4j, 4 m, 4n, 4p, 4q** and **4r** exhibited weak activity on all 60-cancer cell line tested (Supplementary Table S5).

#### Five-dose results

**Figure 9.**
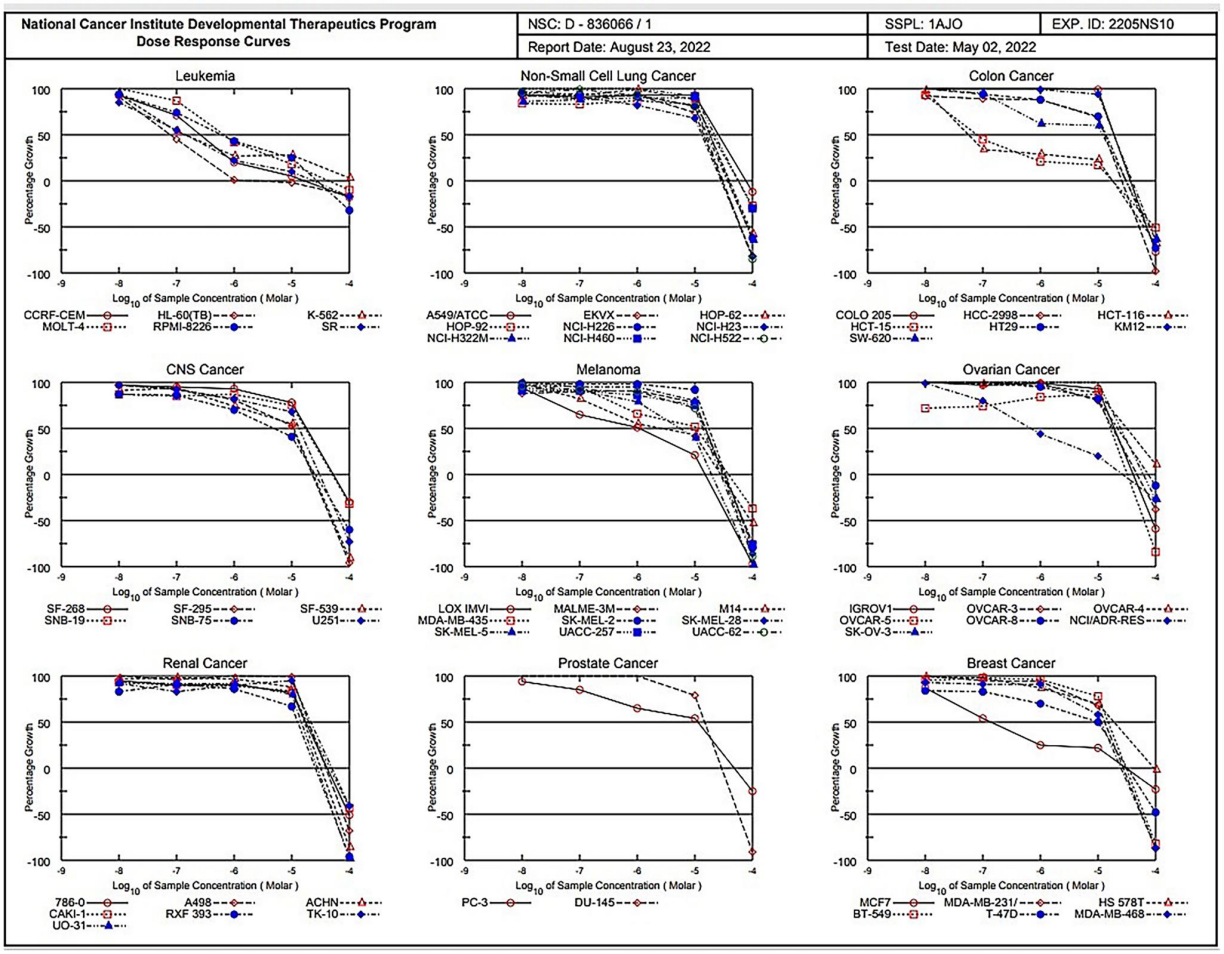
dose response curves of compound **1c** against 60 cancer cell lines.

The most active compound **(1c**) satisfied the predetermined threshold growth inhibition criteria and were further selected for the five-dose assay where cytotoxic activity of this compound was tested *in vitro* against the 60 human tumour cell lines panel derived from nine cancerous diseases at 10-fold dilution of 5 concentrations ranging from 1 0 ^−4^ to 10^−8^M to determine their GI_50_, TGI, and LC_50_ (Table 1, (Figure 9).

For each cell line, the median growth inhibition, total growth inhibition, and median lethal response parameters were computed (Table 1). The GI_50_ (Growth inhibitory activity) value relates to the compound’s molar concentration causing 50% inhibition of net cell growth. The molar concentration that causes total growth inhibition is defined as the total growth inhibition (TGI) value (cytostatic activity). The molar concentration that causes 50% of all cells to die is known as the LC_50_ (median lethal concentration) value (cytotoxicity parameter). Moreover, mean graph mid-points (MG-MID) were calculated for compound **1c** against all the individual subpanels and full panel cell lines, which is the average activity parameter over all cell lines .The parameters for delta and range were also determined. The means of the log^,^s of each individual GI, TGI, and LC value were then calculated to yield the log10 GI50, log10 TGI50, and log10 LC50. Negative values indicate the most sensitive cell lines. Compounds having log10 GI50 values ≤ −4 are declared to be active (Supplementary biology, Figure 7). Results of the five-dose assay demonstrate that compound **1c** has broad spectrum anti-cancer activity against the nine cancerous subpanels tested with effective growth inhibition full panel GI50 (MGMID) value of −5.17 μM and cytostatic activity full panel TGI (MG-MID) values of −4.48 μM. However, the LC50 values (MG-MID) (cytotoxicity) were −4.11 μM for tested cell lines. As revealed from their GI_50_, TGI and LC5_0_ values, it recorded strong cytotoxic activity against all 60 human cancerous cell lines tested, where the log10 GI50 was varying from −4.50 to −7.25

Regarding sensitivity of individual cell lines (Table 1), compound **1c** displayed distinguished sensitivity profile towards leukaemia cell lines with GI_50_ range of 0.08–0.7 μM and colon cancer (HCT-116 and HCT-15) with GI_50_ 0.06 and 0.08 μM, respectively. Among the tested cancer cell lines, leukaemia was the most susceptible to the influence of **1c** (GI_50_ [MG-MID] 0.33).

##### Selectivity assessment

Assessment of selectivity of a compound depends on the ratio obtained by dividing the full panel MID ^a^ by their individual subpanel MID ^b^ (µM) where MID^a^ is the average sensitivity of all cell lines towards the test agent (µM). Ratios between 3 and 6 indicate moderate selectivity, ratios > 6 refer to higher selectivity towards the corresponding cell line, compounds not meeting either of these criteria are considered to be non-selective^105^^.^

Compound **1c** was observed to have broad spectrum antitumor activity against the nine tumour subpanels tested with selectivity ratio ranging from 0.7 to 39 at the GI50 level. It recorded high selectivity towards leukaemia (selectivity ratios; 39). In addition to its antileukemic effect, it has been demonstrated to be non-cytotoxic (LC50 > 100 M) and low selectivity towards non-small cell lung cancer, colon cancer, CNS cancer, melanoma, ovarian cancer, renal cancer, prostate cancer and breast cancer cell-lines (selectivity ratios; 0.70, 1.2,1.04,1.2,0.7,0.9,0.99 and 1.13 respectively), as revealed in **Table 1**.

## Result analysis and structural variation

### Regarding the SAR of compounds

First, the results of 4-Amino-2-mercaptopyrimidine-5-carbonitrile analogues **1a-c** with different aryl substituents showed that the anticancer activity was consistently correlated with the lipophilicity and/or electrical properties of the substituent groups on aryl ring. Regarding the characteristics of the substituent groups, tested compounds were ordered in the following order by their level of action against various cell lines: (2,4-Cl2 > 4-Cl > N(CH_3_)_2_).

Only compound with electron-withdrawing group and moderate size such as the chloro derivative **1c** (with 2,4-dichloro phenyl moiety) has significant broad spectrum anticancer activity against seven cancerous subpanels, sufficient to qualify this compound for 5-dose assay. Compound **1c** possesses anticancer potency higher compared to one with electron-donating group **1a** (N, N-dimethyl phenyl moiety) and compound with 4-chloro phenyl moiety **1b.** Also compound **1c** showed higher selectivity towards leukaemia (CCRF-CEM). **(**Table 2). As evidenced by pyrimidine analog **1c**, the anticancer activity of the pyrimidine is improved by the addition of a more electronegative and lipophilic substituent.

Second, keeping the substituted phenyl at position-6 and replacing the 5-cyano group of compounds **1a-c** with small (carboximidohydrazide group) or large moiety (aryl methylidene or triazole ring) in compound **2a-c, 4a-r** and **5a-c**, respectively results in decrease or abolishing the anticancer activity. However, benzylidene derivative **4g** exhibited significant anticancer activity towards seven cancerous subpanels, but its activity was lower than that of **1c** derivative.

Third, keeping the substituted phenyl at position-6 and cyclisation of the cyano and amino group of compounds **1a-c** to the corresponding pyrazolopyrimidine analogues **3a-c** led to significant decrease or abolishing the anticancer activity.

### In vitro PI3 kinases inhibitory activity assay

To examine the putative mechanism underlying compound **1c’**s potent anticancer effect, its inhibitory capacity against PI3Kα, β and δ was tested. The IC_50_ values of compound **1c** were compared to those of the reference PI3K inhibitor Duvelisib. Compound **1c** showed potent inhibitory activity against PI3Kα, β and δ at sub micromolar concentrations (IC_50_ = 0.88, 0.55, and 0.0034 μM, respectively). Compound **1c** showed comparable activity to Duvelisib against PI3Kδ (IC_50_ = 0.0034 and 0.0025 μM, respectively).

### Cell cycle analysis

**Figure 10.**
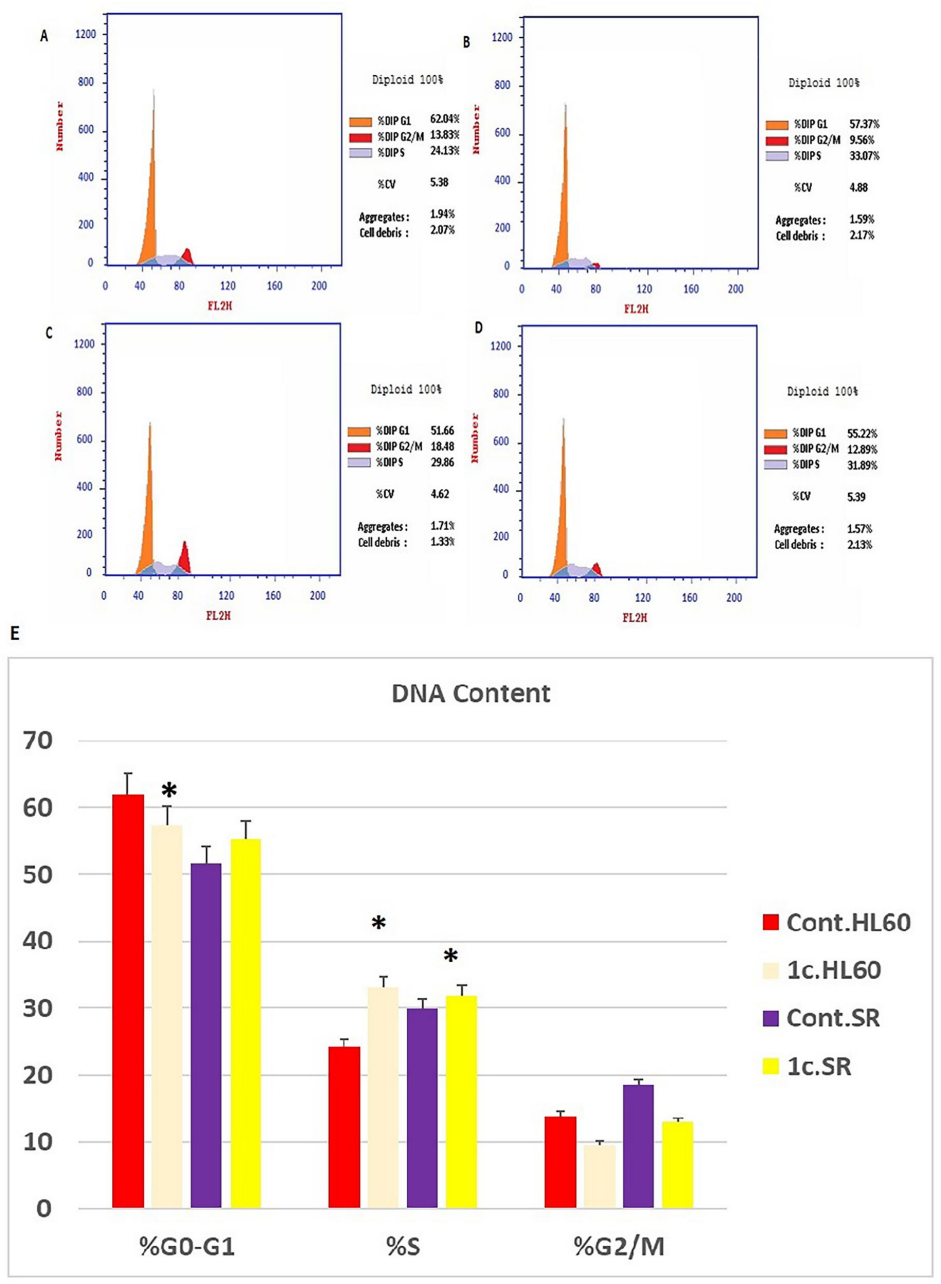
Flow cytometric analysis of cell cycle phases after compound **1c** treatment in HL60 and leukaemia SR cell lines. (A & C) The histograms represent the cell cycle distribution of control (HL60 & leukaemia SR cells). (B & D) The histograms represent the cell cycle distribution of compound **1c** treated cells. (E) A column graph represents the percentage of cells in each phase of the cell cycle in compound **1c** treated.

Numerous anticancer compounds cause either cell cycle arrest, apoptosis, or a combination of cycle arrest and apoptosis to exert their growth inhibitory effect^106^^,^^107^. ^.^In addition, controlling the cell cycle and apoptosis is a successful strategy for creating cancer therapies^108^. As a result of the cytotoxic activity of compound **1c** displayed distinguished sensitivity profile towards leukaemia cell lines. The effect of compound **1c** on the most sensitive cell line, HL60 and leukaemia SR cells was studied.We tested its effects on the cell cycle phases. It was crucial to determine whether cell cycle arrest was the cause of inhibition of cell growth. Our results have shown that the percentage of arrested cells has significantly increased from 24.13% and 29.86% of the untreated control HL60 and leukaemia SR cells to 33.07%, 31.89% of compound **1c** treated cells, respectively at the S phase. Additionally, percentage of arrested cells has significantly increased from 51.66% of the untreated control leukaemia SR to 55.22% of compound **1c** treated cells, respectively at the G1 phase. However compound 1c led to a decrease in G0/G1 HL60 cells from 62.04% to 57.37% of its control cells (Figure 10).

Consequently, our results had shown that compound **1c** exerted its effect mainly via triggering S phase arrest of the cell lines HL60 and Leukaemia SR respectively. Hence, The antiproliferative effect of the compound is attributed to its potent apoptotic activity. The results show that compound **1c** arrested cell cycle at the S phase.

### Apoptosis and necrosis analysis

**Figure 11.**
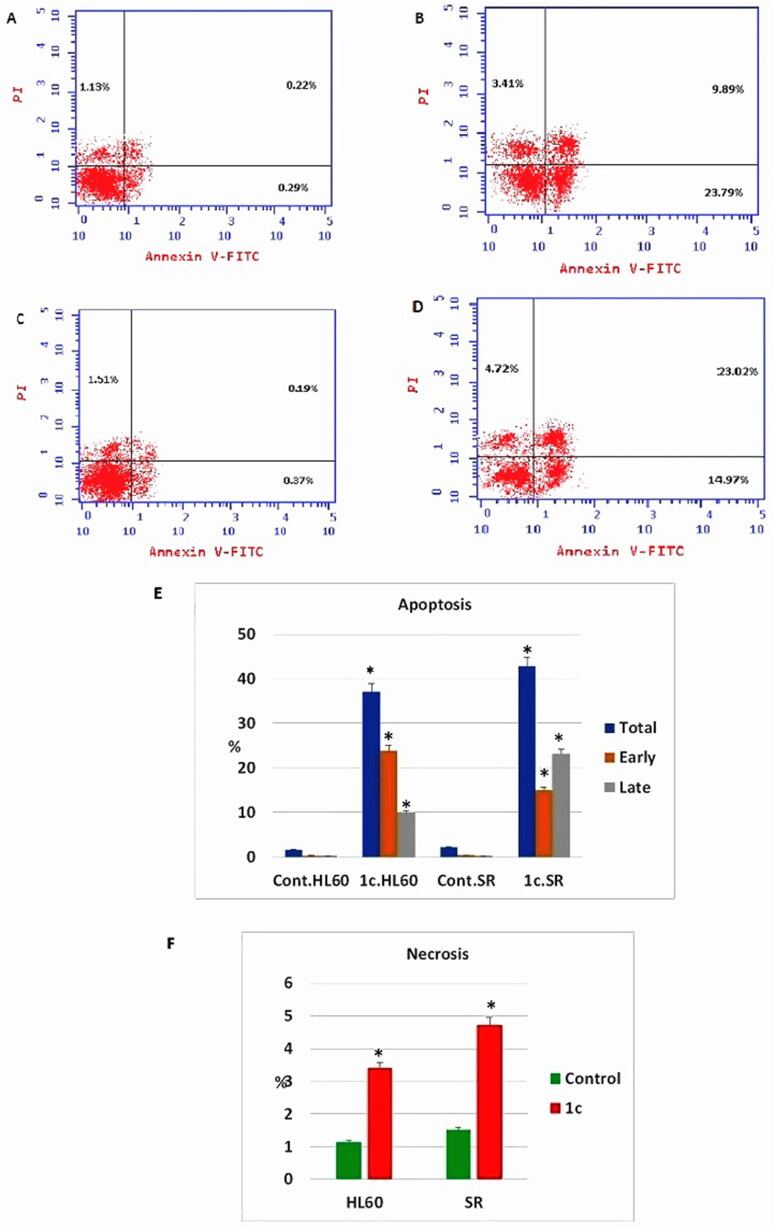
Apoptosis assay after treatment with compound **1c** on HL 60 and leukaemia SR cells. (A & C) The cytograms show apoptosis pattern of the control cells (HL60 & leukaemia SR). (B &D) The cytograms show apoptosis pattern of compound **1c** treated cells. (E) The column graph represent quantification of annexin v positive cells for compound **1c** relative to control showing total, early & late apoptosis (F) The column graph represents necrosis percent in compound 1**c** treated cells compared to their untreated control. * *P* < 0.05 indicates significant differences compared with control using unpaired student t test.

The effect of compound **1c** on apoptosis was assessed using an annexin-V∕ propidium iodide (PI) staining assay. Early and late apoptosis of compound **1c** treated HL60 had been significantly increased from 0.29% and 0.22% to 23.79% and 9.89%, respectively. Moreover, early, and late apoptosis showed a significant increase in compound **1c** treated leukaemia SR cells from 0.37% and 0.19% to 14.97% and 23.02%, respectively. Consistent with our findings, necrosis percentage showed a significant increase from 1.13% to 3.41% in compound **1c** treated HL60 cells as well as from 1.51% to 4.72% in compound **1c** treated leukaemia SR cells (Figure 11).

### Caspase 3 determination

Caspase-3 is a crucial indicator of apoptosis. In the apoptotic cascade, caspase-3 is a downstream effector cysteine protease. Caspase-3 overexpression and loss of expression have been observed in a variety of malignant cancers^109^.

**Figure 12.**
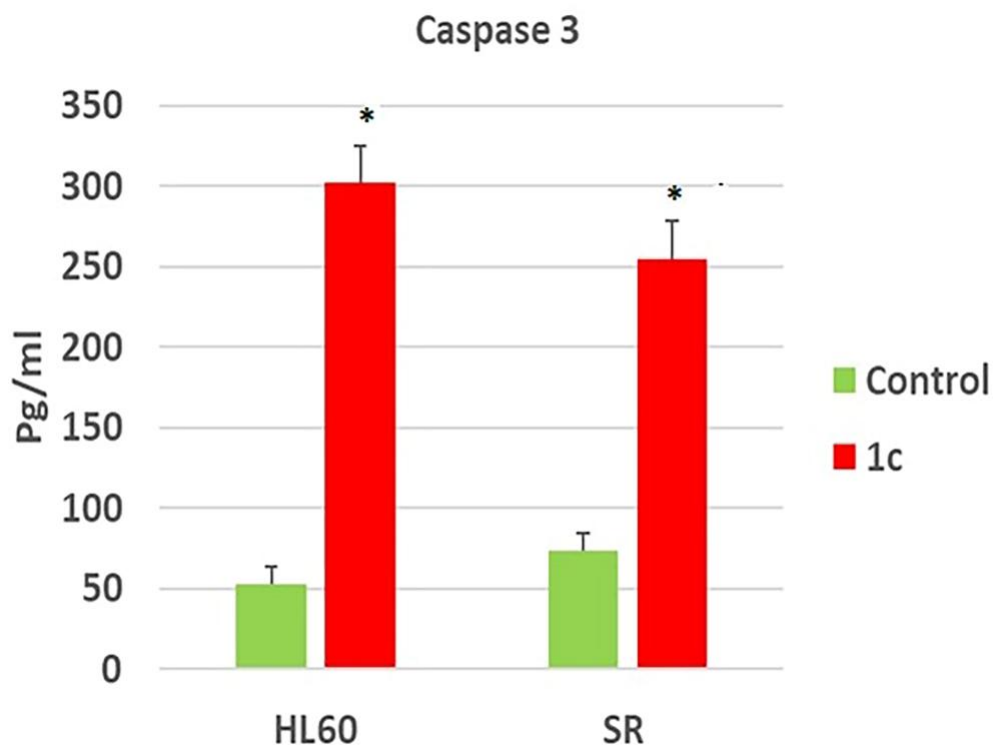
Effects of compound **1c** on caspase 3 level in HL60 and leukaemia SR cells. Values are reported as mean SEM. * *P* < 0.05 indicates significant difference from the untreated control using unpaired student t test.

To confirm the effect of compound **1c** on apoptosis, its effect on caspase 3 level was tested. Our results have shown that compound **1c** has increased caspase 3 level significantly on compound **1c** treated HL 60 cells by (5.6) folds in regard to their untreated control. However, its level was increased significantly by (3.4) folds in compound **1c** treated leukaemia SR cells when compared to their untreated control (Figure 12). These findings suggested that **1c** caused apoptosis by activating caspase-3, which was validated by cell cycle analyses and annexin V tests.

### Western blotting analysis

The family of Bcl-2 proteins is primarily in charge of synchronising the mitochondrial apoptotic process and divided into two main categories: group I proteins with antiapoptotic properties such as Bcl2 protein and group II proteins with apoptotic properties such as Bax protein^110^. As a result, apoptosis can be successfully induced by inhibiting group I proteins and/or activating group II proteins.

A nuclear transcription factor called p53 promotes apoptosis. Since the p53 gene contains loss of function mutations in more than 50% of human malignancies, p53 has been regarded as one of the traditional types of tumour suppressors. A cell cycle arrest caused by activated p53 allows for DNA repair and/or apoptosis^111^.

**Figure 13.**
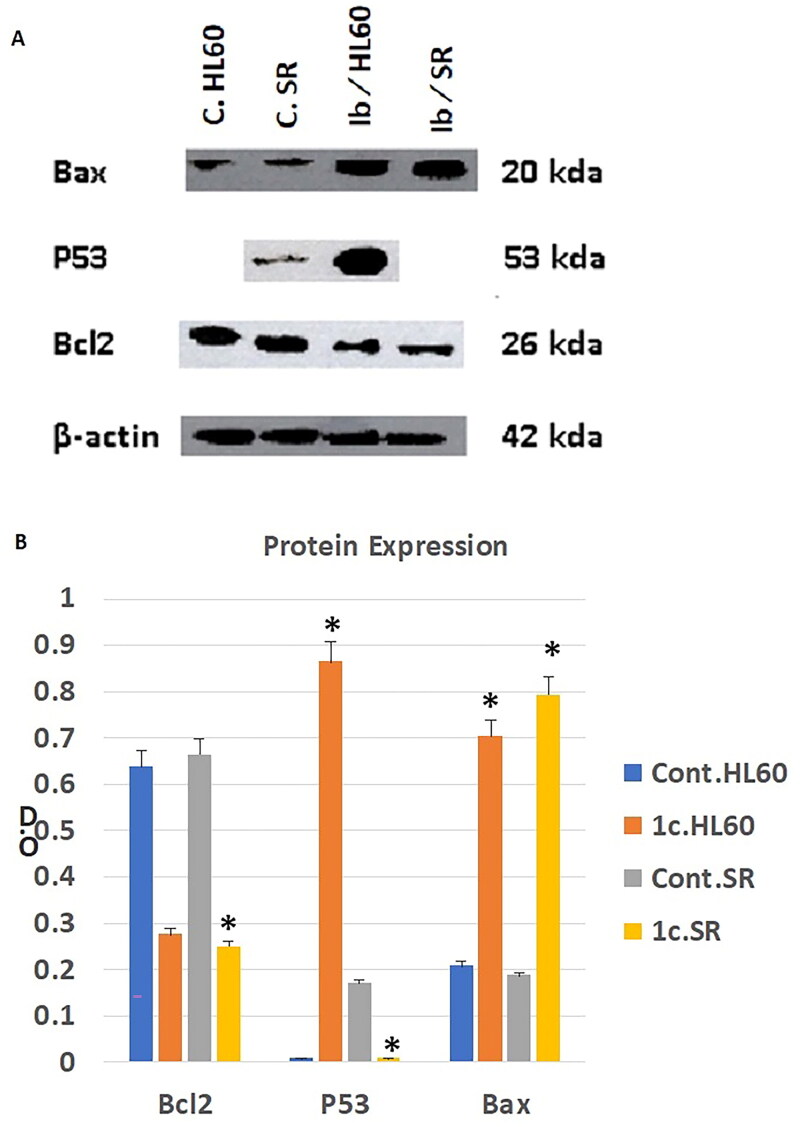
The effect of compound **1c** on Bcl2, P53, and Bax protein levels on HL60 and leukaemia SR cells. (A) Western blotting analysis of Bcl2, P53, Bax protein expression in HL60 and leukaemia SR cells treated with compound **1c** compared to their untreated cells as negative control. B- actin was used as loading control. (B) The column graph represents relative expression of Bcl2, P53, and Bax protein levels after compound **1c** treatment in HL60 and leukaemia SR cell lines in comparison to their untreated controls. * significance at *P* < 0.05.

We then examined apoptosis-related indicators to provide additional evidence for apoptosis. To test the anti-tumour effects of compound **1c** on protein level, we tested the effect of our candidate compound on the expression level of Bcl2, Bax and P53 proteins by western blotting technique. As shown in figure 13 we found that compound **1c** significantly inhibited the expression of the tumorigenic protein Bcl2 on both HL60 and leukaemia SR cells when compared to their untreated control. On the other hand, we get opposite results regarding the apoptotic proteins Bax and P53. Our candidate compound has significantly increased the expression of Bax protein on both HL60 and leukaemia SR cells when compared to their untreated control. However, it increased the expression of P53 protein significantly only in HL60 cells when compared to their untreated control.

These results demonstrated that compound **1c** caused apoptosis in HL60 and leukaemia SR cells by suppressing the antiapoptotic Bcl2 protein, activating the apoptotic Bax protein in both types of cells, and activating the P53 protein in HL60 cells.

### In vitro cytotoxicity towards human normal WI-38 cells

The potent compound **1c** was evaluated for cytotoxic effect against human normal lung fibroblast cell line (WI-38 cells), to investigate its safety adopting the MTT assay procedures^98^. The results, expressed as (IC_50_) values (Table 3).

**Table 3.** Cytotoxic activity (IC_50_) of compound **1c** against WI-38 cell line.

^a^IC_50_(μM)	
**Compound**	**WI-38**
**1c**	43.6 ± 1.20
**Duvelisib**	22.80 ± 0.65

^a^ Three independent experiments were performed for each concentration. ^b^IC_50_ values represent mean ± SD of three experiments.

The tested compound **1c** showed non-significant cytotoxic effect towards normal WI-38 cells (IC_50_ = 43.6 ± 1.20 μM) and was less cytotoxic than anticancer drug duvelisib (IC_50_ = 22.80 μM), thereby providing a high-safety profile as anti-proliferative agents.

### Molecular modeling

#### Docking study to evaluate binding of compound 1c to hPI3Kδ:

The PI3K (phosphoinositide 3-kinase) family of lipid kinases is divided into three classes, PI3K class I, class II, and class III. However, PI3K class I is the most well-defined class, and gets activated by cell surface receptors to produce phosphatidylinositol-3,4,5-trisphosphate (PIP3)^66^^.^ PI3K class I is subdivided into four different isoforms PI3Kα, PI3Kβ, PI3Kδ, and PI3Kγ. PI3Kδ is expressed primarily in leukocytes and regulates a wide range of cellular activities such as cell survival, proliferation, differentiation, and cell trafficking^112^. PI3Kδ is now a validated and a promising target for cancer therapy, with the approval of PI3Kδ inhibitors for clinical use.

The role of PI3Kδ in B-cell activation has been investigated in haematologic malignancies driven by the AKT pathway, resulted in the approval of the selective PI3Kδ inhibitor Idelalisib in 2014 for the treatment of follicular B-cell non-Hodgkin lymphoma and chronic lymphocytic leukaemia^113^. Another structurally related PI3Kδ inhibitor duvelisib was approved in 2019 for the treatment of hematological tumours^114^ (Figure 4). However, the use of these approved oral PI3Kδ inhibitors as anticancer agents comes with serious adverse effects including hepatic toxicity, GI toxicity, several infections, and intestinal perforation^115^. Our drug discovery approach was focused on the discovery of another class of compounds with better safety profile.

The crystal structure of human PI3Kδ co-crystallised with clinical candidate IC87114 shows that the ligand adopts a propeller-shaped conformation in which the quinazolin-4-one moiety is sandwiched into an induced hydrophobic specificity pocket between Trp760 and Met752^116^. Previous studies^117^^,^^118^ suggest that increasing potency could be achieved via ligand side chain that interact with the affinity pocket. This pocket is surrounded by the side chains of Ser831, Glu826, Val827 and Val828. Thus, our efforts were based on introducing functional groups that could interact with the amino acids present in this region. In this study, we used molecular docking to drive SAR. The molecular docking was performed by AutoDock Vina using the crystal structure of human PI3Kδ co-crystallised with an inhibitor (PDB ID 5M6U).

Validation for the docking parameters was performed by re-docking the co-crystallised inhibitor into the binding pocket of human PI3Kδ. The binding mode was assessed by comparing the modelled structure’s binding mode to that of the crystal structure.

**Figure 14.**
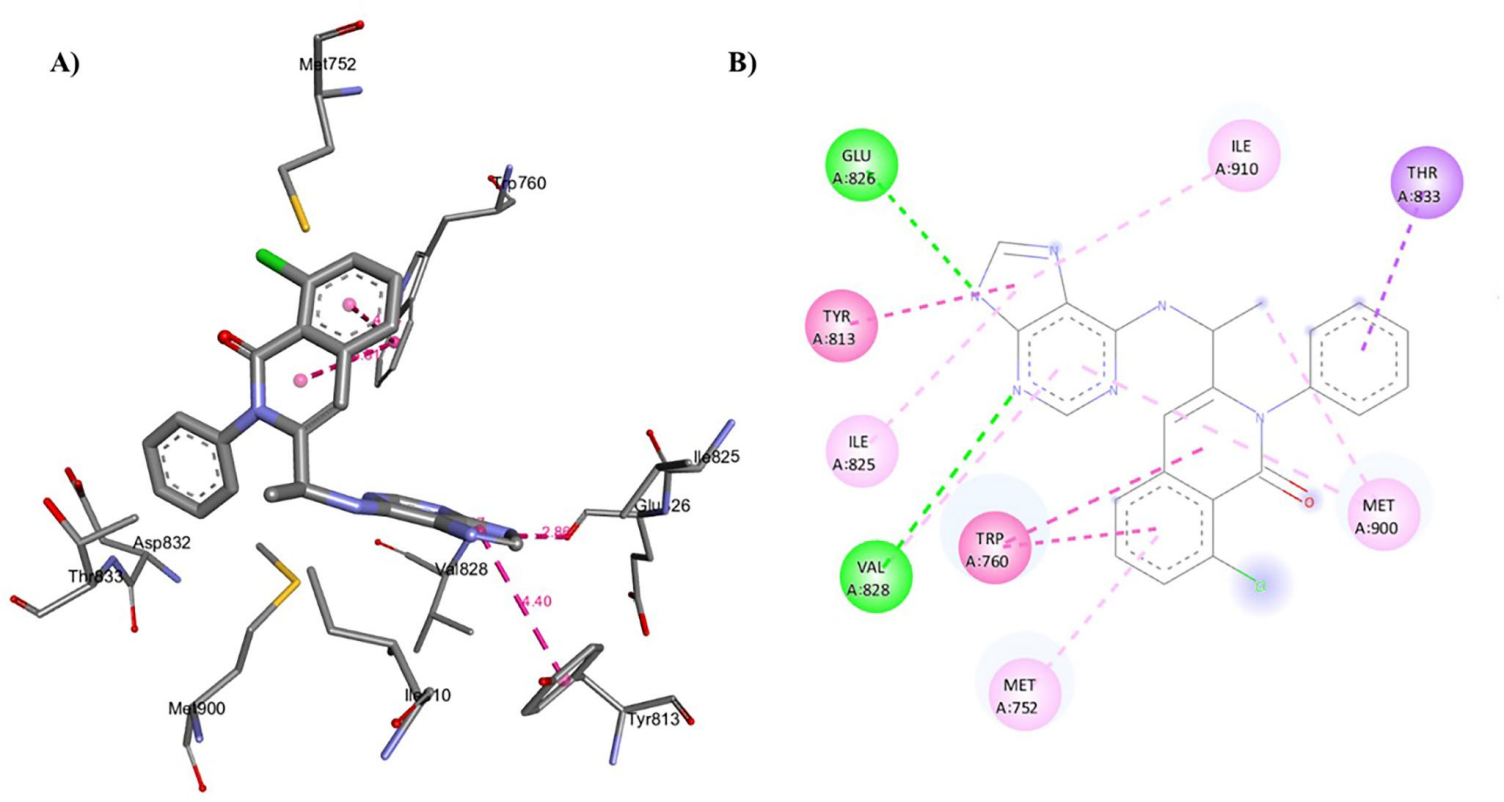
A) 3D structure of the docked duvelisib into the biding site of PI3Kδ (red dotted lines are non-covalent interactions); B) 2D representation of the non-covalent interactions between duvelisib and binding site amino acid residues (green dotted line: H-bonds, other coloured dotted lines: hydrophobic interactions).

It was found that the modelled structure nicely overlays on the co-crystallised structure. We also performed docking for the clinical drug duvelisib into the binding pocket of human PI3Kδ and found that the binding interactions formed are similar to that of the co-crystallised inhibitor (Figure 14).

Duvelisib forms multiple non-covalent interactions with the binding site amino acid residues (Figure 14b). The docking of duvelisib resulted in 9 conformers with affinity ranging from −7.0 to −8.1 kcal/mol. Duvelisib forms several non-covalent interactions with the PI3Kδ amino acid residues (Figure 14a andb): 1) H-bonds with Glu826 (2.86 Å) and Val828 (3.17 Å); 2) Hydrophobic interactions with Met752, Trp760, Tyr813, Ile825, Glu826, Tyr833, Met900 and Ile910.

**Figure 15.**
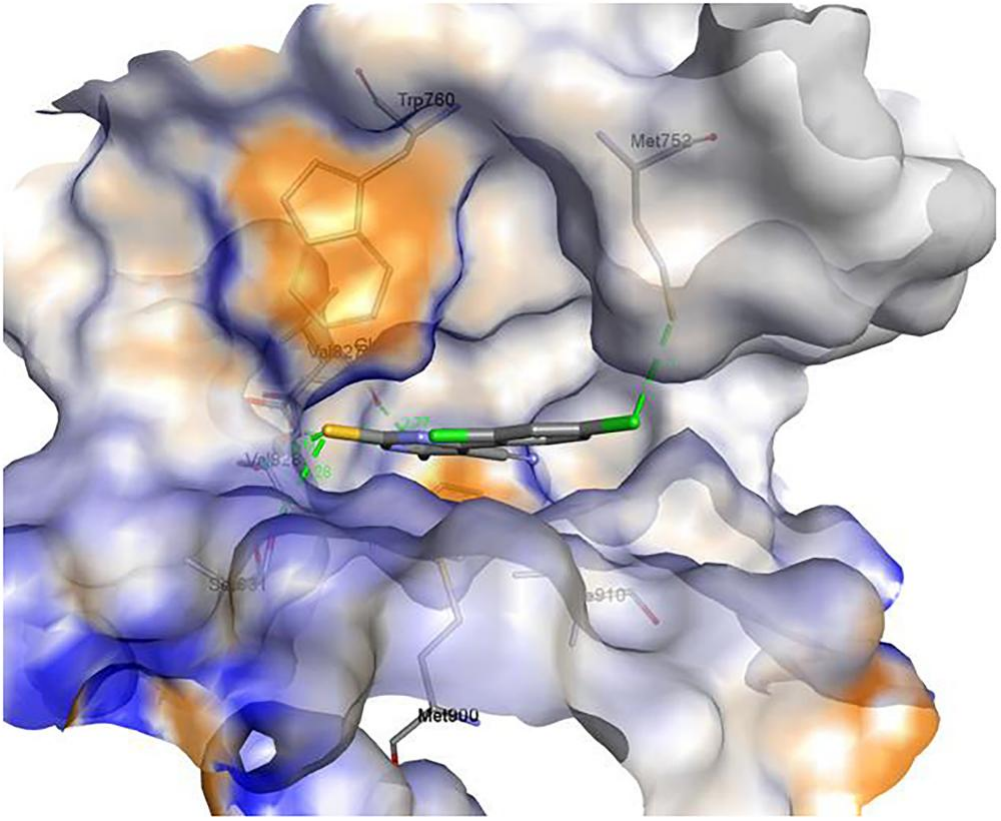
Binding mode of compound **1c** inside the binding pocket of human PI3Kδ.

Similarly, the docking of compound **1c** into the binding pocket of human PI3Kδ (Figure 15) resulted in 9 conformers with affinity ranging from −7.4 to −8.4 kcal/mol.

**Figure 16.**
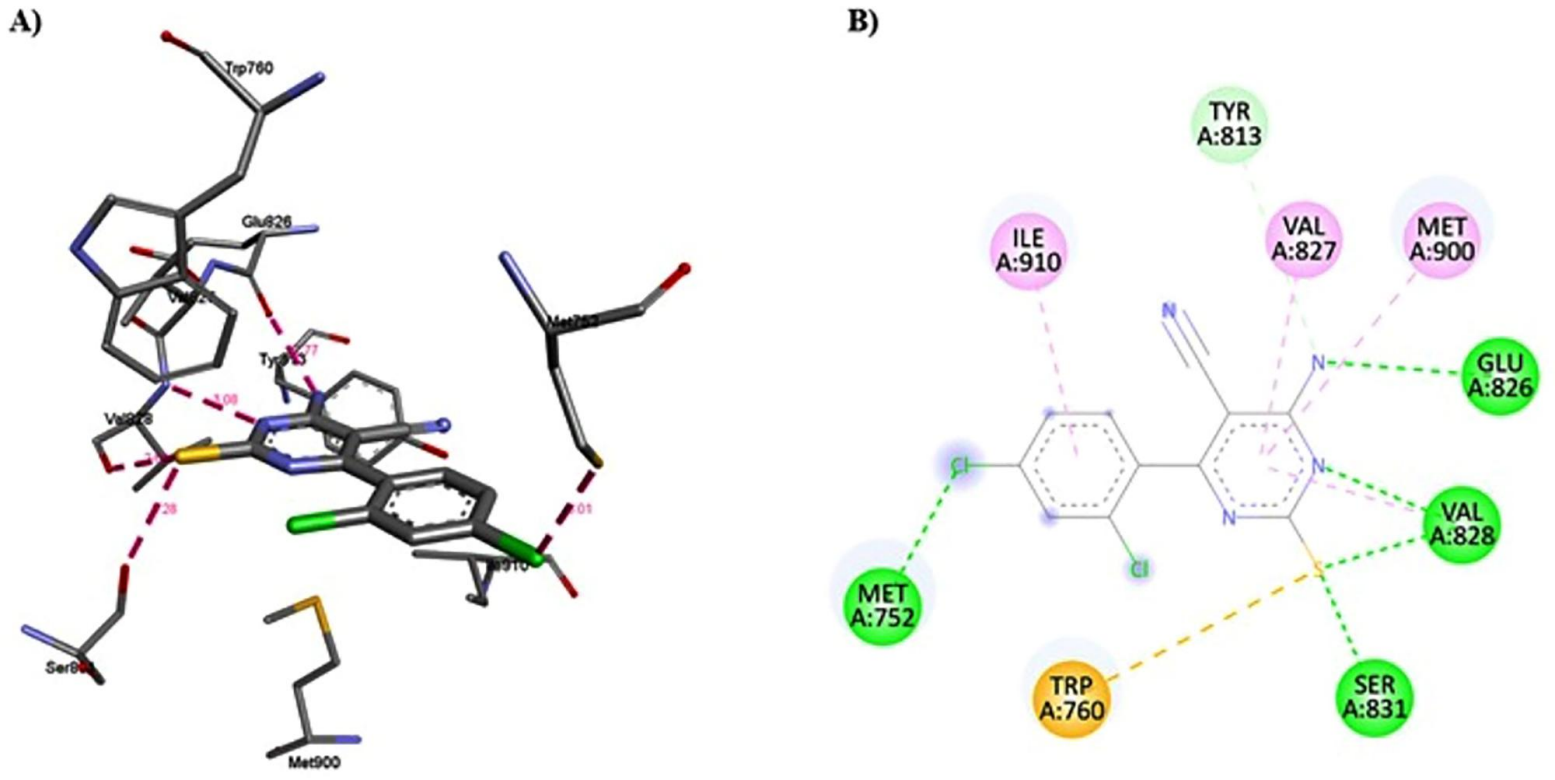
A) 3D structure of the docked compound 1c into the biding site of PI3Kδ (red dotted lines are H-bonds); B) 2D representation of the non-covalent interactions between 1c and binding site amino acid residues (green dotted line: H-bonds, pink dotted lines: hydrophobic interactions; orange line: π-Sulfur interaction).

Compound **1c** forms several non-covalent interactions, i.e. H-bonds and hydrophobic interactions, with the PI3Kδ amino acid residues (Figure 16). Compound **1c** forms multiple H-bonds with Met752 (3.01 Å), Glu826 (2.77 Å), Val828 (3.08 Å and 2.60 Å) and Ser831 (3.28 Å). Additionally, compound **1c** forms multiple hydrophobic interactions with Met752, Tyr813, Glu826, Val827, Val828, Ser831 and Met900. It is notable that compound **1c** binds very well to PI3Kδ with binding affinity compared to Duvelisib and is expected to inhibit its function and hence may have anticancer activity.

## Conclusions

In our effort to develop potent anti-cancer agents, Diverse sets of 6-amino-5-cyano-4-aryl-2-thio pyrimidines and condensed pyrimidines analogues **1a-c, 2a-c, 3a-c, 4a-r and 5a-c)** were designed and synthesised. All compounds were evaluated for *in vitro* anticancer activity by the National Cancer Institute (NCI; MD, USA) against 60 cell lines. The results revealed that compound **1c** has significant broad spectrum anticancer activity against the nine cancerous subpanels tested with GI_50_ (MGMID) and TGI (MG-MID) values of −5.17 μM and −4.48 μM, respectively. Regarding sensitivity of individual cell lines, compound **1c** displayed distinguished sensitivity profile towards leukaemia cell lines with GI_50_ range of 0.08–0.7 μM. Compound **1c** recorded high selectivity towards leukaemia with selectivity ratios value of 39. Compound **1c** possesses anticancer potency higher compared to **1a** and compound **1b**. As evidenced by pyrimidine analog **1c**, the anticancer potency of the pyrimidine are improved by the addition of a more electronegative and lipophilic substituent. The probable mechanism underlying the anticancer activity of the promising compound **1c** was investigated. Compound **1c** arrested cell cycle at S phase and displayed significant increase in the early and late apoptosis in HL60 and leukaemia SR cells. The necrosis percentage showed a significant increase from 1.13% to 3.41% in compound **1c** treated HL60 cells as well as from 1.51% to 4.72% in compound **1c** treated leukaemia SR cells. Compound **1c** showed potent inhibitory activity against PI3Kα, β and δ at sub micromolar concentrations (IC_50_ = 0.88, 0.55, and 0.0034 μM, respectively). Compound **1c** showed comparable activity to Duvelisib against PI3Kδ (IC_50_ = 0.0034 and 0.0025 μM, respectively). Also, compound **1c** triggered apoptosis by activating caspase 3, Bax, P53 and suppressing Bcl_2_. Finally, compound **1c** had low cytotoxic effect on normal lung fibroblast cells (IC_50_ = 43.6 ± 1.20 μM) and was more safe than the clinically used anticancer drug erlotinib (IC_50_ = 22.80 ± 0.65 μM). The molecular modelling study performed identified another possible mechanism of action for these compounds. Duvelisib forms multiple non-covalent interactions with the binding site amino acid residues (Figure 14b). The docking of duvelisib resulted in 9 conformers with affinity ranging from −7.0 to −8.1 kcal/mol. Similarly, the docking of compound **1c** into the binding pocket of human PI3Kδ resulted in 9 conformers with affinity ranging from −7.4 to −8.4 kcal/mol. Compound **1c** binds nicely and forms multiple non-covalent interactions with the binding site amino acid residues. Compound **1c** forms multiple H-bonds with Met752 (3.01 Å), Glu826 (2.77 Å), Val828 (3.08 Å and 2.60 Å) and Ser831 (3.28 Å). Additionally, compound **1c** forms multiple hydrophobic interactions with Met752, Tyr813, Glu826, Val827, Val828, Ser831 and Met900. It is notable that compound **1c** binds very well to PI3Kδ and is expected to inhibit its function and hence may have anticancer activity.

In conclusion, compound **1c** can be considered as lead for further development of new thiopyrimidines for more potent and selective anticancer drugs in the future.

## Supplementary Material

Supplemental Material
