# Supplementary material for: Design, synthesis, molecular modelling and biological evaluation of novel 6-amino-5-cyano-2-thiopyrimidine derivatives as potent anticancer agents against leukemia and apoptotic inducers
Source: J Enzyme Inhib Med Chem. 2024 Feb 13;39(1):2304625. doi: 10.1080/14756366.2024.2304625 (PMC10866072; doi:10.1080/14756366.2024.2304625)
Supplement: Supplemental Material [file IENZ_A_2304625_SM4803.pdf]

***In vitro* PI3 kinases inhibitory activity assay**

The inhibitory activity of compound **1c** was evaluated against PI3K (α, β and δ) using PI3 Kinase Activity/Inhibitor Assay Kit (CAT. # 17-493; Millipore Corporation, MA, USA) according to manufacturer's regulations (see supplementary).

All buffers, working standards and samples were prepared. The kinase enzyme and its inhibitors (Duvelisib and compound **1c**) were preincubated for 10 min before addition of PIP2 (phosphatidylinositol (4,5)-bisphosphate) substrate. A total of 5 µl/well 5X kinase reaction buffer was added following the addition of the substrate, then distilled water was added to fill up to 25 µl. After incubation for 1 h at room temperature, 25 µl of biotinylated PIP3/EDTA working solution was added to all wells except the buffer control. Instead, 25 µl tris-buffered saline (TBS) was added to the buffer control wells. A general receptor phosphoinsitides-1 working solution was added to all wells then, was incubated for 1 h at room temperature. After incubation, all wells were washed with 200 µl 1X TBS-Tween followed by addition of 50 µl (horseradish peroxide as econjugated streptavidin) SA-HRP then were incubated for 1 h at room temperature. All wells were washed using 100 µl 1X TBS-Tween and TBS followed by addition of 100 µl of substrate 3,3',5,5'-tetramethylbenzidine and kept in dark for 5–20 min. Finally, 10 µl stop solution was added and the blue color was assayed at 450 nm

Table 1S: One dose mean graph results of compounds **1a-c and 2a-c** on nine different cancer cell types

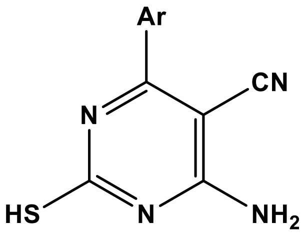

1a-c

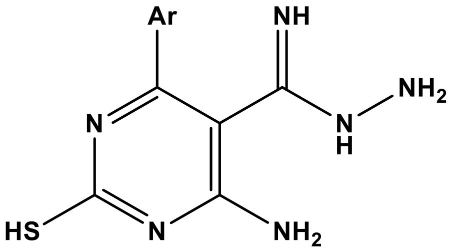

2a-c

Ar= a :4-N$(CH_3)_2$-C_6H_3 , b : 4-Cl-C_6H_4 ,c : 2,4-Cl_2-C_6H_3

**Cell line**	**1a**	**1b**	**1c**	**2a**	**2b**	**2c**
**leukemia**						
CCRF-CEM	90.93	65.47	7.65	91.59	100.09	75.32
HL-60(TB)	94.03	69.64	5.30	99.62	98.64	57.91
K-562	89.77	68.13	36.86	103.46	98.62	91.24
MOLT-4	92.63	50.72	6.11	109.54	99.19	94.43
RPMI-8226	80.50	47.20	9.51	98.02	94.54	87.93
SR	82.96	66.92	2.54	102.70	81.00	69.30
**Non-Small cell lung cancer**						
A549/ATCC	96.51	85.97	90.06	107.34	110.21	89.50
EKVX	87.86	61.03	98.58	95.76	100.22	83.88
HOP-62	116.58	74.60	81.30	107.78	113.92	95.11
HOP-92	94.01	43.53	104.80	114.12	107.66	71.67
NCI-H226	95.99	78.74	64.39	96.50	121.25	90.50
NCI-H23	95.05	85.54	76.08	104.78	107.36	95.68
NCI-H322M	100.72	76.10	96.45	101.58	104.22	97.26
NCI-H460	113.43	86.67	82.63	102.69	107.93	89.85
NCI-H522	79.69	58.67	49.42	100.34	98.92	68.80
**Colon cancer**						
COLO 205	123.45	-	80.54	134.76	-	100.56
HCC-2998	104.67	99.57	83.35	114.82	105.50	108.77
HCT-116	91.91	76.49	-18.77	103.97	119.61	85.00
HCT-15	86.36	85.98	10.91	103.47	103.81	92.01
HT29	109.20	91.07	24.68	107.55	98.85	89.84
KM12	92.30	82.00	49.53	103.89	100.03	100.35
SW-620	113.30	87.27	45.69	105.68	103.87	87.86

Table 1S: One dose mean graph results of compounds 1**a-c and 2a-c** on nine different cancer cell types

**Cell line**	**1a**	**1b**	**1c**	**2a**	**2b**	**2c**
**CNS cancer**						
SF-268	103.74	69.66	51.01	101.57	108.46	104.49
SF-295	103.41	76.16	78.11	112.06	93.17	93.63
SF-539	98.41	71.93	39.87	107.89	103.32	85.28
SNB-19	89.93	60.35	75.37	101.46	98.41	86.22
SNB-75	107.02	42.20	36.51	115.82	99.29	-
U251	90.62	81.58	38.84	106.99	105.90	103.26
**Melanoma**						
LOX IMVI	85.62	79.23	-49.21	101.99	109.11	86.57
MALME-3M	106.34	99.57	4.96	100.97	109.72	94.17
M14	101.88	99.91	49.84	103.05	111.66	95.64
MDA-MB-435	102.70	86.45	25.29	114.53	96.65	96.41
SK-MEL-2	106.70	96.52	98.58	117.18	109.65	95.39
SK-MEL-28	102.57	95.61	57.71	108.97	108.56	109.76
SK-MEL-5	88.72	85.94	58.06	104.41	100.02	97.76
UACC-257	101.53	101.51	77.71	109.40	114.55	102.94
UACC-62	77.19	66.77	62.73	96.91	100.76	84.96
**Ovarian cancer**						
IGROV1	100.43	57.93	65.70	109.01	103.95	104.68
OVCAR-3	94.42	93.44	17.15	105.79	112.26	104.96
OVCAR-4	101.21	76.25	81.25	96.78	103.90	82.22
OVCAR-5	107.61	91.58	93.48	116.97	103.26	98.09
OVCAR-8	103.12	82.25	5.74	116.82	110.09	94.00
NCI/ADR-RES	91.19	-	37.91	104.56	-	103.23
SK-OV-3	121.05	84.27	125.98	120.70	123.58	94.00

Table 1S: One dose mean graph results of compounds 1**a-c and 2a-c** on nine different cancer cell types

Cell line	1a	1b	1c	2a	2b	2c
**Renal cancer**						
786-0	87.88	85.00	66.95	105.86	99.27	90.40
A498	131.00	77.36	136.57	129.90	116.23	105.32
ACHN	98.03	77.19	19.59	99.26	102.02	76.09
CAKI-1	92.37	51.99	72.35	93.33	99.31	83.79
RXF 393	-	55.53	-	-	119.00	94.62
SN12C	90.73	75.71	62.15	106.67	99.89	91.26
TK-10	110.74	126.50	114.14	112.54	113.67	111.85
UO-31	83.81	45.35	63.48	69.66	93.82	71.44
**Prostate cancer**						
PC-3	87.83	56.39	49.77	87.24	111.23	77.58
DU-145	102.09	86.83	51.27	111.10	106.23	107.65
**Breast cancer**						
MCF7	80.41	67.71	31.58	86.35	98.64	79.62
MDA-MB-231/ATCC	96.01	53.73	51.88	103.57	100.62	77.09
HS 578T	100.50	74.01	75.31	111.82	128.54	99.16
BT-549	85.42	-	42.10	106.15	-	81.84
T-47D	75.63	58.29	54.51	99.52	96.25	73.88
MDA-MB-468	60.24	81.12	34.98	117.68	102.27	90.16
**Mean**	96.61	75.67	53.34	105.50	105.07	90.71
**Delta**	36.37	33.47	102.55	35.84	24.07	32.80
**Range**	70.76	84.30	185.78	65.10	47.54	53.94

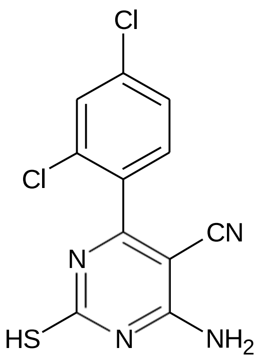

1c

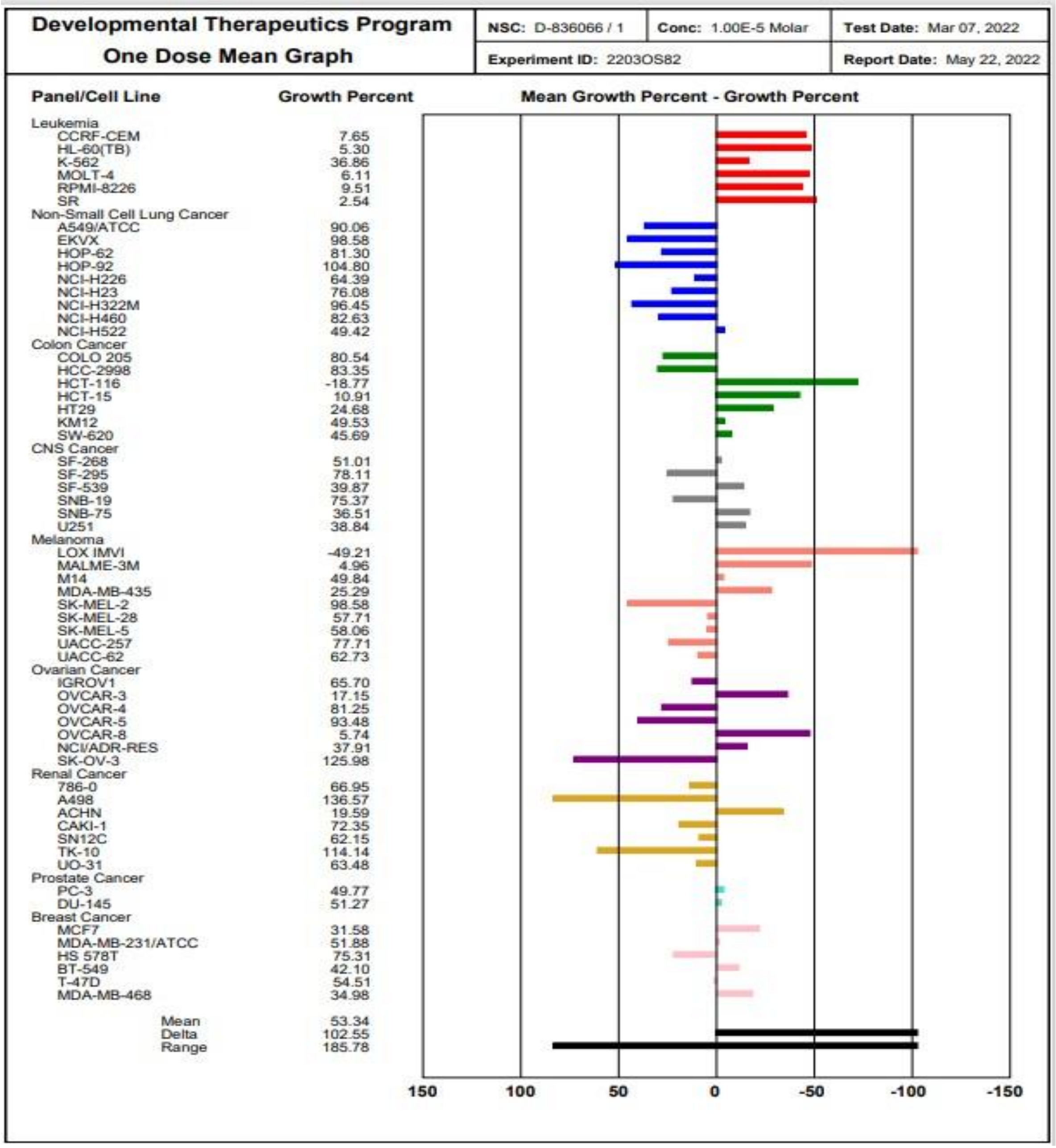

Figure 1: One dose mean graph of nine different cancer cell types of compound 1**c.**

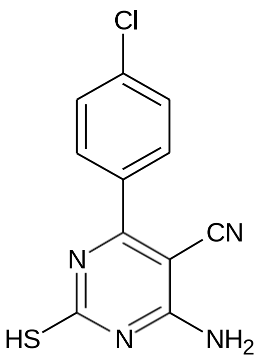

1b

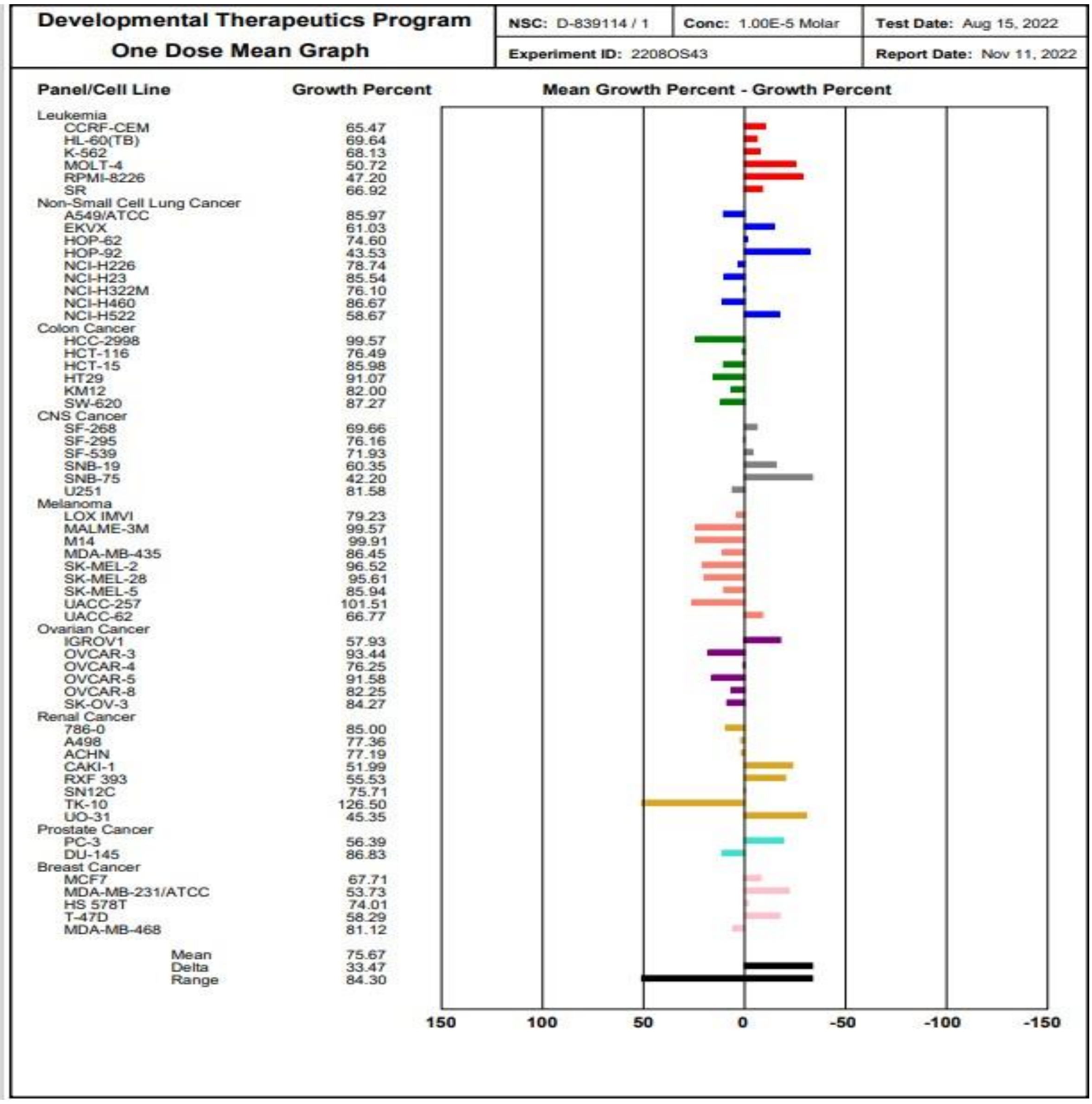

Figure 2: One dose mean graph of nine different cancer cell types of compound 1**b.**

Table 2S: One dose mean graph results of compounds 3**a-c and 5a-c** on nine different cancer cell types

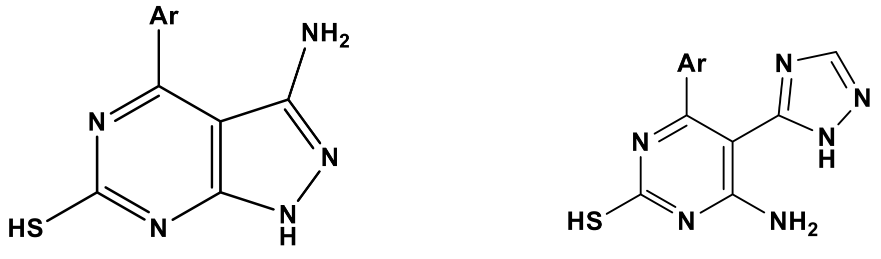

3a-c **5a-c**

Ar= a :4-N(CH_3)$_2$-C_6H_3 , b : 4-Cl-C_6H_4 ,c : 2,4-Cl_2-C_6H_3

**Cell line**	**3a**	**3b**	**3c**	**5a**	**5b**	**5c**
**leukemia**						
CCRF-CEM	77.55	92.49	94.56	102.97	97.93	92.74
HL-60(TB)	89.88	82.04	89.08	84.44	107.84	70.93
K-562	102.48	94.65	105.30	89.79	102.85	98.00
MOLT-4	100.00	92.74	90.06	100.07	107.55	91.44
RPMI-8226	86.49	90.84	108.63	109.14	98.56	102.73
SR	90.80	91.21	97.30	85.71	96.57	83.15
**Non-Small cell lung cancer**						
A549/ATCC	110.12	100.87	104.45	107.70	110.39	101.91
EKVX	97.12	96.85	115.88	120.61	98.05	49.98
HOP-62	108.87	106.23	92.49	102.63	88.64	93.93
HOP-92	116.09	100.46	76.20	100.03	91.67	105.63
NCI-H226	95.19	107.48	106.10	110.37	92.69	109.84
NCI-H23	98.77	103.30	103.34	108.45	97.04	105.26
NCI-H322M	101.18	106.41	106.23	116.16	98.22	102.12
NCI-H460	108.52	107.05	115.81	111.40	109.59	107.59
NCI-H522	92.08	84.30	95.34	101.46	85.51	88.81
**Colon cancer**						
COLO 205	120.31	-	120.01	116.15	-	104.12
HCC-2998	108.59	100.20	103.06	111.87	94.77	105.30
HCT-116	101.11	102.04	112.94	102.30	92.06	97.79
HCT-15	95.53	103.06	111.27	116.48	102.87	102.13
HT29	108.47	95.28	110.65	110.65	95.36	101.86
KM12	97.17	100.80	104.75	105.15	101.65	103.13
SW-620	115.71	102.78	106.47	106.32	102.55	101.83

Table 2S: One dose mean graph results of compounds 3**a-c and 5a-c** on nine different cancer cell types

**Cell line**	**3a**	**3b**	**3c**	**5a**	**5b**	**5c**
**CNS cancer**						
SF-268	105.34	103.53	108.35	106.68	96.70	100.81
SF-295	105.95	89.90	94.52	104.49	93.67	100.46
SF-539	108.53	98.83	94.60	107.58	92.55	101.00
SNB-19	101.52	92.80	93.39	102.16	91.03	97.13
SNB-75	116.67	94.54	-	-	78.72	-
U251	109.49	101.98	103.99	110.97	98.80	104.98
**Melanoma**						
LOX IMVI	97.52	103.05	106.21	109.45	99.17	104.25
MALME-3M	102.71	100.39	92.32	96.70	92.59	98.20
M14	99.09	107.99	106.66	104.80	98.88	98.72
MDA-MB-435	109.49	98.24	110.71	112.96	103.15	104.72
SK-MEL-2	111.64	108.20	108.60	115.73	107.75	103.55
SK-MEL-28	112.69	107.34	115.84	119.33	111.48	114.86
SK-MEL-5	104.39	95.82	108.42	107.98	98.71	94.82
UACC-257	110.00	106.44	107.51	112.85	112.16	112.41
UACC-62	97.30	100.93	93.81	101.84	95.93	95.31
**Ovarian cancer**						
IGROV1	104.23	96.70	105.59	109.06	98.67	102.40
OVCAR-3	116.36	113.31	109.08	111.06	108.35	108.57
OVCAR-4	103.10	98.54	105.05	115.33	111.94	105.17
OVCAR-5	107.30	108.35	108.30	119.56	96.67	107.41
OVCAR-8	104.18	104.96	109.69	113.28	94.82	103.41
NCI/ADR-RES	103.45	-	114.09	115.44	-	106.02
SK-OV-3	107.23	106.45	86.72	102.65	85.54	94.21

Table 2S: One dose mean graph results of compounds 3**a-c and 5a-c** on nine different cancer cell types

**Cell line**	**3a**	**3b**	**3c**	**5a**	**5b**	**5c**
**Renal cancer**						
786-0	109.39	99.34	100.98	96.35	107.65	90.71
A498	162.31	109.21	125.09	130.51	108.68	120.42
ACHN	92.14	102.63	106.88	119.37	96.89	108.81
CAKI-1	92.97	93.47	96.86	89.70	82.84	90.75
RXF 393	-	103.75	114.73	113.44	114.81	109.67
SN12C	100.69	97.55	105.03	106.48	93.47	107.03
TK-10	119.65	114.88	146.75	116.56	108.87	114.37
UO-31	58.31	92.69	85.75	93.94	74.56	89.90
**Prostate cancer**						
PC-3	78.25	101.93	96.81	102.26	85.46	95.84
DU-145	116.81	101.49	119.84	115.27	108.92	112.38
**Breast cancer**						
MCF7	88.54	94.30	95.64	96.58	93.76	94.13
MDA-MB-231/ATCC	98.99	98.65	94.87	107.26	93.73	104.14
HS 578T	108.39	111.10	100.24	112.86	110.76	98.63
BT-549	95.32	-	100.27	98.13	-	97.35
T-47D	102.75	86.49	92.59	109.31	86.44	106.25
MDA-MB-468	115.89	106.46	110.26	110.09	100.79	103.88
**Mean**	103.40	100.23	104.17	107.42	98.37	100.39
**Delta**	45.09	18.19	27.97	22.98	23.81	50.41
**Range**	104.00	32.84	70.55	46.07	40.25	70.44

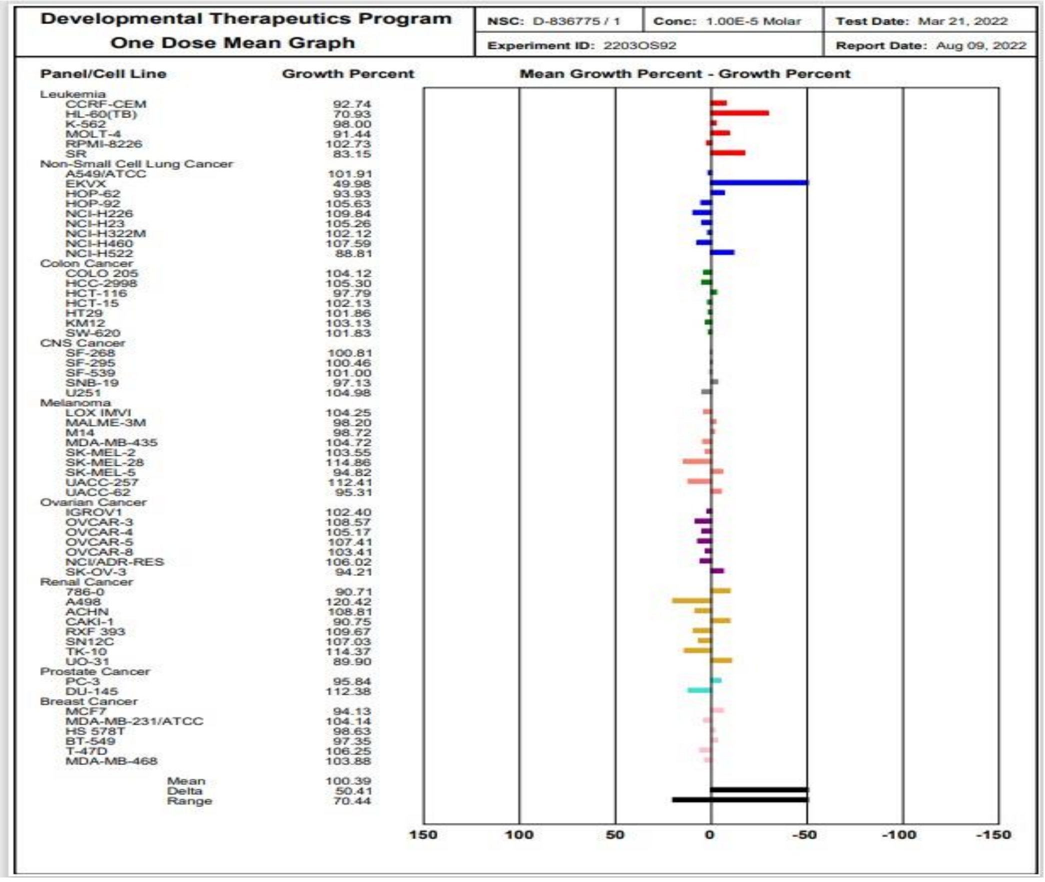

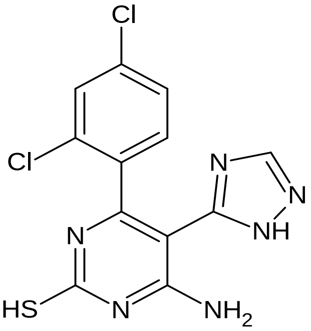

5c

Figure 3: One dose mean graph of nine different cancer cell types of compound 5**c**

Table 3S: One dose mean graph results of compounds **4a-f** on nine different cancer cell types

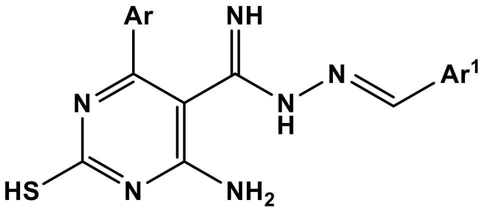

4a-r

Ar= a :4-$N(CH_3)_2$-C_6H_3 , b : 4-Cl-C_6H_4 ,c : 2,4-Cl_2-C_6H_3

Ar^1= C_6H_5 , 4-Cl-C_6H_4 ,4-NO_2-C_6H_4,4-OCH_3-C_6H_4,4-Br-C_6H_4,2,4-Cl_2-C_6H_3

Cell line	4a	4b	4c	4d	4e	4f
**leukemia**						
CCRF-CEM	81.95	76.32	84.44	78.89	88.84	83.70
HL-60(TB)	94.71	91.82	96.42	93.81	91.63	88.37
K-562	106.85	98.52	103.72	100.99	99.60	70.17
MOLT-4	105.98	100.51	108.73	100.86	103.35	100.15
RPMI-8226	89.85	79.12	97.01	86.51	84.44	80.58
SR	104.01	98.69	106.90	102.00	91.54	73.17
**Non-Small cell lung cancer**						
A549/ATCC	102.92	98.44	104.07	103.00	107.76	107.24
EKVX	96.27	91.79	100.43	98.96	98.63	101.98
HOP-62	108.90	109.27	102.11	105.97	108.09	102.77
HOP-92	114.07	117.87	118.01	108.85	112.39	105.72
NCI-H226	98.62	99.76	112.04	102.55	95.73	95.54
NCI-H23	104.33	100.97	103.75	102.87	99.28	99.57
NCI-H322M	99.21	98.53	101.53	95.69	98.15	95.53
NCI-H460	104.21	99.83	107.95	106.33	107.42	116.42
NCI-H522	99.42	92.43	95.97	93.34	98.52	97.15
**Colon cancer**						
COLO 205	139.73	137.33	126.74	117.72	160.99	166.33
HCC-2998	112.86	94.30	115.10	107.52	111.82	115.09
HCT-116	110.41	100.34	106.02	108.11	101.49	67.74
HCT-15	99.88	92.33	98.47	98.16	102.14	76.68
HT29	101.44	92.68	108.78	100.42	103.99	107.33
KM12	107.33	92.90	100.03	93.21	110.12	77.55
SW-620	107.76	102.34	108.52	102.62	106.75	100.23

Table 3S: One dose mean graph results of compounds 4**a-f** on nine different cancer cell types

**Cell line**	**4a**	**4b**	**4c**	**4d**	**4e**	**4f**
**CNS cancer**						
SF-268	104.05	97.45	87.84	96.98	101.01	100.15
SF-295	104.33	96.81	92.44	104.12	104.27	92.69
SF-539	106.25	97.99	98.82	108.17	101.35	81.61
SNB-19	101.85	98.81	97.15	100.57	99.42	90.42
SNB-75	101.34	95.23	100.20	101.44	113.52	109.05
U251	110.80	106.41	106.05	105.75	111.78	93.17
**Melanoma**						
LOX IMVI	98.34	102.59	99.28	100.90	98.62	83.27
MALME-3M	104.73	98.71	108.21	98.40	98.58	96.75
M14	105.58	96.86	109.70	96.11	98.92	102.83
MDA-MB-435	99.40	96.15	101.97	96.68	101.17	91.76
SK-MEL-2	107.94	107.25	113.82	109.48	106.71	108.40
SK-MEL-28	105.45	102.55	112.01	104.92	103.86	104.07
SK-MEL-5	103.30	97.57	102.98	102.45	101.48	99.34
UACC-257	106.19	99.66	105.95	105.89	108.61	108.57
UACC-62	97.92	93.98	99.97	94.28	94.03	92.33
**Ovarian cancer**						
IGROV1	111.29	102.40	102.03	103.83	101.55	101.99
OVCAR-3	101.72	102.50	105.95	105.49	110.26	110.19
OVCAR-4	100.80	95.53	101.30	96.77	101.82	104.31
OVCAR-5	111.29	109.18	109.57	102.97	104.01	106.65
OVCAR-8	115.41	106.29	95.81	103.63	113.27	106.22
NCI/ADR-RES	103.77	104.27	101.63	107.35	102.34	101.46
SK-OV-3	118.49	120.23	95.66	109.56	124.39	125.43

Table 3S: One dose mean graph results of compounds **4a-f** on nine different cancer cell types

**Cell line**	**4a**	**4b**	**4c**	**4d**	**4e**	**4f**
**Renal cancer**						
786-0	101.81	86.71	95.71	98.32	103.21	84.91
A498	106.92	92.06	123.93	119.36	117.60	113.83
ACHN	101.40	101.77	102.79	103.06	104.85	106.71
CAKI-1	99.05	101.56	99.59	97.54	99.59	101.73
RXF 393	-	-	-	-	-	-
SN12C	102.99	88.52	99.46	100.38	102.04	93.73
TK-10	104.52	89.96	102.06	102.54	114.64	113.66
UO-31	75.03	65.36	75.44	71.96	69.94	70.24
**Prostate cancer**						
PC-3	85.08	91.47	94.11	88.46	90.99	89.85
DU-145	103.76	100.17	107.81	106.02	108.82	111.44
**Breast cancer**						
MCF7	93.13	90.87	92.00	95.17	93.86	75.34
MDA-MB-231/ATCC	109.11	102.05	111.21	103.30	101.51	99.54
HS 578T	113.39	97.51	95.19	112.13	107.89	96.85
BT-549	103.28	95.72	111.67	102.31	106.96	118.68
T-47D	105.47	88.33	100.81	98.67	97.50	103.62
MDA-MB-468	117.15	106.91	108.19	113.91	110.91	113.07
**Mean**	103.95	98.19	102.97	101.31	103.63	98.86
**Delta**	28.92	32.83	27.53	29.35	33.69	31.12
**Range**	64.70	71.97	51.30	47.40	91.05	98.59

Table 4S: One dose mean graph results of compounds 4 **g-l** on nine different cancer cell types

**Cell line**	**4g**	**4h**	**4i**	**4j**	**4k**	**4l**
**leukemia**						
CCRF-CEM	65.01	75.50	88.62	89.69	43.36	64.57
HL-60(TB)	96.98	100.57	97.27	111.06	119.46	100.95
K-562	52.38	85.96	96.31	95.01	67.82	77.13
MOLT-4	58.49	85.76	104.89	100.58	54.63	66.42
RPMI-8226	51.39	83.79	89.04	91.20	56.81	46.53
SR	46.39	86.82	96.52	97.31	62.42	65.58
**Non-Small cell lung cancer**						
A549/ATCC	31.63	93.14	103.05	107.45	55.33	90.68
EKVX	52.91	75.04	95.81	94.77	66.33	59.03
HOP-62	30.23	95.60	95.02	90.32	97.98	52.98
HOP-92	-0.44	84.18	96.97	107.32	18.97	54.89
NCI-H226	-0.68	98.97	149.72	124.91	98.01	62.40
NCI-H23	64.34	93.69	106.02	102.51	71.83	78.34
NCI-H322M	81.52	87.31	88.86	96.77	82.40	77.26
NCI-H460	16.47	93.73	103.71	119.43	73.40	66.34
NCI-H522	56.35	85.44	92.91	101.36	73.53	62.30
**Colon cancer**						
COLO 205	-	-	-	-	-	-
HCC-2998	93.06	102.93	103.80	113.38	110.76	95.01
HCT-116	30.62	86.05	107.62	106.96	69.63	84.20
HCT-15	53.06	90.39	95.16	95.64	73.38	77.36
HT29	78.50	98.97	100.90	104.91	101.51	86.98
KM12	62.26	91.90	99.20	100.06	76.25	79.85
SW-620	76.33	103.18	102.02	111.99	95.59	95.83

Table 4S: One dose mean graph results of compounds 4**g-l** on nine different cancer cell types

**Cell line**	**4g**	**4h**	**4i**	**4j**	**4k**	**4l**
**CNS cancer**						
SF-268	49.28	96.04	116.84	113.31	90.21	73.58
SF-295	35.61	90.23	96.72	97.51	91.20	84.99
SF-539	11.40	80.42	96.67	101.01	71.25	70.35
SNB-19	59.82	87.50	94.21	90.46	80.38	84.01
SNB-75	-34.30	83.46	115.26	102.67	35.46	26.06
U251	13.22	93.96	98.85	106.78	77.13	93.92
**Melanoma**						
LOX IMVI	22.56	94.29	106.34	104.30	92.34	69.40
MALME-3M	52.08	106.53	96.90	105.03	119.24	43.36
M14	70.64	103.80	107.33	109.48	76.45	76.22
MDA-MB-435	77.29	98.80	100.70	103.72	87.84	88.96
SK-MEL-2	92.71	101.05	108.08	109.51	95.99	83.27
SK-MEL-28	88.55	100.03	101.27	111.48	105.08	75.77
SK-MEL-5	84.86	97.32	101.42	101.98	93.28	80.96
UACC-257	92.82	101.08	96.55	109.70	81.36	96.53
UACC-62	78.12	81.76	94.57	92.91	82.39	43.08
**Ovarian cancer**						
IGROV1	62.56	78.84	99.42	91.47	68.20	44.88
OVCAR-3	77.67	107.04	119.45	110.24	101.27	80.21
OVCAR-4	37.46	83.10	98.60	99.56	76.68	79.21
OVCAR-5	80.08	95.39	101.17	102.03	96.96	73.21
OVCAR-8	20.38	94.83	104.73	100.84	63.73	89.97
NCI/ADR-RES	-	-	-	-	-	-
SK-OV-3	80.96	77.49	102.91	93.73	78.65	82.10

Table 4S: One dose mean graph results of compounds 4**g-l** on nine different cancer cell types

**Cell line**	**4g**	**4h**	**4i**	**4j**	**4k**	**4l**
**Renal cancer**						
786-0	41.67	98.52	102.61	104.04	89.92	88.64
A498	104.62	101.50	113.78	125.31	88.41	6.77
ACHN	23.28	77.16	92.07	97.37	77.28	53.24
CAKI-1	31.24	79.43	89.64	91.91	62.37	43.20
RXF 393	8.96	102.56	108.17	123.54	82.51	37.00
SN12C	73.92	91.85	92.64	93.60	87.67	65.46
TK-10	93.88	121.65	111.67	142.67	115.75	98.77
UO-31	29.45	66.27	90.98	77.48	47.83	31.19
**Prostate cancer**						
PC-3	52.60	79.61	90.11	82.59	70.55	48.02
DU-145	48.70	107.77	107.79	117.57	83.91	79.30
**Breast cancer**						
MCF7	42.23	76.56	90.65	86.57	57.47	53.10
MDA-MB-231/ATCC	1.22	69.72	96.05	91.26	-1.21	52.66
HS 578T	-6.98	92.97	118.44	107.46	42.57	36.43
BT-549	-	-	-	-	-	-
T-47D	49.42	72.07	90.55	86.80	54.82	28.14
MDA-MB-468	49.66	99.27	113.64	113.70	84.63	88.40
**Mean**	50.78	91.03	101.41	102.85	76.82	68.33
**Delta**	85.08	24.76	12.79	25.37	78.03	61.56
**Range**	138.92	55.38	61.10	65.19	120.67	94.18

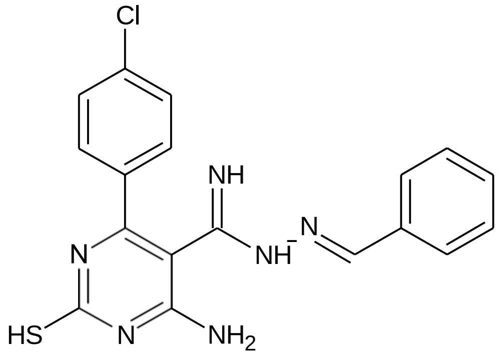

4g

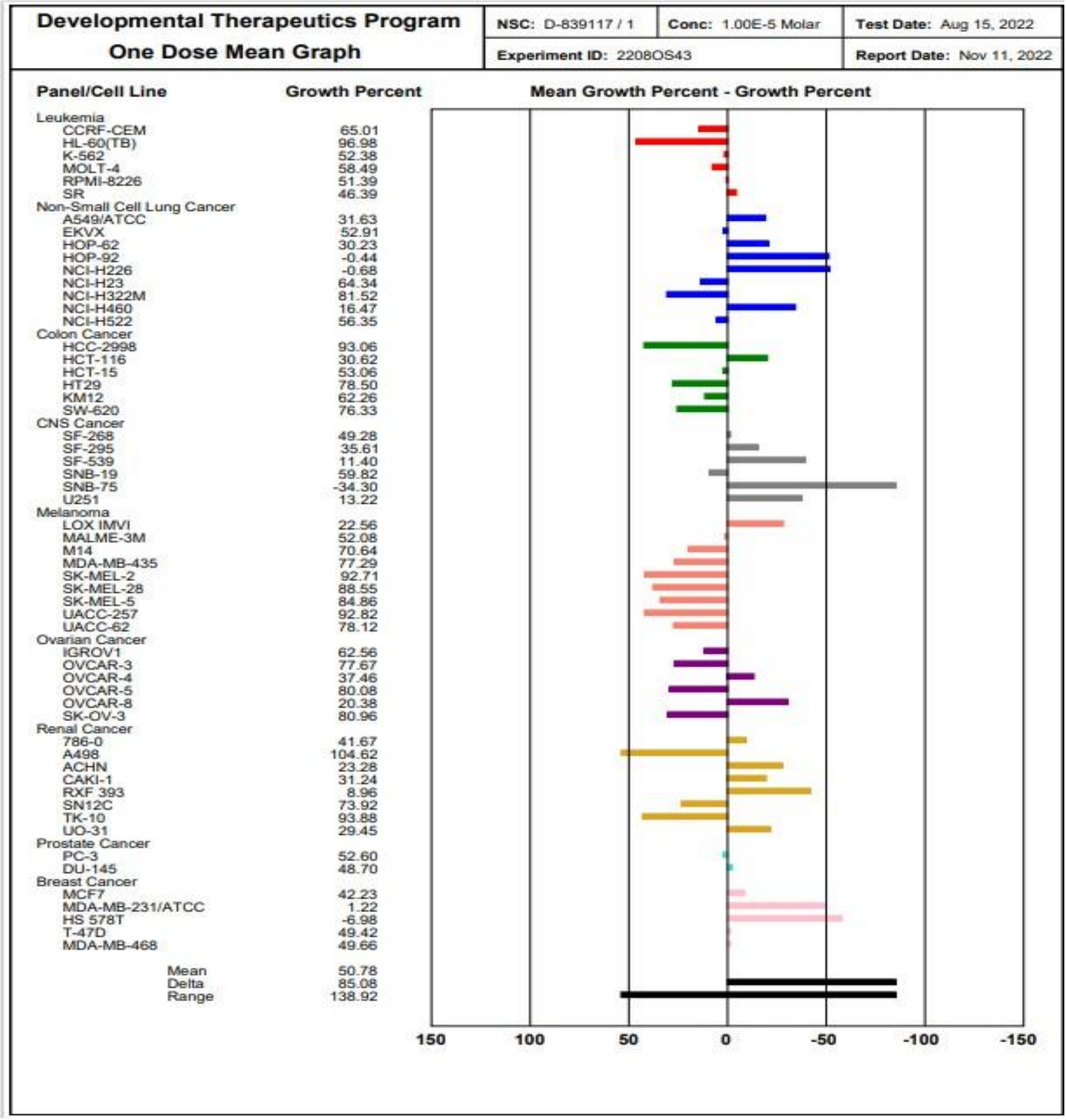

Figure 4: One dose mean graph of nine different cancer cell types of compound **4g**

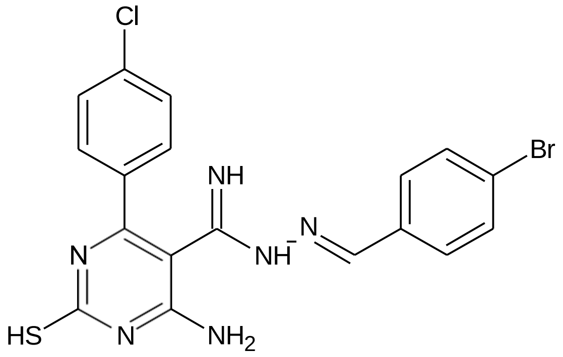

4k

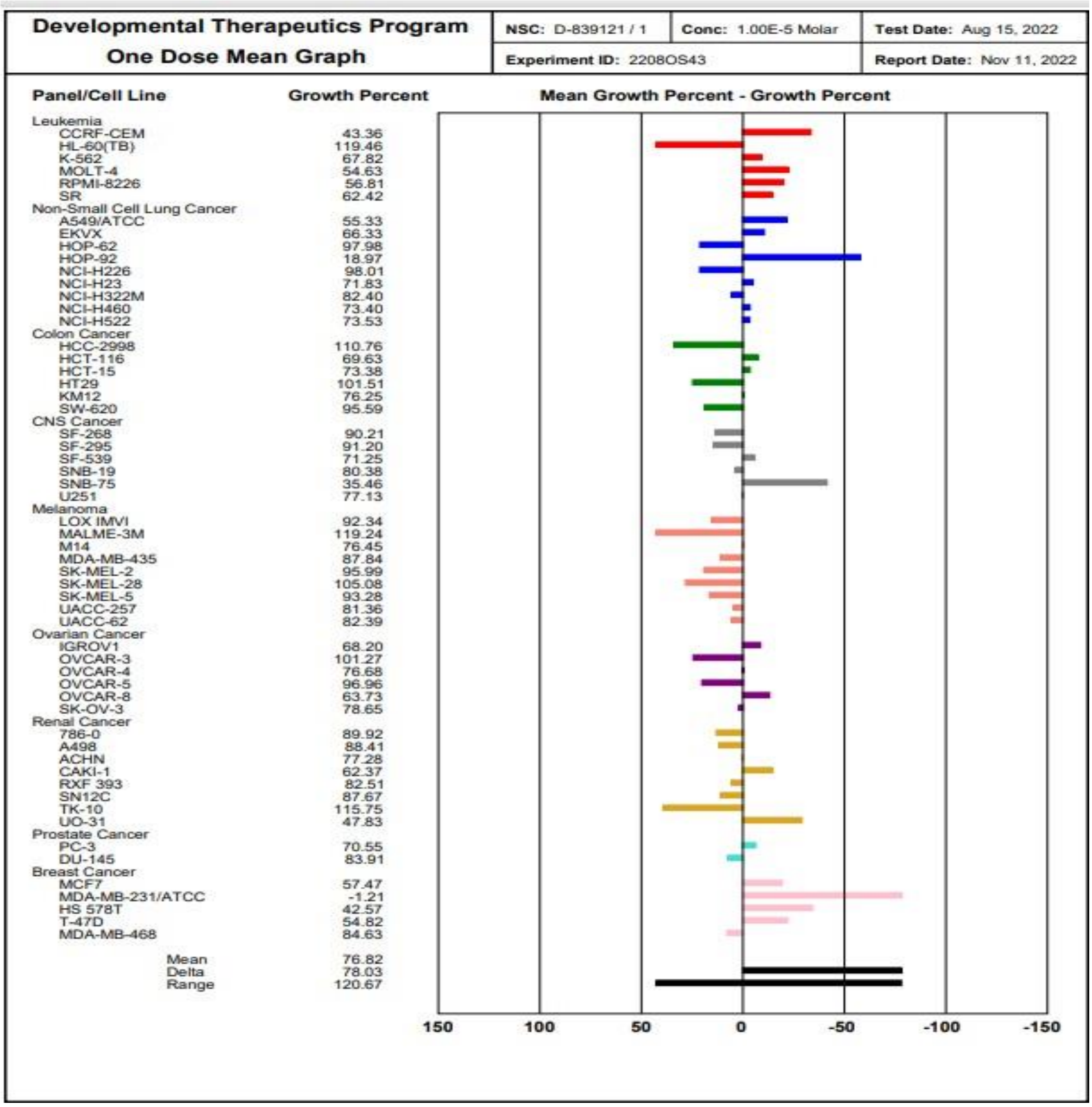

Figure 5: One dose mean graph of nine different cancer cell types of compound **4k**

Table 5S: One dose mean graph results of compounds 4**m-r** on nine different cancer cell types

**Cell line**	**4m**	**4n**	**4o**	**4p**	**4q**	**4r**
**leukemia**						
CCRF-CEM	104.59	105.92	106.20	99.14	96.76	94.91
HL-60(TB)	97.14	107.51	103.25	96.91	96.50	101.59
K-562	95.81	89.92	94.96	102.14	92.22	99.98
MOLT-4	96.18	92.36	94.03	95.65	91.37	100.36
RPMI-8226	104.48	101.85	97.31	99.42	98.77	105.95
SR	83.16	90.64	76.85	97.45	86.67	92.64
**Non-Small cell lung cancer**						
A549/ATCC	97.43	95.78	100.52	102.33	98.75	102.20
EKVX	103.25	108.05	98.91	107.90	107.97	114.37
HOP-62	96.56	83.09	57.00	90.23	100.71	101.82
HOP-92	69.19	64.20	2.04	74.82	102.01	77.97
NCI-H226	92.02	86.35	82.13	97.25	107.41	106.33
NCI-H23	98.69	92.91	90.15	100.02	102.18	103.58
NCI-H322M	99.06	102.25	100.04	104.38	104.03	106.69
NCI-H460	107.69	102.07	113.78	112.77	110.26	110.51
NCI-H522	108.60	90.63	78.53	97.49	100.90	100.38
**Colon cancer**						
COLO 205	115.23	113.67	115.23	126.59	114.39	112.53
HCC-2998	105.00	101.42	106.17	117.91	114.01	100.37
HCT-116	102.91	94.61	85.22	99.76	96.20	102.11
HCT-15	100.98	101.19	102.65	106.06	107.53	112.55
HT29	108.14	120.64	115.91	105.63	112.36	118.19
KM12	103.17	107.50	107.90	103.63	100.05	100.10
SW-620	108.79	106.23	108.21	104.71	107.55	105.23

Table 5S: One dose mean graph results of compounds 4**m-r** on nine different cancer cell types

**Cell line**	**4m**	**4n**	**4o**	**4p**	**4q**	**4r**
**CNS cancer**						
SF-268	105.23	100.45	94.94	104.39	104.94	100.68
SF-295	86.90	73.40	52.43	85.77	96.63	98.50
SF-539	100.14	87.29	74.67	96.75	102.25	101.24
SNB-19	95.76	90.87	74.87	93.00	99.18	94.56
SNB-75	-	-	-	-	-	-
U251	97.35	70.46	66.98	103.30	106.77	104.78
**Melanoma**						
LOX IMVI	100.28	95.36	96.47	103.03	108.96	103.89
MALME-3M	85.67	81.62	72.95	89.40	91.24	86.51
M14	96.25	95.62	101.73	107.06	100.86	97.41
MDA-MB-435	110.10	104.18	107.58	112.97	104.31	113.29
SK-MEL-2	120.36	107.80	93.41	115.27	109.43	111.05
SK-MEL-28	103.54	105.96	112.97	112.29	106.00	113.47
SK-MEL-5	103.06	102.57	104.48	105.47	104.40	107.37
UACC-257	105.61	108.25	104.26	103.42	102.50	105.56
UACC-62	94.27	90.92	84.72	97.23	96.38	96.40
**Ovarian cancer**						
IGROV1	101.26	97.01	94.91	108.05	105.49	102.14
OVCAR-3	108.64	110.05	110.21	112.91	104.21	108.29
OVCAR-4	115.25	106.68	110.90	121.63	109.05	104.32
OVCAR-5	107.87	104.77	111.15	117.20	119.17	116.71
OVCAR-8	108.77	91.16	98.36	103.43	113.54	109.93
NCI/ADR-RES	104.65	104.62	104.73	113.78	114.00	110.83
SK-OV-3	95.57	92.24	89.12	108.27	103.03	104.63

Table 5S: One dose mean graph results of compounds 4**m-r** on nine different cancer cell types

**Cell line**	**4m**	**4n**	**4o**	**4p**	**4q**	**4r**
**Renal cancer**						
786-0	92.45	78.01	54.09	90.17	93.83	87.53
A498	125.44	121.64	159.67	138.08	128.78	117.14
ACHN	108.37	83.69	88.85	109.85	108.06	107.24
CAKI-1	95.95	98.62	78.85	97.30	86.86	108.87
RXF 393	89.48	84.65	73.05	94.52	101.34	118.98
SN12C	95.92	90.07	96.13	108.49	102.75	102.35
TK-10	140.01	143.10	142.20	142.78	148.12	111.83
UO-31	90.14	93.17	98.26	97.72	94.15	92.50
**Prostate cancer**						
PC-3	102.77	102.40	96.77	98.91	102.01	98.06
DU-145	110.88	108.53	114.71	110.28	108.08	107.77
**Breast cancer**						
MCF7	89.14	83.64	88.24	91.49	90.89	98.94
MDA-MB-231/ATCC	94.39	82.55	77.52	90.47	98.49	100.65
HS 578T	102.21	102.01	87.12	104.55	117.92	105.14
BT-549	88.53	92.59	75.65	95.61	97.73	91.58
T-47D	102.94	92.64	95.34	100.37	110.37	103.09
MDA-MB-468	91.07	96.10	90.75	105.26	113.45	110.70
**Mean**	101.09	97.18	93.49	103.94	104.30	103.80
**Delta**	31.90	32.98	91.45	29.12	17.63	25.83
**Range**	70.82	78.90	157.63	67.96	61.45	41.01

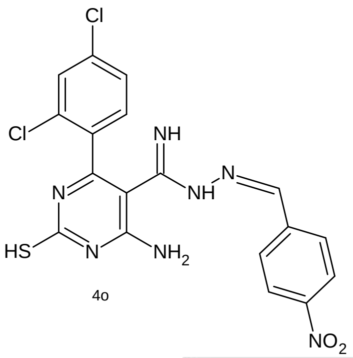

4o

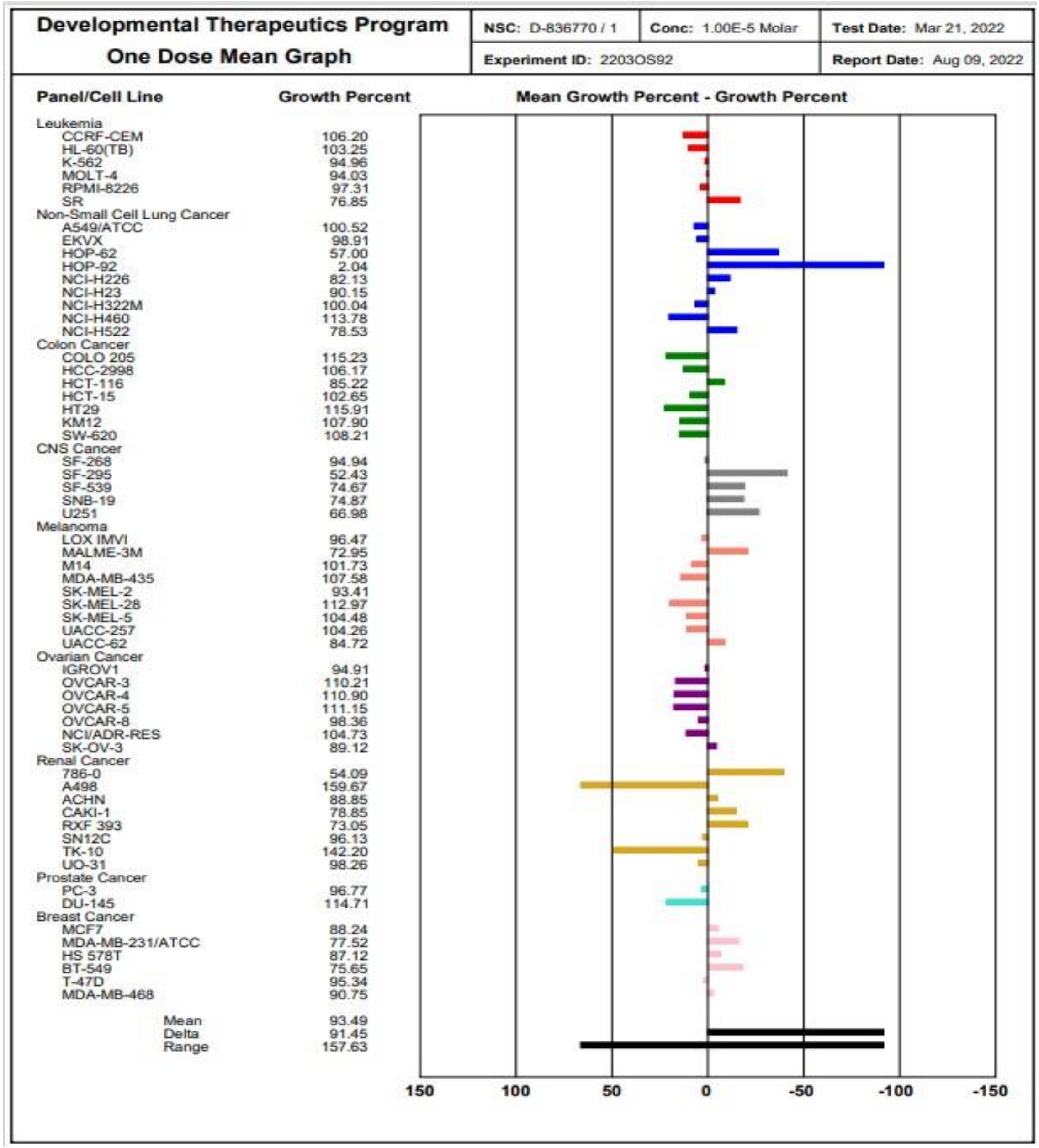

Figure 6: One dose mean graph of nine different cancer cell types of compound 4**o**

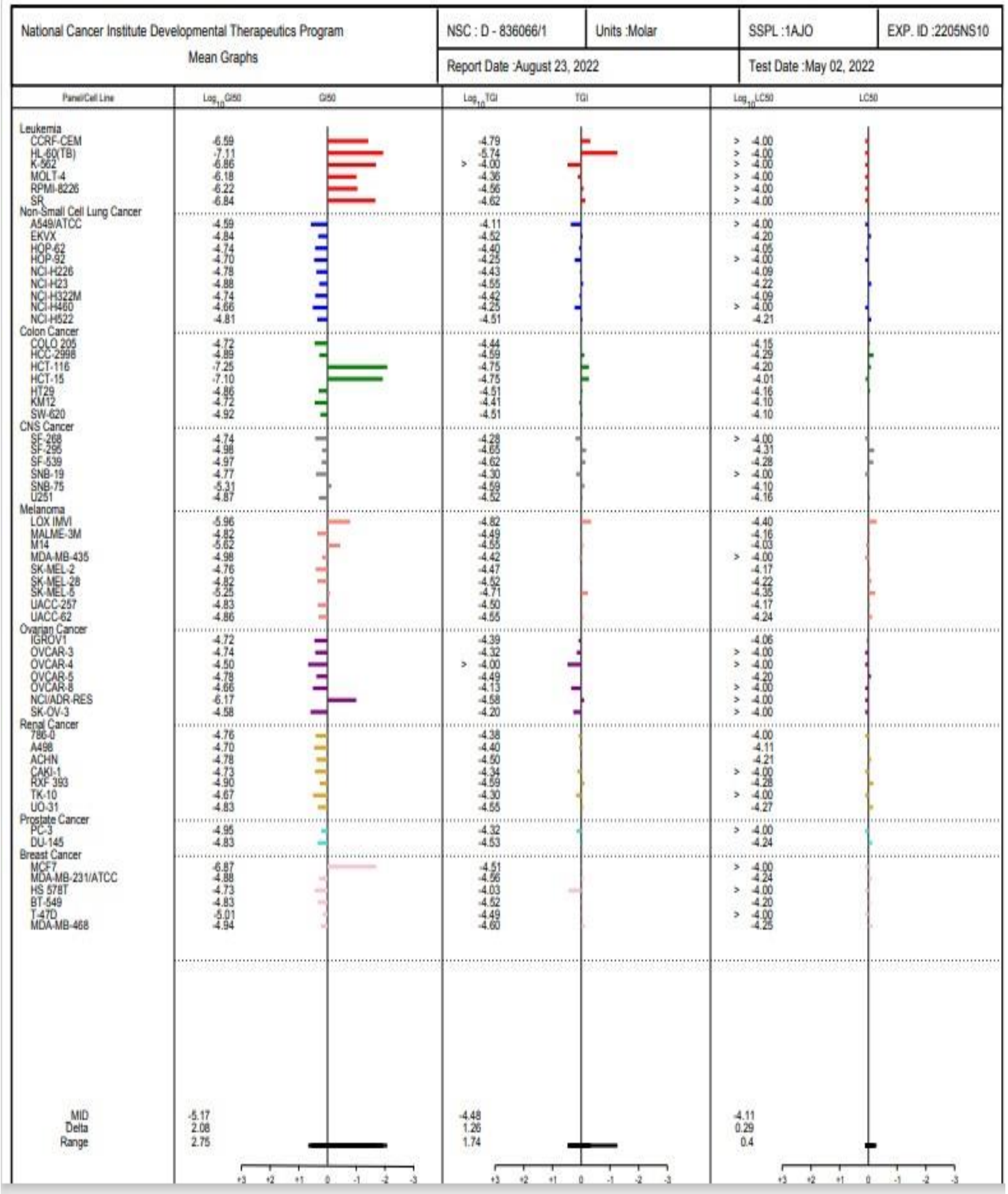

Figure 7: $\log_{10}$ GI_{50}, The $\log_{10}$ TGI and $\log_{10}$ LC_{50} of compound 1**c** against 60 cancer cell lines.

Supplementary data

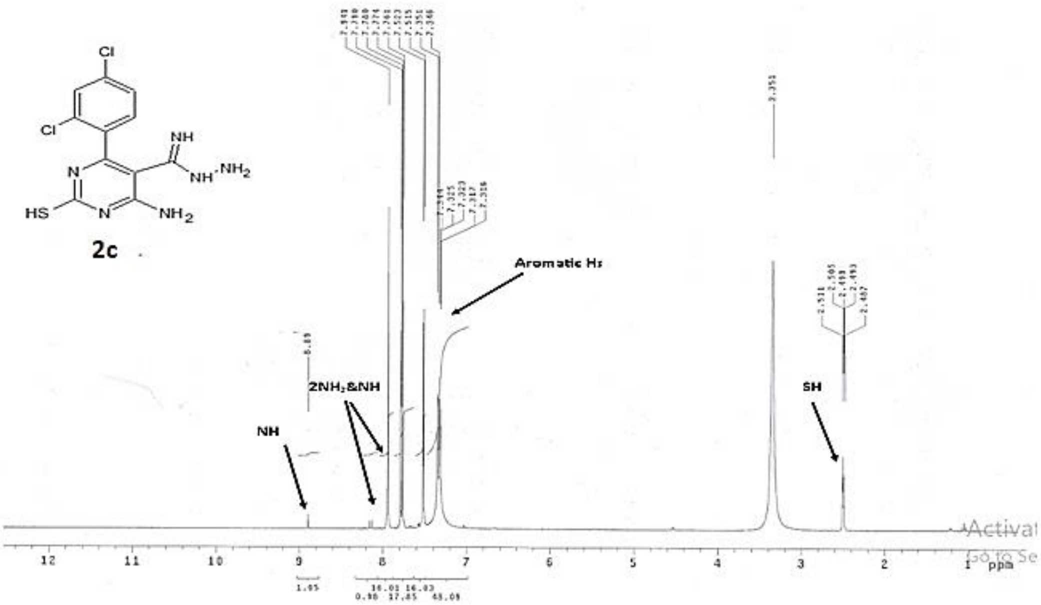

^{1}H-NMR spectrum of compound **2c** DMSO-*d*$_6$**.**

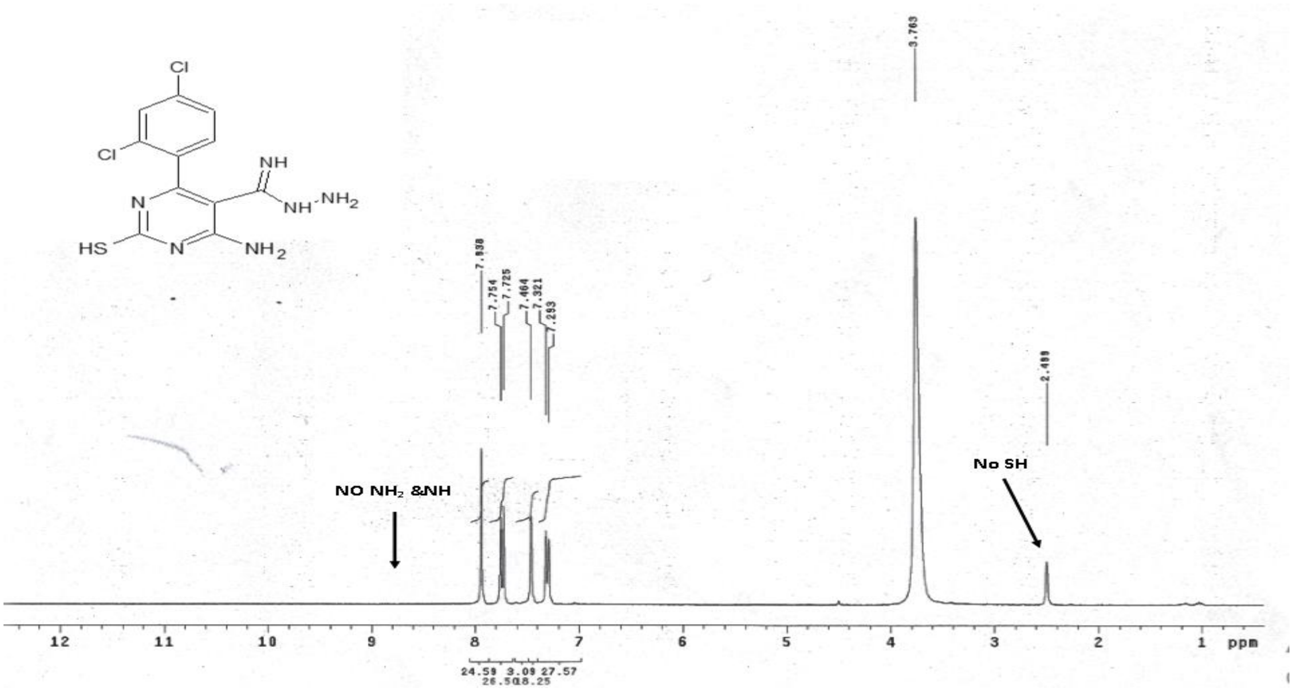

^{1}H-NMR spectrum of compound **2c** D_2O.

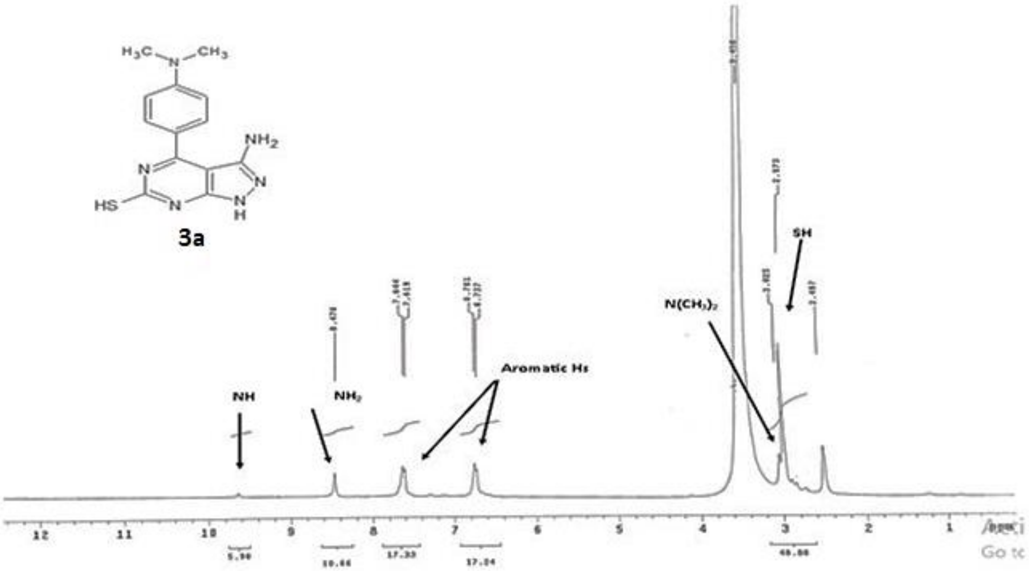

^{1}H-NMR spectrum of compound **3a** DMSO-d_6.

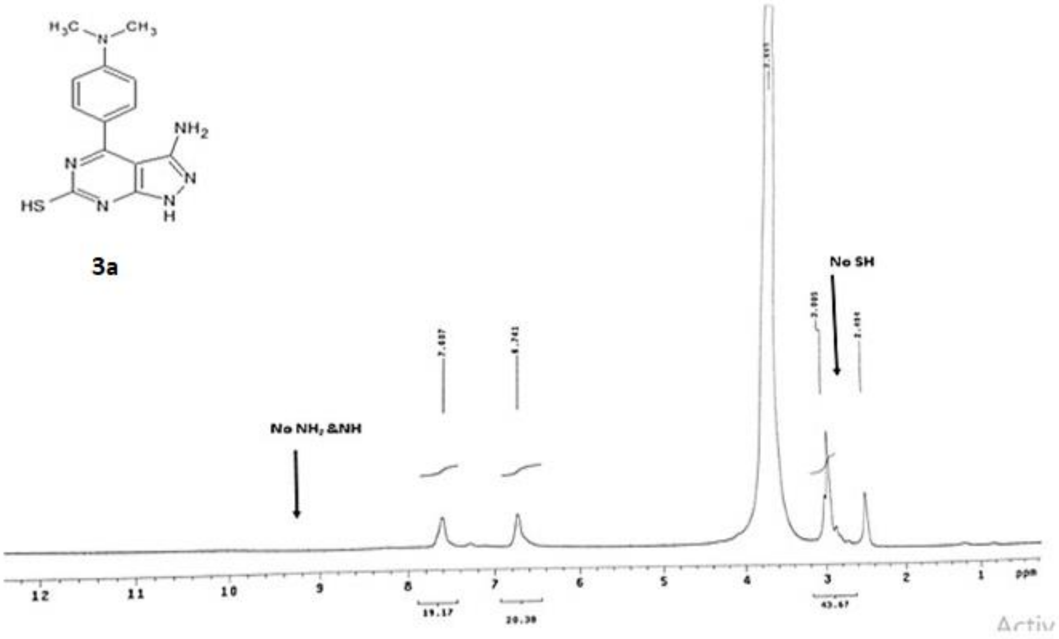

^{1}H-NMR spectrum of compound **3a** D_2O**.**

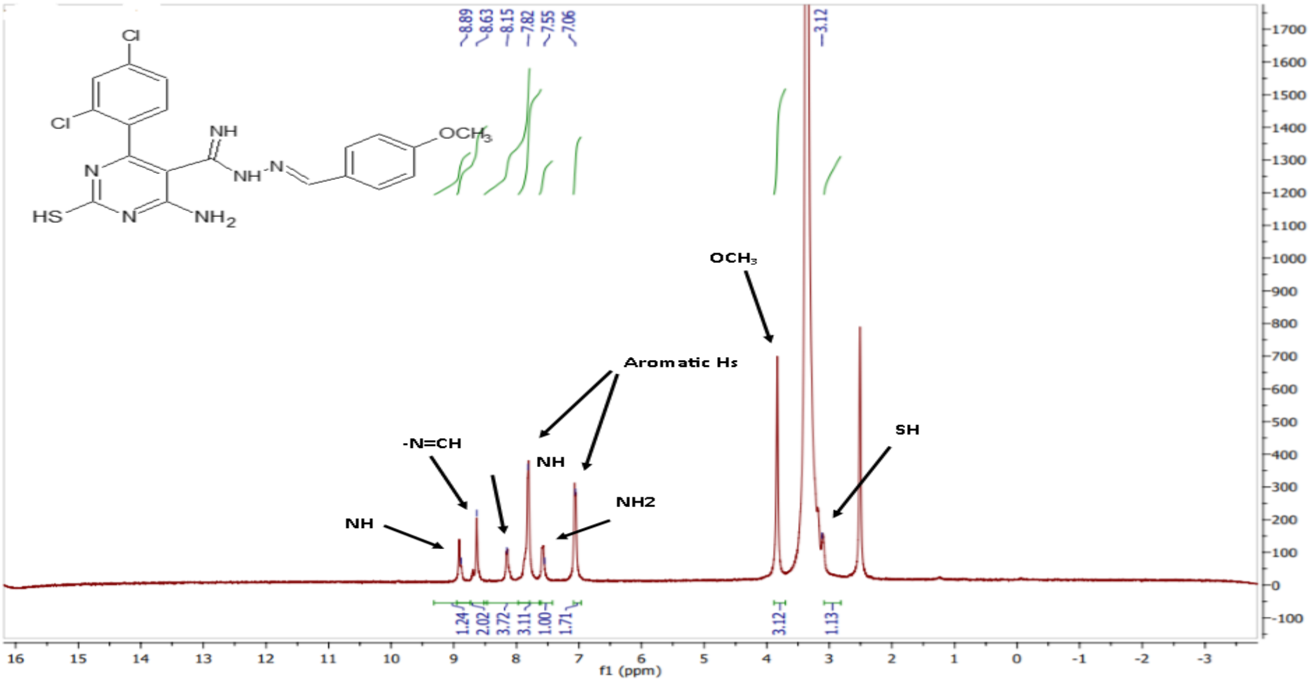

^{1}H-NMR spectrum of compound **4P** DMSO-d_6**.**

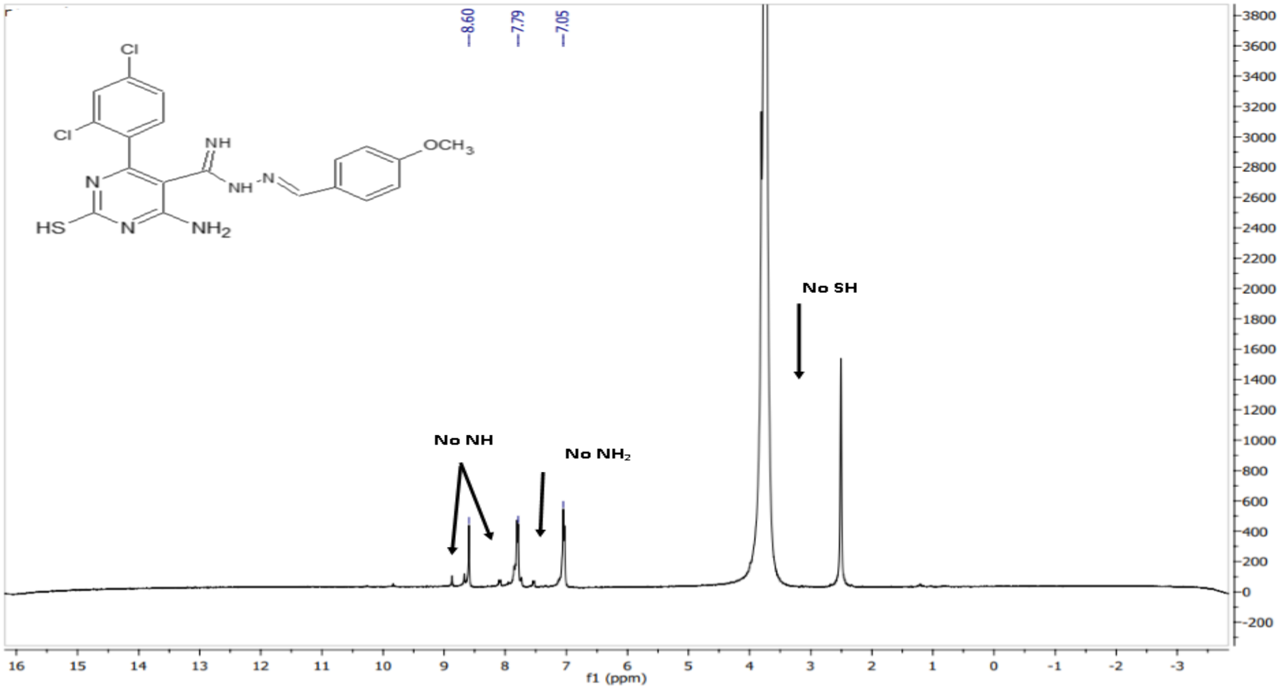

^{1}H-NMR spectrum of compound **4p** D_2O**.**

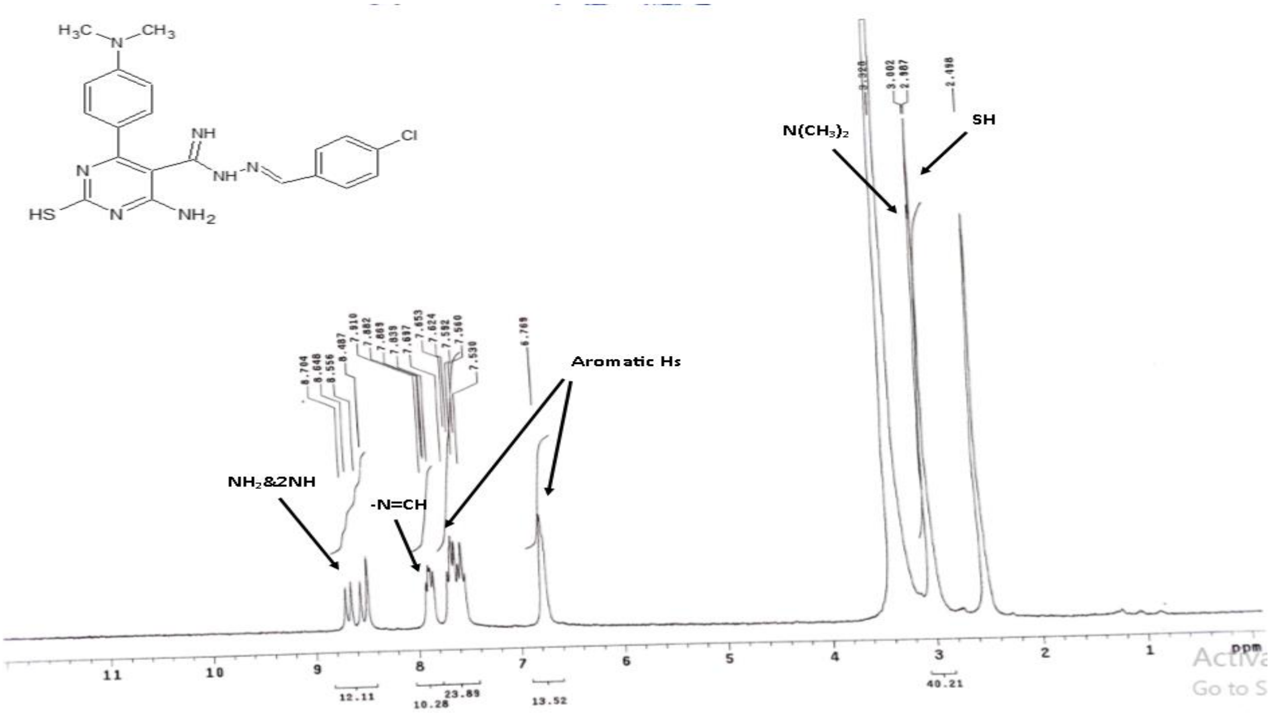

^{1}H-NMR spectrum of compound **4b** DMSO-d_6.

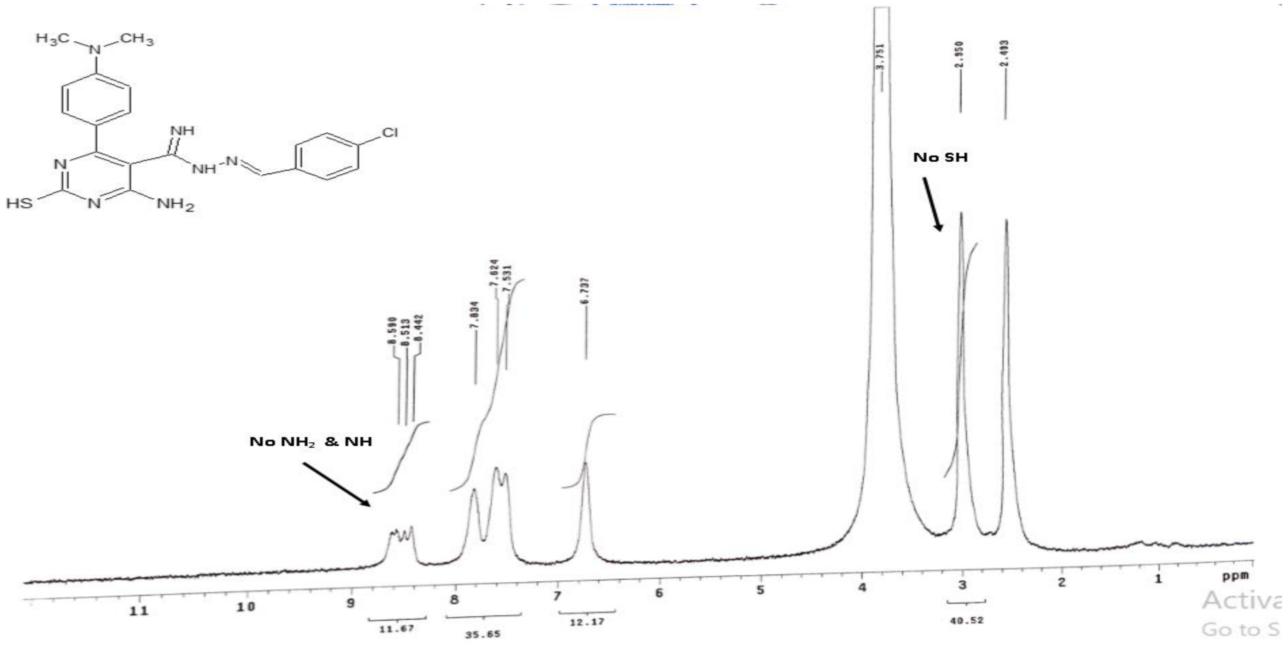

^{1}H-NMR spectrum of compound **4b** D_2O**.**

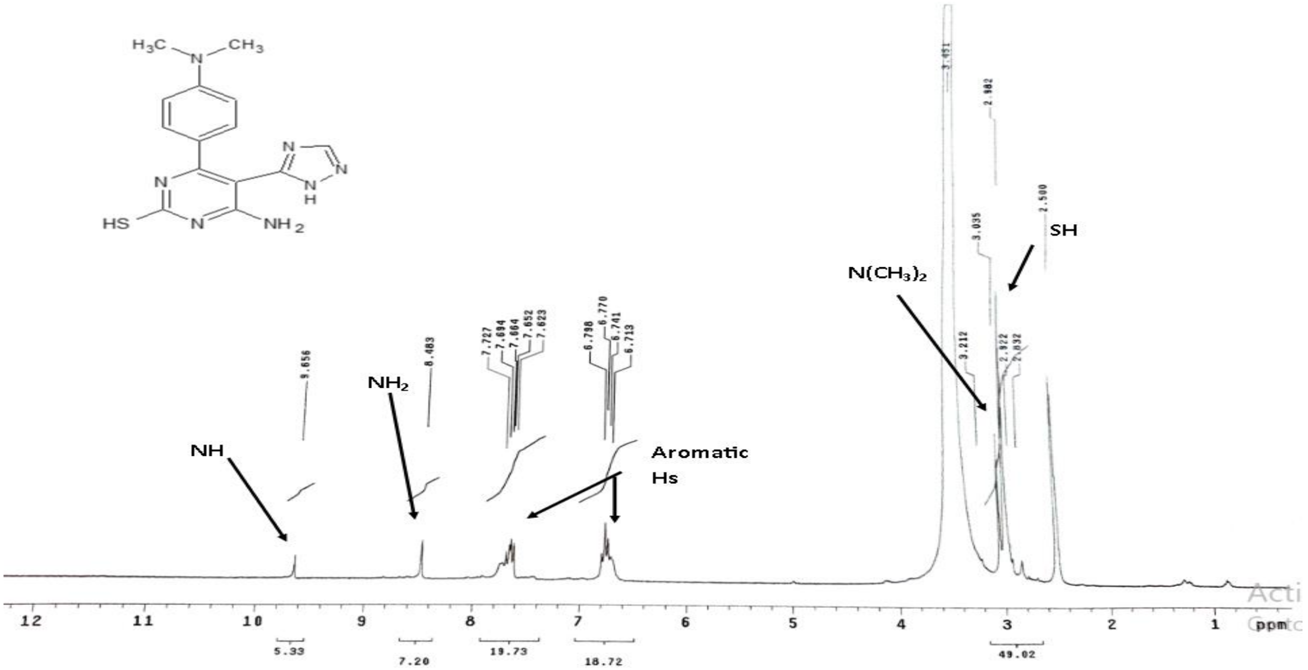

^{1}H-NMR spectrum of compound **5a** DMSO-d_6**.**
